# ﻿A subgeneric revision of the genus *Suillus* (*Suillaceae*, *Boletales*) and novel taxa from Eastern Asia based on morphology and multigene phylogenies

**DOI:** 10.3897/imafungus.16.144260

**Published:** 2025-07-17

**Authors:** Xiaofei Shi, Shiru Zhang, Gregory M. Mueller, Peigui Liu, Fuqiang Yu, Indunil C. Senanayake

**Affiliations:** 1 Key Laboratory of Chemistry in Ethnic Medicinal Resources, School of Ethnic Medicine, Yunnan Minzu University, Kunming, 650500, Yunnan Province, China; 2 The Germplasm Bank of Wild Species, Yunnan Key Laboratory for Fungal Diversity and Green Development Development & Yunnan International Joint Laboratory of Fungal Sustainable Utilization in South and Southeast Asia, Kunming Institute of Botany, Chinese Academy of Sciences, Kunming, 650201, China; 3 Key Laboratory of Biodiversity and Biogeography, Kunming Institute of Botany, Chinese Academy of Sciences, Kunming 650201, Yunnan Province, China; 4 Program in Plant Biology and Conservation, Northwestern University, 633 Clark Street, Evanston, Illinois 60201, USA; 5 Plant Conservation Science, Chicago Botanical Garden, 1000 Lake Cook Road, Glencoe, Illinois 60022, USA

**Keywords:** Ectomycorrhizal fungi, global diversity, host specificity, phylogeny

## Abstract

*Suillus* Gray (*Suillaceae*, *Boletales*) is an ectomycorrhizal fungal genus with exceptional host specificity associated with *Pinaceae*. The sampling gap in East Asia could be filled by discovering new species and unreported hosts. This study provides a comprehensive multigene dataset (ITS, LSU, *TEFα-1*, *RPB1*, and *RPB2*) of *Suillus*, covering about 80% of known species. Species recognitions by concordance phylogenetic species recognition (GCPSR), concatenation, and coalescent methods were conducted. Seventy-one *Suillus* species are recognized globally using coalescent analyses and GCPSR, of which 12 new species, 5 new locality records, and 14 potentially new species were revealed in East Asia. The higher classification of *Suillus* is another breakthrough supported by morphology and concatenation analyses of protein-coding genes and ribosomal loci. New subgenera *Boletinus*, *Fuscoboletinus* and *Larigni* are all associated with *Larix*, whereas subgenus Douglasia prefers to *Pseudotsugamenziesii* and subgenus Suillus prefers *Pinus*. Subgenus Suillus contains most of the diversity and is further divided into two phylogenetic sections *Suillus* and *Diversipedes*. This study aimed to characterize *Suillus* subgenera, sections, and new species based on molecular data combined with morphology and ecology.

## ﻿Introduction

*Suillus* Gray is a genus of ectomycorrhizal fungi (ECM) in the family *Suillaceae*, suborder *Suillineae*, order *Boletales* of *Basidiomycota* (Binder and Hibbett 2006). This genus is highly specific with *Pinaceae* hosts. That is, each species of *Suillus* is generally associated with single genus, subgenus or even species of *Pinaceae*, mainly with genera *Pinus* L., *Larix* Mill. and *Pseudotsuga* Carrière, but plenty of exceptions have been published. *Suillus* species are ecologically important and vital underground partners for many *Pinaceae* species in the northern hemisphere (Smith and Thiers 1964; Adriaensen et al. 2004, 2005; Paul et al. 2007; Leski et al. 2008; Kwon and Tsuyuzaki 2016; Lofgren et al. 2024). Some *Suillus* species are edible (Smith and Thiers 1964) and some contain compounds with anticancer properties that are used for medical purposes (Leon et al. 2008; Vaz et al. 2012; Santos et al. 2013). Importantly, the “*Suillus—Pinaceae*” relationship is an ideal model for investigating host specificity and host preferring pattern. *Suillus* species are easy to culture *invitro* and therefore facilitates the genomic research (Liao et al. 2016).

Previous generic and subgeneric delimitation of *Suillus* were based on a set of morphological, ecological and molecular characters. Pomerleau and Smith (1962) merged *Boletinus* Kalchbr. into *Suillus* based on morphology. Smith and Thiers (1964) divided *Suillus* into two sections emphasizing smoothness of the cap, stipe annulus and glandular dots. Singer (1986) recognized *Boletinus* Kalchbr. (with clamp connections) as a separate genus from clampless *Suillus* and divided *Suillus* into five sections, two of them associated with *Pinus* and two sections with *Larix* considering glutinous or fibrillose pileus, presence or absence of glandular dots, stipe annulus, and spore print colors as the key features. Singer (1986) also recognized nine subsections within these sections of *Suillus*. Estadès and Lannoy (2001) followed the infrageneric classification of *Suillus* proposed by Singer (1986) with minor changes. *Paragyrodon* (Singer) Singer was removed from the genus *Suillus* by Binder and Bresinsky (2002) due to genetic evidence. Based on Estadès and Lannoy (2001), genus *Suillus* contains viscid or scaly pileus, with or without glandular dots at the stipe apex, and dark staining cystidia when mounted in KOH (Klofac 2013). A detailed history of the proposed subgeneric classifications is presented in Table 1.

**Table 1.** Taxonomic delimitation of infrageneric groups in *Suillus* and genera now included within *Suillus*. The types are in bold.

Reference	Genus and sec. names	Inclusive species	Main features
Pomerleau and Smith 1962	*Fuscoboletinus*	*S.aeruginascens*, *S.glandulosus*, *S.grisellus*, *S.ochraceoroseus*, ***S.sinuspaulianus* (type)**, *S.spectabilis*	pileus viscid, fibrillose or floccose; stipe solid with veils; without glandular dots; spore purple, reddish or vinaceous brown; no clamp connections
Smith and Thiers 1964	Suillussec.Boletinus	*S.appendiculatus*, *S.caerulescens*, *S.castanellus*, *S.cavipes*, *S.decipiens*, *S.floridanus*, *S.grevillei*, *S.imitatus*, *S.lakei*, *S.lithocarpi-sequoiae*, *S.pictus*, *S.ponderosus*, *S.proximus*, *S.pseudobrevipes*, *S.pseudogranulatus*	Membranous annulus, if no annulus, the pileus is fibrillose-squamulose; no glandular dots
	Suillussec.Suillus	*S.acerbus*, *S.acidus*, *S.albidipes*, *S.americanus*, *S.brevipes*, *S.brunnescens*, *S.cothurnatus*, *S.flavoluteus*, *S.fuscotomentosus*, *S.glandulosipes*, *S.granulatus*, *S.hirtellus*, *S.lutescens*, ***S.luteus* (type)**, *S.megaporinus*, *S.pallidiceps*, *S.pinorigidus*, *S.placidus*, *S.punctatipes*, *S.punctipes*, *S.pungens*, *S.ruber*, *S.sibiricus*, *S.subaureus*, *S.subluteus*, *S.subolivaceus*, *S.subvariegatus*, *S.tomentosus*, *S.umbonatus*	with glandular dots, spore print dull cinnamon, gelatinous pileus, no clamp connections
Singer 1986	Boletinussec.Boletinus	***S.cavipes* (type)**, *S.asiaticus*	Clamp present; stipe hollow with veil, glandular dots absent; dry pileus, olive and reddish brown spore print and wide pores
	Boletinussec.Palustres	***S.paluster* (type)**	clamp present; stipe solid, with veil, glandular dots absent; dry pileus, pores extraordinarily wide, spore print purplish brown; occurred in North America
	Suillussec.Solidipedes	*S.amabilis*, *S.decipiens*, *S.ochraceoroseus*, *S.pictus*, *S.spectabilis*	no clamp; pileus fibrillose, with veil; no glandular dots
	Suillussec.Glandulosi	*S.glandulosus*, *S.sinuspaulianus*	pileus viscid, glabrous or fibrillose, spore print purplish, with veils and associate with conifers other than *Larix*
	Suillussec.Larigni	*S.aeruginascens*, *S.bresadolae*, *S.grevillei*, *S.grisellus*, *S.hololeucus*, *S.imitatus*, *S.jacuticus*, *S.nueschii*, *S.proximus*, *S.serotinus*, *S.tridentinus*, possibly *S.ponderosus*	with annulus, without glandular dots; squamose pileus or glabrous, dry or viscid, spore print olive to brownish cinnamon, associate with *Larix*
	Suillussec.Suillus	*S.acerbus*, *S.acidus*, *S.albidipes*, *S.americanus*, *S.bellinii*, *S.brevipes*, *S.brunnescens*, *S.chiapasensis*, *S.collinitus*, *S.cothurnatus*, *S.flavidus*, *S.glandulosipes*, *S.granulatus*, *S.hirtellus*, *S.leptopus*, *S.lutescens*, ***S.luteus* (type)**, *S.pallidiceps*, *S.placidus*, *S.plorans*, *S.pseudobrevipes*, *S.pseudogranulatus*, *S.punctatipes*, *S.punctipes*, *S.pungens*, *S.ruber*, *S.sibiricus*, *S.subalutaceus*, *S.subaureus*, *S.subluteus*, *S.tomentosus*, *S.weaverae*	with glandular dots, with or without veils, spore print color variable, associate with *Pinus*, not with *Larix*
	Suillussec.Fungosi	*S.bovinus*, *S.variegatus*	no glandular dots, no veils, spore print with a slight olive tinge, associate with *Pinus* but not *Larix*
Estadès and Lannoy 2001	Suillussec.Solipedes	***S.spraguei* (type**, synonym of *S.pictus* (Peck) Kuntze)	associate with *Pseudotsuga*, *Pinus*, *Abies*, *Picea*, not with *Larix*
	Suillussec.Larigni	***S.grevillei* (type)**, *S.viscidus*	pileus viscid, glabrous, with veils, without glandular dots
	Suillussec.Suillus	*S.flavidus*, *S.luteus*	not clearly stated
	Suillussec.Granulati	***S.granulatus* (type)**	pileus glabrous, viscid, stipe annulated, with glandular dots
	Suillussec.Fungosi	*S.bovinus*, ***S.variegatus* (type)**	not clearly stated

*Suillus* and its allied taxa in Asia are poorly sampled and infrageneric and many interspecific relationships within the genus *Suillus* may reveal themselves if the Asian collections are studied. An ITS phylogeny on its own does not resolve most deep relationships within *Suillus* (Nguyen et al. 2016). Current rigorous higher classifications of fungi are based on phylogenetic analyses of concatenated multigene datasets (James et al. 2006; Liu et al. 2017). The coalescent analyses are used to resolve phylogenetic and ancestral relationships in lineages with higher incomplete sorting (ILS) level (Wickett et al. 2014; Xi et al. 2014; Zhong et al. 2014; Simmons and Gatesy 2015). Nevertheless, coalescent analysis might provide a different perspective for the higher classification of *Suillus*. The global diversity of *Suillus* species is not well-known. Of the 101 *Suillus* species listed in Index Fungorum (2025) and Mycobank (2025), 58 species from North America and 24 species from Europe are originally described. Eight species are described from the Himalayan region (Gardezi and Sabir 2007; Reddy and Verma 2014, 2015; Verma and Sudhakara 2014; Das et al. 2015; Sarwar et al. 2015), others are from Africa (*S.holomaculatus* Klofac & Hauskn.), Central America (*S.chiapasensis* Singer), East Asia (*S.americanus* (Peck) Snell, *S.cavipoides* (Z.S. Bi & G.Y. Zheng) Q.B. Wang & Y.J. Yao, *S.kunmingensis* (W.F. Chiu) Q.B. Wang & Y.J. Yao), Siberia (*S.americanus* (Peck) Snell), and New Zealand (*S.pungens* Thiers & A.H. Sm.= *S.subacerbus* McNabb; the native range is coastal California and introduced to New Zealand). However, most species were introduced without designating holotype specimens. Many of the *Suillus* collections obtained outside North America and Europe represent cryptic new species that are not yet named and described (Nguyen et al. 2016).

The primary goals of this study are to resolve the phylogenetic relationships within *Suillus* and to address the sampling gap in East Asia with a focus in China. The assessment of phylogenetic relationships was based on ITS, LSU, *TEFα-1*, *RPB1* and *RPB2* data by RAxML and Bayesian analyses. Single gene phylogenies, concatenation and coalescent analyses were compared to determine which method would provide support for higher classification of *Suillus*. Additionally, species recognition methods are compared, including genealogical concordance phylogenetic species recognition (GCPSR), coalescent analysis, concatenation, and barcoding by single gene phylogenies. Detailed taxonomic descriptions of *Suillus* subgenera, sections and new species incorporating morphological, ecological and geographic characters are provided.

## ﻿Materials and methods

### ﻿Specimens

Specimens examined in this study were collected from East Asia, North America and Europe (Table 2). The Mycology Collections Data Portal (MyCoPortal) was searched for species reported from North America, their documented geographic distribution and informing fungarium loan requests. North American herbaria specimens were obtained from Field Museum of Natural History (F); University of California, Berkeley (UC); University of Tennessee Fungarium (Tenn); T.D. Bruns (TDB), and Bradley R. Kropp (Bkr). European *Suillus* specimens were loaned from the Royal Botanic Gardens Fungarium, Kew (KM). East Asian *Suillus* specimens were loaned from Cryptogamic Fungarium, Kunming Institute of Botany (HKAS). Sample collecting was undertaken for over 10 years in China from *Pinus*, *Larix*, and *Pseudotsuga* host plans. Specimens were photographed, georeferenced and deposited in HKAS.

**Table 2.** Sequence accession numbers of *Suillus*. Bold letters indicate sequences originally used in this study.

Taxonomic name	Fungarium number	Collection number	Collection site	Host	ITS	LSU	*TEFα-1*	*RPB1*	*RPB2*
*Suillusacidus*	TENN066904	N/A	USA, Tennessee	*Pinusresinosa*, *P.strobus*	KU721166	KU721522	KU721708	KU852235	KU852347
*S.aenoplacidus*	HKAS91431	RZ082012 04	China, Yunnan	*Pinusarmandii*	KU721269	KU721388	KU721560	KU852238	KU852366
*S.aenoplacidus*	N/A	Aww471	China, Tibet	*Pinusarmandii*	**KU721259**	**KU721380**	**KU721562**	**KU852236**	**KU852365**
*S.aenoplacidus*	HKAS71785	N/A	China, Yunnan	*Pinusarmandii*	**KU721265**	**KU721386**	**KU721566**	N/A	N/A
*S.aestivoluteus*	HKAS63212	Shi690	China, Yunnan	*Pinusyunnanensis*	KU721214	KU721393	KU721602	KU852247	KU852310
*S.aestivoluteus*	HKAS72004	Shi1032	China, Yunnan	*Pinusyunnanensis*	KU721217	**KU721396**	**KU721605**	N/A	N/A
*S.aestivoluteus*	HKAS63183	Shi673	China, Sichuan	*Pinusyunnanensis*	**KU721213**	**KU721394**	**KU721601**	N/A	N/A
*S.alpinus*	HKAS63128	Shi697	China, Yunnan	*Larixpotaninii*	KX342857	KU663237	KU721665	KU852257	KU852328
*S.alpinus*	HKAS57121	feng392	China, Yunnan	*Larixpotaninii*	**KU721284**	**KU663236**	**KU721666**	N/A	**KU852327**
*S.alpinus*	HKAS63232	Shi693	China, Yunnan	*Larixpotaninii*	**KU721283**	**KU663238**	**KU721667**	N/A	N/A
*S.americanus*	N/A	WCG2494	USA, Indiana	*Pinusstrobus*	KU663184	KY489971	KU663205	KU852261	KU852351
*S.americanus*	F1187271	N/A	USA, Indiana	*Pinusstrobus*	KU663181	**KY489972**	**KU663203**	N/A	**KU852354**
*S.americanus*	UC	YNP2355	USA,California	*Pinusstrobus*	**KU663196**	**KY489973**	**KU663204**	N/A	N/A
*S.ampliporus*	HKAS63146	Shi598	China, Heilongjiang	*Larixgmelinii*	KU721541	KU721411	KU721571	KU852274	N/A
*S.ampliporus*	N/A	TDB646	USA, south Michigan	*Larixlaricina*	KU721548	KU721428	KU721572	KU852276	KU852368
*S.asiaticus*	HKAS63145	Shi579	China, Heilongjiang	*Larixgmelinii*	KU721410	KU721248	KU721568	KU852262	KU852369
*S.asiaticus*	F1128638	QXW2408	China, Jilin	*Larixgmelinii*	**KU721247**	**KU721403**	**KU721570**	N/A	**KU852370**
*S.asiaticus*	HKAS63210	Shi596	China, Heilongjiang	*Larixgmelinii*	**KU721250**	**KU721422**	**KU721567**	**KU852263**	N/A
*S.aurihymenius*	HKAS63130	Shi616	China, Neimengu	*Larixgmelinii*	KX342859	KU663242	KU721658	KU852265	KU852326
*S.aurihymenius*	HKAS63129	Shi597	China, Heilongjiang	*Larixgmelinii*	N/A	**KU663241**	**KU721660**	**KU852264**	N/A
*S.aurihymenius*	HKAS63247	Shi607	China, Heilongjiang	*Larixgmelinii*	**KU721285**	**KU663243**	**KU721659**	N/A	N/A
*S.bellinii*	KM143046	N/A	Italy	*Pinuspinaster*, *P.brutia*	KU721183	KU721352	KU721635	KU852266	KU852287
*S.boletoluteus*	HKAS71797	Shi284	China, Yunnan	*Pinusarmandii*	KU721500	KU721375	KU721703	KU852204	KU852346
*S.boletoluteus*	HKAS63244	Shi633	China, Neimengu	Pinussylvestrisvar.mongolica	**KU721499**	**KU721374**	**KU721702**	N/A	N/A
*S.bovinus*	KM164971	N/A	England	*Pinussylvestris*	KU721203	KU721292	KU721733	KU852269	KU852336
*S.bovinus*	HKAS63164	Shi587	China, Heilongjiang	Pinussylvestrisvar.mongolica	KU721196	KU721290	KU721730	N/A	N/A
*S.bovinus*	HKAS63190	Shi637	China, Neimengu	Pinussylvestrisvar.mongolica	**KU721197**	**KU721291**	**KU721728**	N/A	N/A
*S.bresadolae*	HKAS63245	Shi605	China, Heilongjiang	*Larixgmelinii*	KU721449	KU663218	KU721687	N/A	N/A
*S.bresadolae*	HKAS72145	MG346	Italy	*Larixdecidua*	**KU721456**	**KU663225**	**KU721689**	N/A	N/A
*S.brevipes*	UC1860327	N/A	USA, California	*Pinuscontorta*, *P.muricata*	N/A	KU721322	KU721761	N/A	N/A
*S.brevipes*	F1187371	N/A	Canada, Alberta	*Pinuscontorta*	KU721224	KU721323	KU721760	KU852270	KU852302
*S.caerulescens*	HKAS93543	RZ120207	USA, California	*Pseudotsugamenziesii*	KU721188	KU721358	KU721632	KU852272	KU852375
*S.caerulescens*	HKAS93544	RZ120701	USA, California	*Pseudotsugamenziesii*	**KU721190**	**KU721356**	**KU721633**	**KU852273**	**KU852373**
*S.cavipes*	HKAS63161	Shi610	China, Heilongjiang	*Larixgmelinii*	KU721544	KU721415	KU721574	N/A	N/A
*S.cavipes*	HKAS63148	Shi580	China, Heilongjiang	*Larixgmelinii*	**KU721542**	**KU721412**	**KU721573**	N/A	N/A
*S.cavipes*	HKAS63149	Shi593	China, Heilongjiang	*Larixgmelinii*	KU721543	KU721413	KU721575	N/A	N/A
*S.cavipes*	HKAS71862	Shi713	China, Sichuan	*Larixpotaninii*	KU721546	KU721423	KU721576	N/A	N/A
*S.cinerescens*	HKAS63243	Shi664	China, Shanxi	*Pinusarmandii*	KU721159	KU721317	KU721740	KU852278	N/A
*S.cinerescens*	HKAS71893	Shi763	China, Hubei	*Pinusarmandii*	KU721160	KU721318	KU721741	KU852279	N/A
*S.cinerescens*	HKAS57687	wu155	China, Yunnan	*Pinusarmandii*	**KU721162**	**KU721315**	**KU721742**	N/A	N/A
*S.cinerescens*	HKAS63234	Shi558	China, Heilongjiang	*Pinuskoraiensis*	KU721163	KU721316	KU721739	KU852277	N/A
*S.decipiens*	HKAS93525	BKr102900 2	Belize	*Pinuscaribaea*	KU721495	KU721521	KU721709	N/A	KU852362
*S.elbensis*	TENN061628	N/A	Canada	*Larixlaricina*	**KU721465**	**KU663235**	**KU721679**	**KU852217**	**KU852330**
*S.flavidus*	KM171907	N/A	Scotland	*Pinussylvestris*	KU721177	KU721378	KU721707	N/A	N/A
*S.flavidus*	F1187506	N/A	Canada, Alberta	*Pinuscontorta*	**KU721494**	**KU721377**	**KU721705**	N/A	**KU852353**
*S.flavopunctipes*	HKAS91433	RZ082012 07	China, Yunnan	*Pinusyunnanensis*	KX342861	KU721336	KU721624	KU852282	KU852298
*S.flavopunctipes*	HKAS71974	Shi961	China, Guangdong	*Pinusyunnanensis*	KU721239	**KU721335**	**KU721625**	N/A	N/A
*S.flavopunctipes*	HKAS57630	Wu98	China, Yunnan	*Pinusyunnanensis*	**KU721240**	**KU721332**	**KU721622**	N/A	N/A
*S.fluryi*	KM126760	N/A	England	*Pinus sect. Pinus*	KU721180	KU721349	N/A	N/A	KU852293
*S.fuscotomentosus*	C0300855F	BW6	USA, California	*Pinusradiata*, *P.ponderosa*, *P.muricata*	KU721158	KU721309	KU721747	KU852283	KU852340
*S.fuscotomentosus*	N/A	RZ120204	USA, California	*Pinusradiata*, *P.ponderosa*, *P.muricata*	KU721155	**KU721307**	**KU721743**	**KU852284**	**KU852337**
*S.fuscotomentosus*	N/A	TDB3795	USA, California	*Pinusradiata*, *P.ponderosa*, *P.muricata*	KU721153	**KU721310**	**KU721744**	N/A	**KU852338**
*S.glandulosipes*	N/A	TDB–3050	USA, California	*Pinusbanksiana*	KU721225	KU721325	KU721759	KU852286	KU852301
*S.glandulosipes*	N/A	F1104884	USA, Michigan	*Pinusbanksiana*	**KU721226**	**KU721324**	**KU721758**	N/A	N/A
*S.granulatus*	HKAS63139	Shi443	China, Jilin	*Pinusheldreichii*, *P.densiflora*	**KU721246**	**KU721345**	**KU721629**	N/A	N/A
*S.granulatus*	HKAS63166	Shi638	China, Neimengu	*Pinustabuliformis*	**KU721244**	**KU721346**	**KU721628**	N/A	N/A
*S.granulatus*	KM172141	N/A	Italy	N/A	KU721242	KU721344	KU721637	N/A	KU852295
*S.grevillei*	HKAS63206	Shi604	China, Heilongjiang	*Larixgmelinii*	KU721480	KU663265	KU721654	KU852229	KU852320
*S.grevillei*	HKAS56512	Yang5014	Germany	*Larixdecidua*	**KU721469**	**KU663252**	**KU721642**	N/A	**KU852323**
*S.grevillei*	F1186939	N/A	USA, Idaho	*Larixlaricina*	**KU721467**	**KU663250**	**KU721657**	**KU852225**	**KU852322**
*S.grevillei*	HKAS71890	N/A	China, Shanxi	*Larixgmelinii*	**KU721485**	**KU663267**	**KU721643**	N/A	N/A
*S.grevillei*	HKAS63221	Shi508	China, Heilongjiang	*Larixgmelinii*	**KU721482**	**KU663259**	**KU721645**	N/A	N/A
*S.grevillei*	HKAS71880	Shi741	China, Gansu	*Larixgmelinii*	**KU721483**	**KU663254**	**KU721644**	**KU852231**	N/A
*S.grevillei*	TENN061282	N/A	Canada	*Larixlaricina*	**KU721490**	**KU663260**	**KU721652**	N/A	**KU852321**
*S.grevillei*	KM90726	N/A	UK, Wales	*Larixdecidua*	**KU721487**	**KU663268**	**KU721655**	N/A	N/A
*S.grevillei*	N/A	TDB570	USA, south Michigan	*Larixlaricina*	KU721489	KU663262	KU721634	KU852234	KU852378
*S.grevillei*	KM172554	N/A	England	*Larixdecidua*	KU721486	KU663249	KU721653	KU852232	KU852324
*S.grisellus*	N/A	TDB574	USA, south Michigan	*Larixlaricina*	KU721464	KU663234	KU721680	KU852216	KU852329
*S.grisellus*	HKAS63241	Shi627	China, Neimengu	*Larixgmelinii*	KU721448	KU663217	KU721676	KU852209	N/A
*S.hirtellus*	TENN064576	N/A	USA, Tennessee	*Pinusechinata*, *P.taeda*, *P.virginiana*	KU721152	KX171000	KX171004	N/A	KX171007
*S.hirtellus*	TENN065637	N/A	USA, North Carolina	*Pinuscontorta*	N/A	**KU721304**	**KU721752**	**KU852206**	**KU852342**
*S.hirtellus*	TENN065638	N/A	USA, North Carolina	*Pinuscontorta*	N/A	**KU721305**	**KU721753**	**KU852207**	**KU852343**
*S.kwangtungensis*	HKAS71979	Shi978	China, Guangdong	*Pinuskwangtungensis*	KU721518	KU721539	KU721710	KU852239	N/A
*S.kwangtungensis*	HKAS90666	Rui364	China, Guangdong	*Pinuskwangtungensis*	KU721540	KU721519	KU721711	KU852240	N/A
*S.lakei*	N/A	RZ120208	USA, California	*Pseudotsugamenziesii*	KU721189	KU721361	KU721589	KU852242	KU852374
*S.lakei*	KM170241	N/A	Czech	*Pseudotsugamenziesii*	**KU721186**	**KU721360**	**KU721631**	**KU852241**	**KU852377**
*S.longiflavopunctipes*	HKAS71922	Shi828	China, Shandong	*Pinustabuliformis*	KU721235	**KU721340**	N/A	**KU852195**	**KU852296**
*S.longiflavopunctipes*	HKAS63209	Shi445	China, Jilin	*Pinustabuliformis*	KU721233	KU721341	KU721617	KU852194	N/A
*S.longiflavopunctipes*	HKAS71924	Shi830	China, Shandong	*Pinustabuliformis*	**KU721234**	**KU721342**	**KU721619**	N/A	N/A
*S.luteus*	KM171470	N/A	England	*Pinussylvestris*	KU721218	KU721399	N/A	N/A	KU852311
*S.luteus*	N/A	PRL11285	USA	*Pinusresinosa*	KU721219	KU721398	KU721606	KU852248	KU852308
*S.luteus*	HKAS56521	N/A	Germany	*Pinussylvestris*	**KU721210**	N/A	**KU721599**	N/A	N/A
*S.mediterraneensis*	KM169468	N/A	France	*Pinushalepensis*	N/A	KU721353	KU721616	N/A	KU852290
*S.megaporinus*	UC1860326	N/A	USA, California	*Pinuscontorta*	KU721178	KU721379	KU721701	N/A	N/A
*S.minusculus*	HKAS89874	Bo113	China, Guangdong	*Pinuskwangtungensis*	KU721492	KY489974	KU721706	KU852193	KU852352
*S.minusculus*	HKAS71980	Shi979	China, Guangdong	*Pinuskwangtungensis*	KU721491	**KY489975**	N/A	N/A	N/A
*S.minusculus*	HKAS90665	Rui363	China, Guangdong	*Pinuskwangtungensis*	**KU721493**	**KY489976**	N/A	N/A	N/A
*S.ochraceoroseus*	N/A	SAR84- 137	USA, Washington	*Larixlyallii*	**KU721251**	**KU721426**	**KU721585**	N/A	**KU852372**
*S.paluster*	HKAS63135	Shi599	China, Heilongjiang	*Larixgmelinii*	KU721254	KU721408	KU721582	N/A	N/A
*S.paluster*	F1186906	N/A	USA, Idaho	*Larixoccidentalis*	KU721258	KU721404	KU721584	KU852250	N/A
*S.phylolaricinus*	HKAS63176	Shi696	China, Yunnan	*Larixpotaninii*	KU721438	KU663211	KU721693	KU852243	KU852332
*S.phylolaricinus*	HKAS71995	Shi1022	China, Yunnan	*Larixpotaninii*	**KU721439**	N/A	N/A	N/A	N/A
*S.phylolaricinus*	HKAS63132	Shi260	China, Yunnan	*Larixpotaninii*	**KU721437**	**KU663209**	**KU721692**	N/A	N/A
*S.phylopictus*	HKAS91411	RZ072812 02	China, Yunnan	*Pinusarmandii*	KU721529	KU721508	KU721715	KU852254	KU852361
*S.phylopictus*	HKAS90664	Rui362	China, Guangdong	*Pinuskwangtungensis*	KU721538	KU721517	KU721712	KU852253	N/A
*S.phylopictus*	HKAS91424	RZ081112 03	China, Yunnan	*Pinusarmandii*	KU721530	KU721509	KU721719	KU852255	KU852360
*S.phylopictus*	HKAS53409	Li1064	China, Hunan	*Pinusarmandii*	KU721536	KU721515	KU721713	N/A	N/A
*S.phylosubaureus*	HKAS56316	Li1476	China, Yunnan	*Pinusarmandii*	KU721172	KU721367	KU721694	N/A	N/A
*S.phylosubaureus*	HKAS71798	Shi285	China, Yunnan	*Pinusarmandii*	KU721174	KU721369	KU721696	N/A	N/A
*S.phylosubaureus*	HKAS71801	Shi288	China, Yunnan	*Pinusarmandii*	**KU721175**	**KU721370**	**KU721697**	N/A	N/A
*S.pinetorum*	HKAS63200	Shi393	China, Guizhou	*Pinusyunnanensis*	KU721198	KU721295	KU721732	KU852268	N/A
*S.pinetorum*	HKAS63137	Shi344	China, Hunan	*Pinusmassoniana*	KU721195	KU721294	KU721729	KU852267	N/A
*S.pinetorum*	HKAS91427	RZ081512 03	China, Yunnan	*Pinusyunnanensis*	**KU721208**	**KU721300**	**KU721736**	**KU852188**	**KU852334**
*S.pinetorum*	HKAS91407	RZ072312 01	China, Yunnan	*Pinusyunnanensis*	**KU721205**	**KU721297**	**KU721734**	**KU852186**	**KU852333**
*S.placidus*	TENN062310	N/A	USA,Massachusetts	*Pinusstrobus*	**KU721274**	**KU721389**	**KU721558**	**KU852189**	**KU852364**
*S.placidus*	F1112674	N/A	USA, Michigan	*Pinusstrobus*	KU721260	**KU721381**	**KU721559**	N/A	**KU852363**
*S.plorans*	KM104747	N/A	Italy	*Pinuscembra*	KU721164	KU721312	KU721737	N/A	N/A
*S.plorans*	HKAS63225	Shi588	China, Heilongjiang	*Pinuspumila*	KU721165	KU721313	KU721738	N/A	N/A
*S.ponderosus*	N/A	RZ120201	USA, California	*Pseudotsugamenziesii*	KU721187	KU721364	KU721590	KU852190	KU852376
*S.pseudobrevipes*	N/A	YNP2390	USA, California	*Pinusponderosa*	KU721229	KU721329	KU721612	KU852191	KU852305
*S.pseudobrevipes*	N/A	YNP2681	USA, California	*Pinusponderosa*	**KU721230**	**KU721330**	**KU721613**	**KU852192**	**KU852304**
*S.pungens*	N/A	RZ120202	USA, California	*Pinusradiata*	KX342862	KU721326	KU721630	KU852196	KU852299
*S.pungens*	N/A	RZ120203	USA, California	*Pinusradiata*	N/A	**KU721327**	**KU721638**	**KU852197**	**KU852300**
*S.quiescens*	C0300858F	RZ120206	USA, California	*Pinusmuricata*, *Pinusradiata*	KU721184	KU721354	KU721756	KU852198	KU852288
*S.quiescens*	N/A	TDB3048	USA, California	*Pinusmuricata*, *Pinusradiata*	**KU721185**	**KU721355**	**KU721757**	**KU852199**	**KU852289**
*S.salmonicolor*	HKAS93524	BKr1029001	Belize	*Pinuscaribaea*	KX170996	KX170999	KX171003	N/A	KX171006
*S.sinuspaulianus*	N/A	DAOM66995	Canada, Quebec	*Larix*	L54078	KU721430	KU721557	KU852200	KU852317
*Suillus* sp.	HKAS71782	Shi266	China, Yunnan	Pinussect.Pinus	KU721182	KU721350	N/A	N/A	N/A
*Suillus* sp.	N/A	Aww521	China, Yunnan	*Pinusyunnanensis*	KU721181	KU721351	KU721614	N/A	KU852294
*S.spectabilis*	N/A	TDB641	USA, Michigan	*Larixlaricina*	KU721556	KU721429	KU721596	KU852221	KU852313
*S.spectabilis*	HKAS63177	Shi606	China, Heilongjiang	*Larixgmelinii*	**KU721553**	**KU721417**	**KU721594**	**KU852222**	**KU852314**
*S.spectabilis*	HKAS63208	Shi611	China, Heilongjiang	*Larixgmelinii*	**KU721555**	**KU721421**	**KU721595**	**KU852224**	**KU852315**
*S.spraguei*	TENN065429	N/A	USA, Tennessee	*Pinusstrobus*	**KU721524**	**KU721503**	N/A	**KU852202**	**KU852357**
*S.spraguei*	AFTOL717	N/A	USA, Massachusett s	*Pinusstrobus*	AY854069	NG_027637	AY883429	AY858965	AY786066
*S.subaureus*	F1189253	N/A	USA	*Pinusstrobus*	KU721171	KU721365	KU721699	KU852203	KU852356
*S.subaureus*	F1187760	N/A	USA, Indiana	*Pinusstrobus*	KU721170	**KU721366**	**KU721700**	N/A	**KU852355**
*S.subcinnamomeus*	HKAS63222	Shi601	China, Heilongjiang	*Pinuspumila*	KU721496	N/A	N/A	N/A	N/A
*S.subcinnamomeus*	HKAS63240	Shi615	China, Heilongjiang	*Pinuspumila*	KU721497	N/A	N/A	N/A	N/A
*S.subsibiricus*	HKAS91415	RZ072912 02	China, sichuan	*Pinusarmandii*	KU663189	KU721520	KU663197	KU852259	KU852349
*S.subsibiricus*	HKAS91430	RZ082012 03	China, Yunnan	*Pinusarmandii*	**KU663191**	N/A	**KU663201**	**KU852260**	**KU852350**
*S.subsibiricus*	HKAS63155	Shi494	China, Heilongjiang	*Pinuskoraiensis*	KU663190	N/A	**KU663198**	**KU852258**	**KU852348**
*S.tomentosus*	F1186917	N/A	USA, Idaho	*Pinuscontorta*	KU721157	KU721301	KU721748	KU852205	KU852345
*S.tomentosus*	F1187341	N/A	Canada, Alberta	*Pinuscontorta*	**KU721156**	**KU721302**	**KU721749**	N/A	**KU852344**
*S.tomentosus*	KM169463	N/A	England	Pinussect.Pinus	N/A	KU721306	KU721750	N/A	KU852341
*S.tridentinus*	N/A	HB347	West Germany	*Larixdecidua*	KU721289	KU663244	KU721661	KU852208	KU852325
*S.viscidus*	F1186934	N/A	USA, Idaho	*Larix*	**KU721431**	**KU663206**	**KU721678**	**KU852244**	**KU852379**
*S.viscidus*	F1187077	N/A	USA	*Larix*	**KU721432**	**KU663207**	**KU721682**	N/A	N/A
*S.viscidus*	KM166812	N/A	England	*Larixdecidua*	KU721460	KU663230	KU721685	KU852213	KU852331
*S.viscidus*	HKAS71870	Shi726	China, Gansu	*Larixgmelinii*	KU721452	KU663221	KU721686	KU852211	N/A
*S.viscidus*	N/A	Shi764	China, Hubei	*Larix*	**KU721463**	**KU663233**	**KU721669**	**KU852215**	N/A
*S.weaverae*	HKAS94881	RZH104	USA, Wisconsin	Pinussubgen.strobus	KX170995	N/A	N/A	N/A	N/A
*S.zangii*	HKAS63238	Shi702	China, Yunnan	*Pinusarmandii*	KX342860	KU721320	KU721725	KU852219	KU852367
*S.zangii*	HKAS63237	Shi701	China, Yunnan	*Pinusarmandii*	KU721169	**KU721319**	**KU721727**	**KU852218**	N/A
*S.zangii*	HKAS63254	Shi703	China, Yunnan	*Pinusarmandii*	KU721168	**KU721321**	**KU721726**	**KU852220**	N/A

Herbaria and personal collections for specimen deposits are as: Field Museum of Natural History (F); University of California, Berkeley (UC); Cryptogamic Fungarium, Kunming Institute of Botany (HKAS); Royal Botanic Gardens, Kew (KM); University of Tennessee Fungarium (Tenn); T.D. Bruns (TDB) and Bradley R. Kropp (Bkr).

### ﻿Morphological study

Colors were recorded following Kornerup and Wanscher (1961). Singer (1945) and Smith and Thiers (1964) were followed for macro-morphological studies. For basidiospores, measurement was taken from *n* number of basidiospores in *m* number of sporophores (sporocarps) from *p* number of specimens, written as [n/m/p]. Basidiospores measurement was given as (a) b–c (d) from small to large values, > 90% of the values were within b–c where outliers were in (a) and (d). Quotient of basidiospores is as Q = a ± b, in which “a” is the arithmetic mean and “b” is the standard deviation. Basidia were documented in similar format as basidiospores. Data on hyphal width of pileipelllis, stipitipellis and trama were based on > 30 measurements per specimen.

### ﻿DNA extraction, amplification, and sequencing

*Suillus* specimens collected in the field were dried at 40 °C using a mushroom dryer for fungarium specimens. A small piece of the fresh sporophore was preserved in silica gel or 1× CTAB buffer prior to specimen drying for DNA extraction. The Qiagen DNeasy Plant Mini Kit (Qiagen, Valencia, California) was used to extract DNA from preserved specimens according to the manufacturer’s instructions. An improved CTAB (cetyl trimethyl ammonium bromide) extraction method was also utilized to extract DNA when necessary (Doyle and Doyle 1987; Li et al. 2009). Sequences were obtained from nuclear ribosomal internal transcribed spacer ITS1-5.8S-ITS2 with primer pair ITS1F/ITS4 (White et al. 1990; Gardes and Bruns 1993). For some specimens with degraded DNA, amplification was carried out with internal primers ITS-2 and ITS-3 (White et al. 1990). For the nuclear partial 28S large subunit rRNA genes (LSU), primers LR0R and LR5 were used (Vilgalys and Hester 1990). The primers *TEFα-1*-983f and *TEFα-1*-2212r, with additional internal primer *TEFα-1*-1567r were used for amplification of the nuclear protein coding translation elongation factor EF1-alpha (*TEFα-1*) partial gene (Rehner and Buckley 2005), and one newly designed *Suillus* specific internal primer *TEFα-1*-Sintf (5’- TYR CAC AGC ATG MCA TGG TA -3’). For amplification of the nuclear protein coding RNA polymerase II largest subunit (*RPB1*) partial gene, the primers *RPB1*-Af and *RPB1*-Cr (Stiller and Hall 1997; Matheny 2005), with additional internal primer *RPB1*-Int2.2f (Binder et al. 2010) and *RPB1*-Int2.1r were used (Frøslev et al. 2005). For amplification of the nuclear protein coding RNA polymerase II second largest subunit (*RPB2*) partial gene, the primers *RPB2*-6F and *RPB2*-7.1r (Matheny et al. 2007), newly designed suillus-specific internal primers *RPB2*-SintR (5’- CTC CRT CNTCNT CGC GRT AA -3’) and *RPB2*-SintF (5’- CAC GAC CRG CRT CYG TGT AY -3’) were used.

DNA polymerase chain reaction (PCR) amplification was processed using 15 μl system. A 15 μl PCR reaction system was prepared using Promega PCR Mastermix, containing 10–20 ng of genomic DNA, 0.66 mM Primer, 1.5 mM MgCl2, 0.35 U Taq polymerase, and 200 μM of dNTPs. PCR was performed in PTC-100 Programmable Thermal Cyclers (Bio-Rad, Hercules, CA, S.A.). For amplification of nuclear rRNA ITS regions, after an initial denaturing step of 95 °C for 3 min, 40 cycles were performed with denaturing step at 94 °C for 30s, annealing at 55 °C for 30s, and extension at 72 °C for 1 min, followed by a final extension step of 72 °C for 10 min and termination at 4 °C indefinitely. The PCR program for LSU was the same except for using an annealing temperature of 50 °C for 40 cycles. A two-step touchdown PCR program was used for amplifying *TEFα-1*, *RPB1* and *RPB2* genes: an initial denaturing step of 3 min at 95 °C, 20 cycles of touchdown PCR with denaturing step at 94 °C for 1 min, annealing for 1 min with a 0.5 °C/cycle decrement starting at 58 °C, and extension at 72 °C for 1 min, followed by a 20 cycles of regular PCR (94 °C for 1 min, 50 °C for 1 min, 72 °C for 1 min, and a final extension step of 72 °C for 10 min), and termination at 4 °C indefinitely.

PCR products were purified using ethanol precipitation and then prepared with a BigDye terminator 3.1 cycle sequencing kit (Life Technologies, Carlsbad, California, USA). Nucleotide sequences were obtained using an Applied Biosystems 3730 DNA analyzer (Life Technologies). Sequence data retrieved from the sequencer were assembled into consensus alignments using CODONCODE ALIGNER 3.5.7 (CodonCode Corporation, Dedham, MA). The consensus sequences were manually checked to resolve nucleotide double peaks and then compared against GenBank using BLAST to verify taxonomic identification to the generic level.

### ﻿Phylogenetic analyses

Single gene datasets of ITS, LSU, *TEFα-1*, *RPB1* and *RPB2* were initially aligned in MESQUITE 2.75 with manual adjustments (Edgar 2004; Maddison and Maddison 2011). Phylogenetic trees of single gene datasets were inferred from Maximum likelihood (ML) and Bayesian analyses run in the CIPRES web portal (http://www.phylo.org/) (Miller et al. 2010). ML analyses were performed using RAxML 8.0.0 HPC2 on XSEDE with 1000 bootstrap replicates (Stamatakis 2006). Bayesian phylogenetic analysis was performed using MRBAYES 3.2.6 (Ronquist and Huelsenbeck 2003; Ronquist et al. 2012). Number of substitution types (Nst) was set at 6, with 2 runs, 4 chains per run, each run searching for 1M generations sampling every 1000^th^ generation. The first 10% of the sampled Bayesian trees was discarded as the burnin. For convergence diagnosis, the estimated sample size (ESS) was above 200. *Rhizopogonabietis*, *R.ochraceisporus* and *Truncocolumellacitrina* were chosen as outgroups for the ITS phylogenetic analysis (Binder and Hibbett 2006). *Gomphidiusroseus*, *Chroogomphus* sp., *Rhizopogon* sp. and *Truncocolumellacitrina* were used as outgroups for the LSU analysis. *Gomphidiusroseus* and *Rhizopogon* sp. were used as outgroups for the *TEFα-1* and *RPB2* analyses. *Gomphidiusroseus*, *Chroogomphus* sp. and *Rhizopogon* sp. were used as outgroups for the *RPB1* analysis.

Topological incongruence was assessed among the phylogenies of ITS, LSU, *TEFα-1*, *RPB1* and *RPB2* sequences. Before being concatenated, significant topological incongruence was manually evaluated using a reciprocal ≥ 70% ML bootstrap (ML-BP) and ≥ 0.98 Bayesian posterior probabilities (PP) cutoff. Phylogenies of three different concatenated supermatrices were accessed. The first supermatrix dataset was assembled with four loci including LSU, *TEFα-1*, *RPB1* and *RPB2*. Each sample was selected from the ITS phylogeny to represent a unique haplotype. ITS sequences were not included because of its high level of sequence variability and poor resolution for deeper nodes. No major topological conflicts were detected between single gene and combined gene trees. The second supermatrix dataset was concatenated excluding introns of the protein coding genes. *Rhizopogonnigrescens* and *Gomphidiusroseus* were chosen as outgroups for the two supermatrices. The third supermatrix was assembled with ITS sequences and introns of the protein coding genes representing the more comprehensive species diversity. *Gomphidiusroseus*, *Chroogomphus* sp., *Rhizopogon* sp. and *Truncocolumellacitrina* were outgroups for the third supermatrix. Partitioning strategy and molecular models were searched under the greedy algorithm and Bayesian information criterion (BIC) in PARTITIONFINDER 1.1.1 (Lanfear et al. 2012). Exons and introns, codon positions of the protein coding genes, and ribosomal regions were estimated for the partition strategy and molecular models. Partitioning schemes and substitution models were set for the RAxML analyses under GTRGAMMA with 1,000 bootstrap replicates. For the Bayesian analyses, MRBAYES 3.2.6 (Ronquist et al. 2012) was implemented with the partitioned supermatrices and substitution models suggested by PartitionFinder.

Coalescent analysis of *Suillus* was carried out with ASTRAL-II, which estimated the *Suillus* species tree from unrooted gene trees of ITS, LSU, *TEFα-1*, *RPB1* and *RPB2* phylogenies (Mirarab and Warnow 2015). Bootstrap replication was set to 1,000 and other parameters were as default. All alignments and phylogenies of this study were deposited at the TreeBase under study number S21408.

### ﻿Phylogenetic species recognition by different methods

An assessment of the resulting single gene phylogenies, phylogenies of the concatenated supermatrices, and coalescent tree for resolving species was undertaken. Species were recognized in single gene and multigene phylogenies as supported by > 70% ML-BP or > 0.98 PP, and were recognized in the coalescent phylogeny as supported by > 80% bootstrap. Single gene phylogenies of ribosomal ITS, LSU, protein coding *TEFα-1*, *RPB1* and *RPB2* were also compared for efficiency in species barcoding.

## ﻿Results

### ﻿Sampling

Sampling was undertaken intensively in China from 2007 to 2016 covering almost all Provinces. A total of *c.* 1,000 specimens were collected associated with most known East Asian *Pinus* and *Larix* species. East Asian *Pseudotsuga* forests were also searched, but no *Suillus* were found, consistent with ecological studies (Murata et al. 2013; Wen et al. 2014). Fieldwork in North America included Illinois, California, Wisconsin and Louisiana from 2010 to 2014.

### ﻿Molecular datasets of this study

A total of 229 new sequences (49 ITS, 61 LSU, 60 *TEFα-1*, 27 *RPB1*, 32 *RPB2*) were generated in this study and aligned with additional GenBank sequences (Table 2). For the multigene phylogenies, 66 OTUs estimated from the ITS phylogeny were sampled (72% of known *Suillus*OTUs). Total length of the first supermatrix was 3,914 bp with 858 informative sites. The supermatrix with introns of the protein coding genes was partitioned in three by PARTITIONFINDER: (1) LSU and the first and second codon positions of *TEFα-1*, *RPB1* and *RPB2*, with GTR+I+G as the best model; (2) the third codon positions of the three protein coding genes and introns of *TEFα-1* and *RPB2*, with GTR+G as the best model; and (3) introns of *RPB1* with model GTR+I+G. The second supermatrix without introns was 3,138 bp with 626 informative sites and was partitioned as: (1) LSU and the first and second codon positions of *TEFα-1*, *RPB1* and *RPB2*, with GTR+I+G as the best model; (2) the third codon positions of *TEFα-1*, *RPB1* and *RPB2* with GTR+G as the best model. The third concatenated supermatrix was comprised of 4,753 non-ambiguously aligned characters from 144 *Suillus* specimens and 4 outgroup species. Of these, less than 10 collections lack ITS, LSU and *TEFα-1* regions, 65 collections lack *RPB1* sequences, and 64 collections lack *RPB2* region. The supermatrix was partitioned in four by PartitionFinder: (1) LSU and the first and second codon positions of *TEFα-1*, *RPB1* and *RPB2*, with GTR+I+G as the best model; (2) the third codon positions of the three protein coding genes and introns of *TEFα-1* and *RPB2*, with GTR+G as the best model; (3) ITS regions; and (4) introns of *RPB1* with model GTR+I+G.

### ﻿Subgenera and sections of *Suillus*

**Figure 1.**
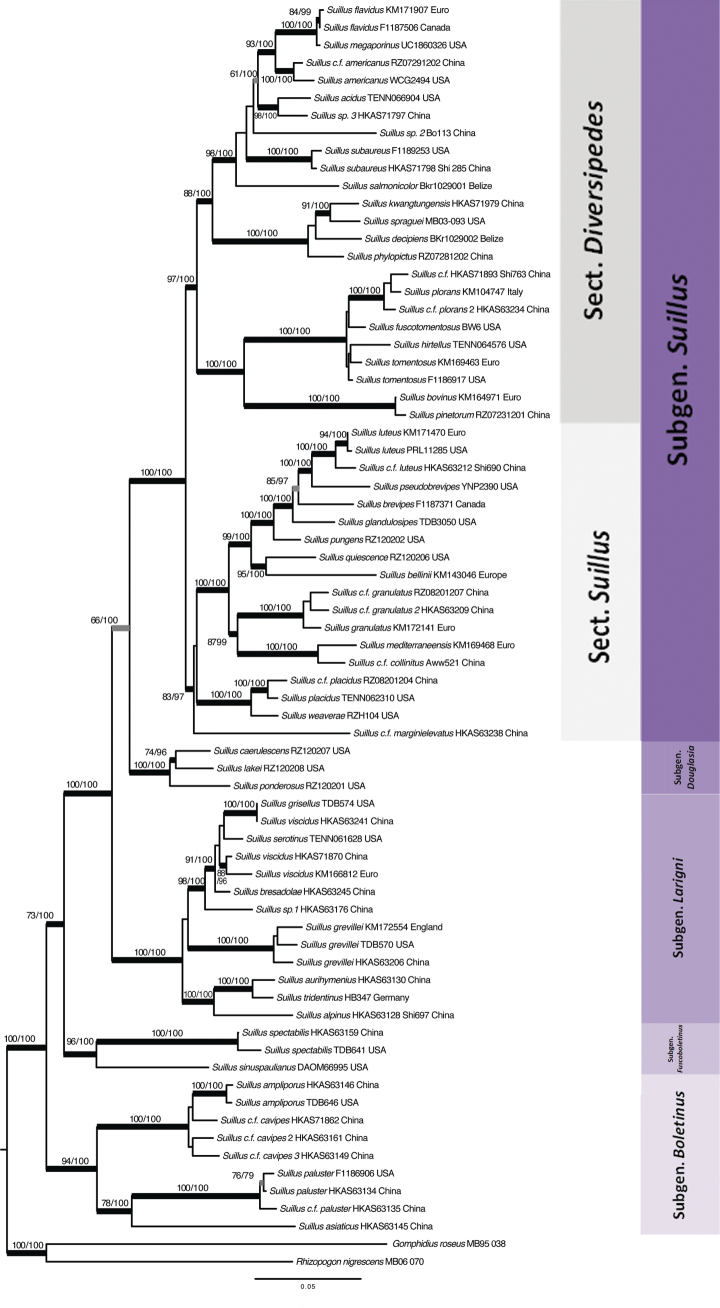
Infrageneric classification of the genus *Suillus* inferred from the phylogeny of concatenated LSU, *TEFα-1*, *RPB1* and *RPB2* sequences with introns. Names of subgenera and sections were annotated by the right side of the figure. Nodes highlighted in thick bars were supported by both ML (≥70%) and PP (≥0.9), annotated above as “ML/PP”. Nodes with thin bars were supported by ML or PP.

**Figure 2.**
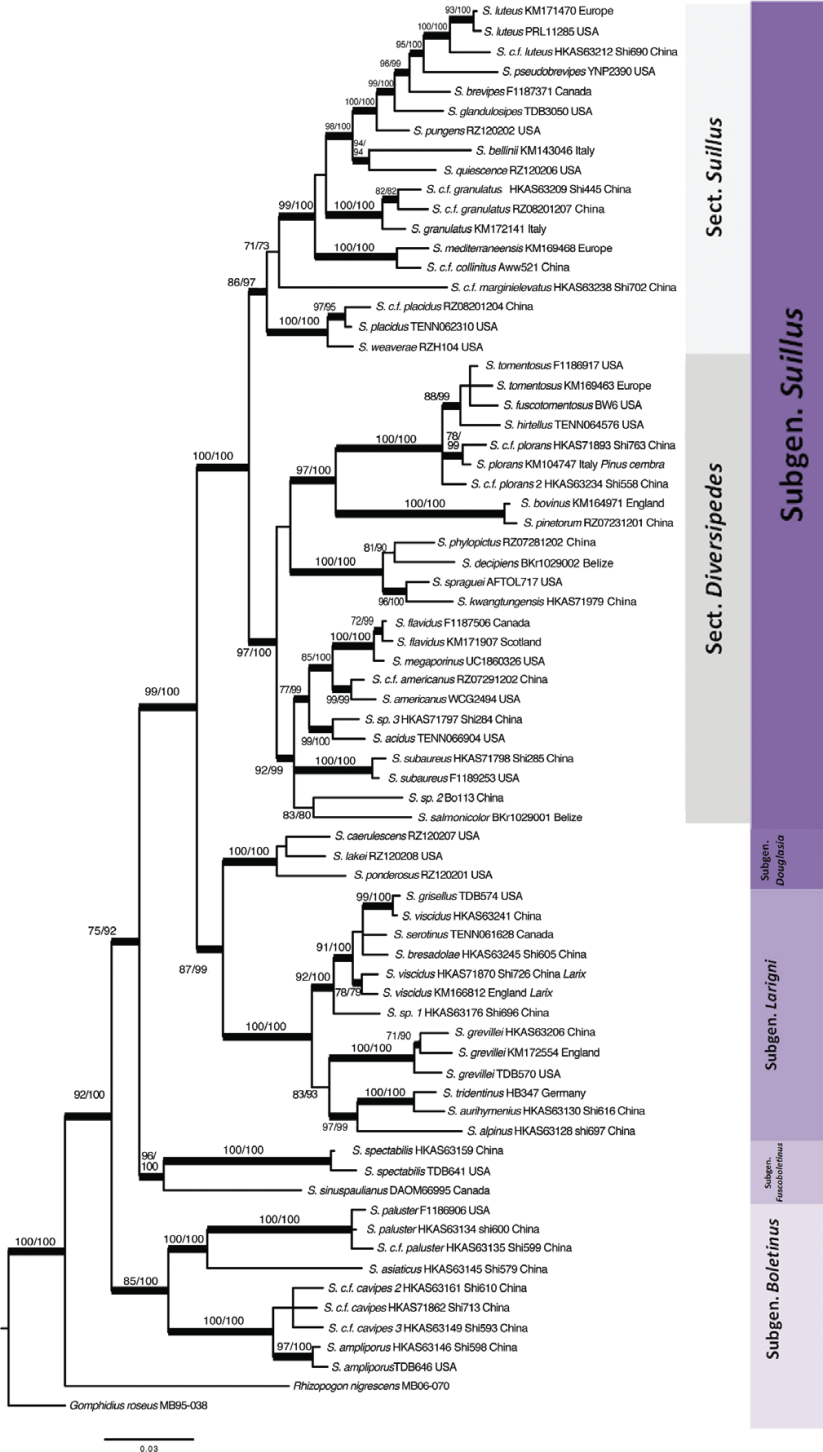
*Suillus* phylogeny of concatenated LSU, and exon regions of *TEFα-1*, *RPB1* and *RPB2*. Names of new subgenera and sections were annotated by the terminal taxa. Nodes highlighted in thick bars were supported by both ML (≥70%) and PP (≥0.9), annotated above as “ML / PP”. Nodes with thin bars were supported by either ML or PP.

Based on the concatenated phylogenies (Figs 1, 2), five subgenera and two sections were recognized in the genus *Suillus* and it reflects the traditional classification in Table 1. Subgenus Boletinus is highly supported as monophyletic clade by the concatenated phylogenies, coalescence and several single gene phylogenies (Table 3). Subgenus Boletinus is sister to a monophyletic clade encompassing all other subgenera. Species in subgenus Boletinus have clamp connections except for *S.ochraceoroseus*, are associated with *Larix*, and have dry caps covered with scales or fibrils. Clamp connections are not found in other subgenera except *Boletinus*. However, all the subsections accepted by Estadès and Lannoy (2001) have not been confirmed by phylogenetic studies in this study.

**Table 3.** Molecular phylogenetic support (RAxML/Bayesian) for accepted subgenera and sections in single and concatenated gene analysis. N/A = not available.

Subgenera and section names	ITS	LSU	*TEFα-1*	*RPB1*	*RPB2*	Coalescence	Concatenation (ITS-LSU-*TEFα-1, RPB1, RPB2*)	Concatenation (LSU-*TEFα-1, RPB1, RPB2*)	Concatenation (*TEFα-1, RPB1, RPB2* without introns)
genus *Suillus*	99/1.00	37/1.00	paraphyletic	99/1.00	50/1.00	0.87	100/1.00	100/1.00	92/1.00
subg. Suillus	N/A	N/A	94/1.00	66/1.00	99/1.00	paraphyletic	100/1.00	100/1.00	100/1.00
sect. Suillus	N/A	N/A	paraphyletic	N/A	84/1.00	paraphyletic	N/A	83/0.97	86/0.98
sect. Diversipedes	N/A	N/A	paraphyletic	N/A	89/1.00	paraphyletic	94/1.00	97/1.00	97/1.00
subg. Boletinus	72/0.89	N/A	84/1.00	96/1.00	39/1.00	0.99	100/1.00	94/1.00	85/1.00
subg. Douglasia	95/1.00	73/1	98/1.00	63/0.99	99/1.00	0.99	100/1.00	100/1.00	100/1.00
subg. Fuscoboletinus	N/A	N/A	89/1.00	paraphyletic	40/0.99	N/A	93/1.00	96/1.00	96/1.00
subg. Larigni	100/1.00	65/1	97/1.00	97/1.00	91/1.00	0.99	100/1.00	100/1.00	100/1.00

SubgenusFuscoboletinus is highly supported as monophyletic by phylogenies of the supermatrices and *TEFα-1* genes (Table 3). Species in this subgenus are associated with *Larix* and have dry or viscid caps with or without scales. Only three species are reported to be in this subgenus, which are distributed in North America and East Asia. Subgenus Larigni is highly supported as monophyletic by the concatenated phylogenies, coalescence and all single gene phylogenies (Table 3). Depending on whether introns of *TEFα-1*, *RPB1* and *RPB2* are included in the phylogenetic analyses, subgenus Larigni is either sister to subg. Douglasia or sister to a monophyletic group composed of subgenera *Douglasia* and *Suillus* (Figs 1, 2). Subgenus Larigni is more speciose than the other two subgenera that have at least some species associated with *Larix*. Subgenus Douglasia is strongly supported as monophyletic by the concatenated phylogenies, coalescence and all single gene phylogenies (Table 3). Subgenus Douglasia is sister to subgenus Larigni or to subgenus Suillus when introns are excluded (Figs 1, 2, Table 3). This subgenus includes only three known species, all associated with *Pseudotsugamenziesii* that is native to the western North America. Species in subgenus Douglasia have dry or viscid caps that are glabrous or covered with scales. Subgenus Suillus is strongly supported as monophyletic by phylogenies of the supermatrices and protein coding genes (Table 3). Species in this subgenus are associated with *Pinus* except for *Suillussubaureus* that can form association with *Quercus*.

SubgenusSuillus contains more species than other subgenera. Morphological features of this subgenus vary over a wide spectrum viz., pilei are dry or viscid, partial veils and glandular dots may or may not be present. All species with glandular dots or caulocystidia are in this subgenus. Subgenus Suillus is further divided into two sections. Both sections are monophyletic, supported by phylogenies of the supermatrices and *RPB2* sequences (Table 3). Both sections are associated with *Pinus* subgenera *Pinus* and *Strobus*. Section Suillus is more uniform in morphology. Species are always glabrous and viscid, and usually present a series of morphological variations at different developmental stages. Glandular dots and partial veils are present or absent. Section Diversipedes contains more species than sect. Suillus and is morphologically more complex. Species caps vary from dry to glutinous, glabrous to scaly, with or without glandular dots as well as partial veils, and the basidiomata are in various colors.

### ﻿*Suillus* species recognition by phylogenetic methods

**Figure 3.**
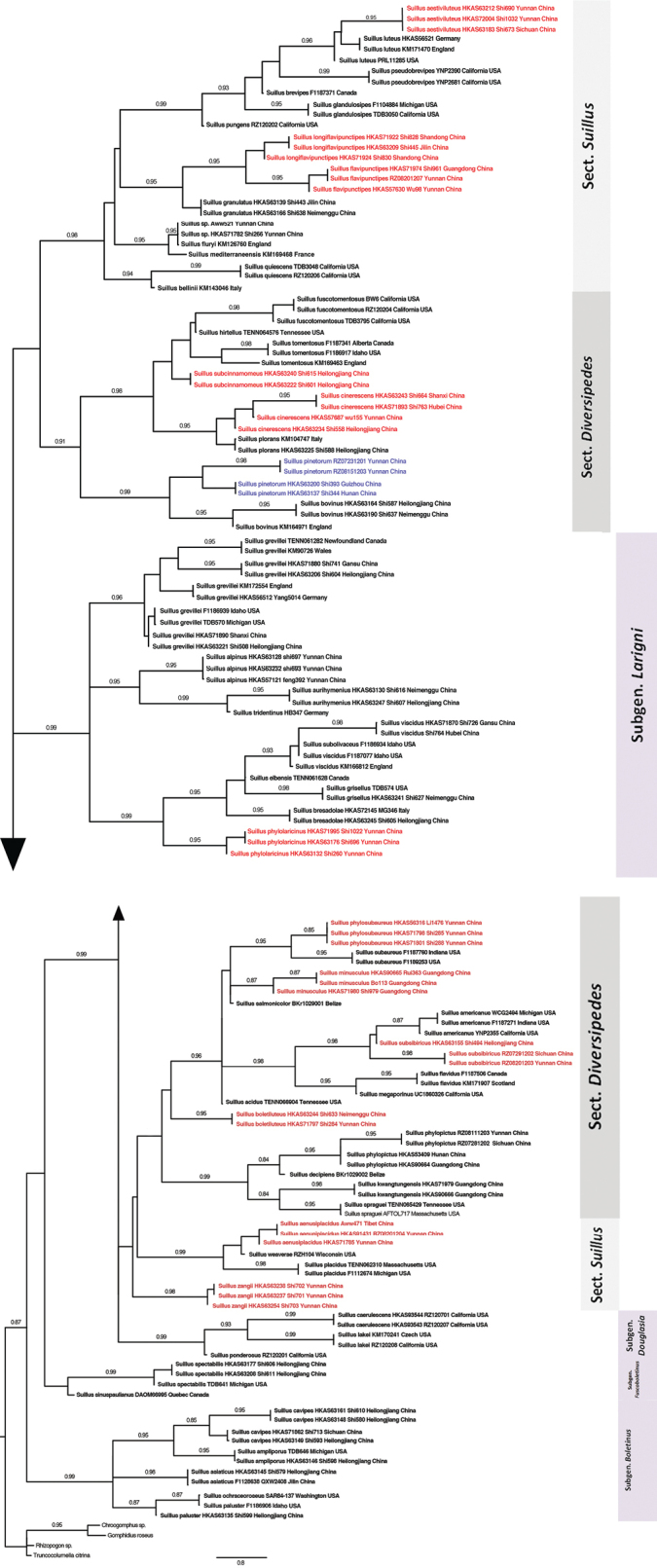
Coalescent phylogram of the genus *Suillus* generated in ASTRAL showing relationships among species. Bootstrap supports over 0.85 are above the branches. Subgenera and sections are annotated close to the terminal taxa. New species are indicated in red and other strains generated in this study are bold. Species names in blue are the redefined *S.pinetorum*.

In this study, GCPSR method was used for species delimitaton in all cases, and the results of GCPSR compared with the results from the other species delimitation methods. A detailed list of phylogenetic species recognized by different methods and an overview of operational taxonomic units (OTUs) supported by each of the methods are in Suppl. material 1: table S1 inferred from figs S1–S6. A total of 71 *Suillus* species are recognized using GCPSR, of which 12 are new species described in this study, 14 are potential new species waiting for adequate morphological and ecological information, and five are new reports from East Asia. The five newly reported species are *Suillusampliporus*, *S.bresadolae*, *S.grisellus*, *S.paluster*, and *S.plorans* all distribute intercontinentally and these species could have a wide distribution in northern latitudes associated with *Larix* sp. Coalescent species recognition is congruent with the GCPSR standard. The output of the coalescent analysis includes a coalescent tree with bootstrap supports for the branches (Suppl. material 1: table S1; Fig. 3). Minor variations in species recognized by coalescence include one additional species in the *S.bovinus* and *S.paluster* complexes, and one species in the *S.fluryi* and *S.grevillei* complexes.

The concatenation analysis tends to identify more species than the other methods (Suppl. material 1: table S1). *Suillusamericanus*, *S.bovinus*, *S.luteus*, *S.paluster* and *S.viscidus* complexes include more species when the concatenation method is applied. For *S.bovinus*, both concatenation and coalescence analyses resolve two cryptic species that are not identified by the GCPSR. Moreover, concatenation differs from all other methods in supporting two cryptic species in the *S.luteus* complex. The ITS barcoding is the most inclusive species recognition method in terms of the number of reference species and multiple representative samples for each species. A total of 92 operation taxonomic units (OTUs) are counted in the ITS phylogeny. Of these, 12 are new species described in this study, 19 are potential new species including three species without proper names from Japan, Russia and Colorado in America, and 16 potential synonyms, including the *S.pseudobrevipes* complex, are not counted as effective OTUs (Suppl. material 1: table S1, fig. S2). The ITS barcoding recognized two OTUs in the *S.ampliporus* complex, whereas GCPSR could confirm only one. A few OTUs supported by extremely short branches are not counted in the *S.acidus*, *S.discolor*, *S.marginielevatus*–*S.indicus* and *S.paluster* complexes (Suppl. material 1: table S1, fig. S2). The ASTRAL species tree was used to delimit OTUs based on the following criteria: (1) branches with > 80% bootstrap support were considered distinct OTUs; (2) for short branches (e.g., < 0.01 substitutions/site), additional evidence from morphology, host association and geographic information was required; (3) singleton collections were excluded from new species designation regardless of branch length, as our sampling protocol requires. This conservative approach prevents over-splitting based on limited evidence. OTU boundaries were assessed phylogenetically, however finalized through consensus analyses with morphological, ecological, and geographic evidence.

Species recognition based on single loci phylogenies of LSU, *TEFα-1*, *RPB1* and *RPB2* are no less effective than the ITS barcoding. Especially for the *Suillusgrevillei* complex, phylogenies of LSU, *TEFα-1* and *RPB2* all outperform the ITS phylogeny in recognizing and supporting cryptic species. Our species delimitation does not rely solely on phylogenetic branch patterns (e.g., bootstrap support or branch lengths) but instead integrates multiple lines of evidence. For example: Morphologically distinct lineages with limited phylogenetic resolution may still be recognized as separate species if supported by diagnostic characters, singletons with long branches are not designated as new species unless corroborated by non-molecular data and well-supported clades may be lumped if no ecological or morphological distinctions exist.

### ﻿Taxonomy

#### ﻿Key to the subgenera of *Suillus*

**Table:** 

1	Clamp connections present in the hyphae of sporophores, stipes hollow, associated with *Larix***subg. Boletinus**
–	Clamp connections absent in the hyphae of sporophores, stipes usually solid, associated with *Larix*, *Pseudotsuga* or *Pinus***2**
2	Associated with *Larix***3**
–	Associated with *Pinus* or *Pseudotsuga*	**4**
3	Pileus usually red, glabrous to scaly, veils glutinous or dry and fibrillose, spore print purplish brown	**subg. Fuscoboletinus**
–	Pileus yellow, orange or brown, glabrous, veils dry and floccose, spore print brownish with olive tinge	**subg. Larigni**
4	Associated with *Pseudotsugamenziesii*, annulated, no glandular dots	**subg. Douglasia**
–	Associated with *Pinus*, with or without veils, with or without glandular dots	**subg. Suillus**

#### ﻿Taxonomic treatments of subgenera, sections and species

##### ﻿ Suillus

Taxon classificationAnimaliaBoletalesSuillaceae

Gray, Nat. Arr. Brit. Pl. (London) 1: 646 (1821)

CD312F3C-8D9D-5A4E-8019-D3347CD36CEB

###### Type.

*Suillusluteus* (L.) Roussel 1796

###### Synonyms:

*Pinuzza* Gray, Nat. Arr. Brit. Pl. (London) 1: 646 (1821). Type: *Pinuzzaflava*Gray. 1821

*Cricunopus* P. Karst., Revue mycol., Toulouse 3(no. 9): 16 (1881). Type: *Cricunopusluteus* (L.) P. Karst. 1881

*Rostkovites* P. Karst., Revue mycol., Toulouse 3(no. 9): 16 (1881). Type: *Rostkovitesgranulatus* (L.) P. Karst. 1881

*Euryporus* Quél., Enchir. fung. (Paris): 163 (1886). Type: *Euryporuscavipes* (Klotzsch) Quél. 1886

*Viscipellis* (Fr.) Quél., Enchir. fung. (Paris): 155 (1886). Type: *Viscipellislutea* (L.) Quél. 1886

*Peplopus* (Quél.) Quél. ex Moug. & Ferry, in Louis, Départ. Vosges, Fl. Vosges, Champ.: 476 [108 repr.] (1887). Type: Viscipellissubgen.Peplopus Quél. 1886

*Ixocomus* Quél., Fl. mycol. France (Paris): 411 (1888). Type: *Ixocomusluteus* (L.) Quél. 1888

*Boletopsis* Henn., in Engler & Prantl, Nat. Pflanzenfam., Teil. I (Leipzig) 1: 194 (1898) [1900]. Type: *Boletopsislutea* (L.) Henn. 1898

*Fuscoboletinus* Pomerl. & A.H. Sm., Brittonia 14: 157 (1962). Type: *Fuscoboletinussinuspaulianus* Pomerl. & A.H. Sm. 1962

*Mariaella* Šutara, Česká Mykol. 41(2): 73 (1987). Type: *Mariaellabovina* (L.) Šutara 1987

*Gastrosuillus* Thiers, Mem. N. Y. bot. Gdn 49: 357 (1989). Type: *Gastrosuillussuilloides* (Thiers) Thiers 1989

##### ﻿ Suillus Gray subg. Suillus

Taxon classificationAnimaliaBoletalesSuillaceae

F61F8436-B534-514E-9BD0-E9CB2E00B0EC

822258

###### Typification.

*Suillusluteus* (L.) Roussel

###### Diagnosis.

This subgenus contains numerous species with a wide spectrum of morphological variations. All are associated with *Pinus* except two species. The clamp connections are lacking.

###### Morphology.

***Basidiomata*** stipitate-pileate with tubular hymenophore. ***Pileus*** develops from hemispherical or convex to subconvex, plane or umbonate, sometimes with wavy margin, viscid to glutinous or dry, glabrous or covered with fibrils or scales. Background color of the cap varies from ivory to yellow. Some species are covered with yellowish, pinkish, red or brown fibrils or scales. Cap color highly variable, colors include ivory, yellowish white, yellow, brown, dark brown, olive brown, olive, pinkish, and red. ***Hymenophore*** adnate, subdecurrent or decurrent, pores 1–2 per mm occasionally up to 5 mm diameter, round to angular, radially arranged or almost lamellate. Some younger ones beaded with white or yellowish droplets. Pores and tubes in some species changing color to brownish or light blue when bruised or cut. Pileus and Stipe ***Context*** white, whitish yellow, yellow or light orange. Stipe context sometimes changes color to blue, greenish blue, reddish or brownish. ***Stipe*** equal to clavate, solid, with or without glandular dots, glandular dots whitish, yellow, reddish, brown, or cinnamon brown when young, becoming cinnamon brown or brown with age. With or without annulus, annulus often superior, white, pinkish or brownish, persistent or evanescent, fibrillose, cottony, gelatinous, glutinous or membranous. ***Mycelia*** often white or pinkish, sometimes changing color to pinkish or red when bruised. ***Spore print*** olive, brown, cinnamon brown or olive brown.

***Basidiospores*** smooth, oblong and inequilateral, hyaline yellow to ochraceous brown in KOH, usually 7–11 μm. ***Basidia*** 4-spored, clavate, hyaline yellow in KOH. ***Cystidia*** abundant, typically in fascicules, large, up to 100 μm, with brown content and surrounded by brown amorphous material in KOH, some species lack caulocystidia. ***Pileipellis*** a layer of gelatinous hyphae, with yellowish hyaline content in KOH; some species with a layer of scales that are smooth and light ochraceous in KOH. ***Clamp connections*** absent.

###### Habitat.

Scattered to gregarious, ectomycorrizal with *Pinus* as primary the host and *Larix* as the secondary host.

###### Known species.

Listed in section Suillus and *Diversipedes*.

###### Notes.

Lofgren et al. (2021) and Zhang et al. (2022) resolved the subgenera within genus Suillus using multigene phylogenies. This subgenus is strongly supported as monophyletic by phylogenies of the supermatrices, *TEFα-1* and *RPB2* genes. This subgenus is the most species rich in genus *Suillus*, containing more species than all other subgenera combined.

Morphological features alone are not very informative for distinguishing species in this subgenus from others. Glandular dots, or caulocystidia visible under the microscope, are exclusive for this subgenus. But not all species in the subgenus have glandular dots. Partial veils may or may not be present. Pilei vary from glabrous to scaly, dry to glutinous and white to a large variety of colors. Micromorphological features are even less effective in delimiting the *Suillus* subgenera. Host association with *Pinus* stands out as the key to differentiate species in this subgenus from those in the other subgenera.

##### ﻿ Suillus Gray sect. Suillus

Taxon classificationAnimaliaBoletalesSuillaceae

80BAB63C-D448-5538-AA7A-B34EA4543F2A

549309

###### Etymology.

autonym.

###### Typification.

*Suillusluteus* (L.) Roussel

###### Morphology.

***Basidiomata*** stipitate-pileate with tubular hymenophore. ***Pileus*** develops from hemispherical to convex or plane, viscid to glutinous, glabrous, with small color streaks that resemble fine fibrils in some species. ***Cap*** is usually covered with a layer of greyish or brown glue when young that dries out or remains viscid. Background color white or yellowish white when young, becoming yellow or dark yellow with age. Cap color varies from whitish yellow to dark brown at maturity. ***Hymenophore*** adnate to subdecurrent, pores 1–2 per mm, round to angular, radially arranged, younger ones are usually beaded with white, yellowish or pinkish droplets. ***Context*** whitish yellow to yellow in pileus and stipe. ***Stipe*** context sometimes changing color to blue, greenish blue, reddish or brownish. Stipe equal to clavate, solid, glandular dots always present, whitish, yellow or reddish when young, becoming cinnamon brown or brown with age. ***Veils*** absent except in the clade of *Suillusluteus* and *S.aestivoluteus*. When present, veil superior, white, cottony or sheer texture. ***Mycelia*** always white or pinkish. ***Spore print*** dull cinnamon, brown, or cinnamon brown.

***Basidiospores*** smooth, oblong and inequilateral, hyaline yellow to ochraceous brown in KOH, usually 7–11 μm. ***Basidia*** 4-spored, clavate, hyaline yellow in KOH. ***Cystidia*** abundant, typically fasciculate, large, up to 100 μm, with brown content and surrounded by brown amorphous material in KOH. ***Pileipellis*** a layer of gelatinous hyphae, with yellowish hyaline content in KOH. ***Clamp connections*** absent.

###### Habitat.

Scattered to gregarious, ectomycorrizal with Pinussubg.Pinus and *Strobus*.

###### Known species.

*Suillusaenoplacidus*, *S.albivelatus*, *S.anomalus*, *S.bellinii*, *S.borealis*, *S.brevipes*, *S.brunnescens*, *S.collinitus*, *S.flavopunctipes*, *S.fluryi*, *S.glandulosipes*, *S.granulatus*, *S.indicus*, *S.kaibabensis*, *S.longiflavopunctipes*, *S.luteus*, *S.aestivoluteus*, *S.marginielevatus*, *S.mediterraneensis*, *S.neoalbidipes*, *S.occidentalis*, *S.placidus*, *S.pseudoalbivelatus*, *S.psuedobrevipes*, *S.pungens*, *S.quiescens*, *S.subalpinus*, *S.triacicularis*, *S.volcanalis*, *S.wasatchicus*, *S.weaverae* and *S.zangii*.

###### Notes.

For this section, pileus is always glabrous, some species have small greyish or brown color streaks resembling fine fibrils. Most species have a glutinous layer over the cap, and the glue appears greyish or brown in younger sporophores. Stipe background color is often white to yellowish white. Glandular dots always present, and usually develop from light color to cinnamon brown or brown with age. Partial veils are absent except in the clade of *Suillusluteus* and *S.aestivoluteus*. These two species have both appendiculate veil remnants on the cap margin and an annulus on stipe. Spore print always dull cinnamon, brown, or cinnamon brown. Cheilocystidia, pleurocystidia and caulocystidia are always abundant. In general, morphological characters alone are not sufficient for delimiting section Suillus from section Diversipedes. Both Pinussubg.Pinus and *Strobus* are hosts for the section.

The current delimitation of the two sections is supported by phylogenies of *RPB2* and concatenated datasets (Table 3. Morphological features are not effective in separating the two sections. In general, section Suillus is morphologically more consistent with the presence of a glutinous pileus and glandular dots. In micromorphology, section Suillus always has a layer of gelatinous hyphae on pileus and a stipe with caulocystidia in fascicles. Host association does not help delimit the two sections because both are associated with *Pinus* subgenera *Pinus* and *Strobus*. Geographic ranges of the two sections overlap.

Estadès and Lannoy (2001) included the type of the genus *Suillus*, *S.luteus* in the Suillussect.Granulati. However, according to the Article 22.2 of the International Code of Nomenclature for algae, fungi, and plants (“A name of a subdivision of a genus that includes the type of the legitimate name of the genus is not validly published unless its epithet repeats the generic name unaltered”), the name of the section is invalid as this section contains the type of the genus *Suillus*. Many synonymous species are found in the *Suilluspseudobrevipes* complex (Nguyen et al. 2016; Suppl. material 1: fig. S2). The ITS phylogeny only supports one species in the *Suilluspseudobrevipes* complex and morphological variations across different developmental stages are found to be common in this complex. This may suggest *S.pseudobrevipes* complex is an actively evolving species complex. Genetic isolation by physical barriers (ex: high mountains, islands) leads to separation and speciation. Therefore, these synonyms may represent multiple species instead of morphotypes in different developmental stages.

##### ﻿ Suillus aenoplacidus

Taxon classificationAnimaliaBoletalesSuillaceae

R. Zhang, X.F. Shi, G.M. Mueller and P.G. Liu sp. nov.

36E11F4B-F275-55F2-8AFC-33EDDE7C9DA5

822262

Figs 4 , 5

###### Etymology.

“*aeno*-” indicates the bronze color of the sticky layer of the cap. “-*placidus*” refers to its sister species *S.placidus*.

**Figure 4.**
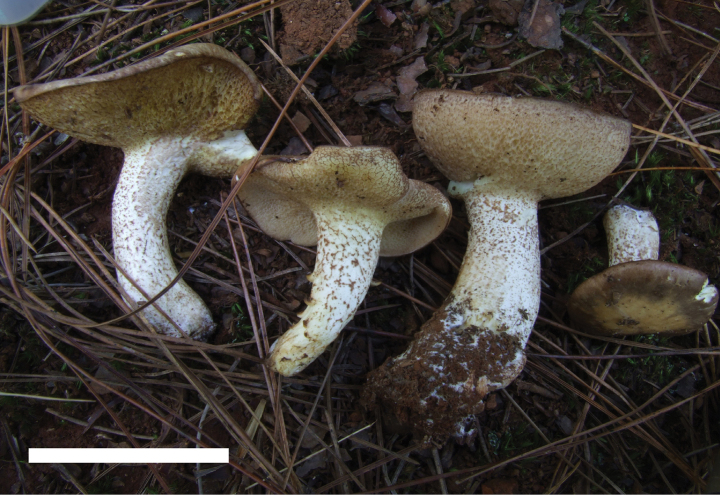
Basidiomata of *Suillusaenoplacidus* (holotype, HKAS 71785). Scale bar: 5 cm.

###### Diagnosis.

*Suillusaenoplacidus* has the bronze glue covering on the cap when young and a white stipe covered with whitish to reddish brown glandular dots. *Suillusaenoplacidus* are associated with two-to-three needle pines.

**Figure 5.**
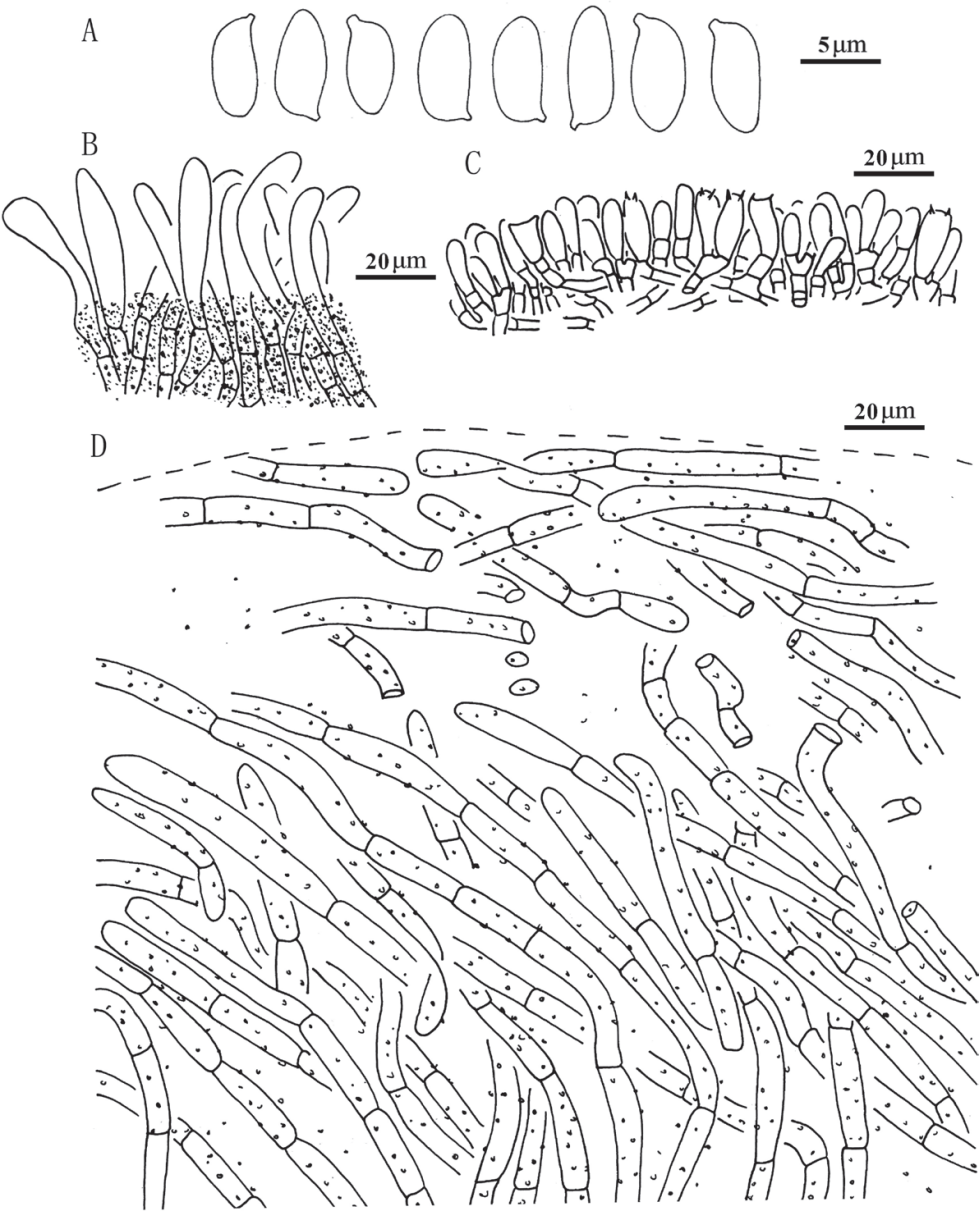
*Suillusaenoplacidus* (holotype, HKAS 71785). **a** Basidiospores; **b** Cheilocystidia; **c** Hymenium with basidia; **d** Pileipellis (mature specimens). Scale bars: 5 μm (**a**); 20 μm (**b–d**).

###### Typification.

**China**: • Yunnan Province, Dali City, Jian Chuan town, Qian Shi Mountain (26°32'17.81"N, 99°53'20.18"E, alt. 2385 m), 07 September 2009, Xiaofei Shi, Shi272 (holotype, HKAS 71785).

###### GenBank.

ITS = KU721265; LSU = KU721386; *TEFα-1* = KU721566

###### Morphology.

***Pileus*** develops from convex to plane, margin incurved when young, wavy at maturity, 2–10 cm diameter, surface viscid to glutinous, glabrous, cap covered with pallid grey (6B2) to bronze brown (5B4, 5B5, 5C5) glue over the white background, cap background color becoming yellow (4A4) at maturity. ***Hymenophore*** adnate to subdecurrent, pale buff (6B2) when young, beaded with pinkish buff (6B3) droplets, turning yellowish (4A5) at maturity. Does not change color when bruised. ***Pores*** 1 to 2 per mm, angular, compound. Pore surface covered with glandular dots. ***Tubes*** shallow, 2–5 mm, whitish yellow (4A2) when young, becoming yellow (4A6) with age. ***Stipe*** 5.0–10.0 × 0.8–1.5 cm, equal to slightly bulbous at base, solid, no veil, stipe cuticle white, covered with glandular dots, dots are whitish when young becoming brownish red (6C6, 6C7, 6D7), big and dense, almost connected to form streaks. ***Mycelia*** white. ***Context*** in pileus white to yellowish, 5–15 mm, stipe context concolorous, unchanging. ***Spore print*** light cinnamon brown (6C2, 6C3). ***Odor and taste*** indistinctive.

***Basidiospores*** [40/2/2] (6.5) 7–8.5 × 3.0–4.0 μm, Q = (2.00) 2.14–2.50, Q_sd_ = 2.31 ± 0.14, smooth, oblong in face view, narrowly inequilateral with a hilar appendage in profile view, hyaline yellow or brown in KOH, tawny ochraceous in Melzer’s. ***Basidia*** 4-spored, clavate, bulbous, 15.0–28.0 × 5.5–8.0 μm, brown in KOH, tawny ochraceous in Melzer’s regent. ***Hymenophoral trama*** divergent, wrinkled, thin-walled, hyaline and mostly 2–7 μm, up to 11 μm. ***Pleuro- and cheilocystidia*** in fascicles, abundant, clavate, up to 100 μm long, content brown or hyaline, surrounded by brown amorphous material in KOH. ***Pileipellis*** densely encrusted by ochraceous granules, hyphae densely distributed as if interwoven, mostly 3–10 μm, up to 15 μm wide. ***Stipitipellis*** covered by patches of brown amorphous pigments, composed of interwoven hyphae about 3–15 μm, up to 23 μm wide. ***Caulocystidia*** abundant, covering the stipe, morphologically similar with pleuro- and cheilocystidia, basal part surrounded by brown pigments, up to 100 μm. ***Context trama*** hyaline, smooth, thin-walled, interwoven, similar in pileus and stipe, mostly 3–30 μm, up to 40 μm wide at the stipe base. ***Clamp connections*** absent.

###### Habitat.

Solitary to scattered, in association with *Pinusarmandii* and *Pinuskoraiensis*.

###### Known distribution.

Currently known from southwestern and northeastern China and Japan.

###### Specimens examined.

**China**: • Yunnan Province, Dali City, Miaopu hill (25°34'7.32"N, 100°12'20.87"E, alt. 2208 m), 20 August 2012, Rui Zhang RZ08201204 (HKAS 91431); **China**: • Tibetan Autonomous Prefecture, Deqin town, valley of Bai Ma Snow Mountain (28°29'42.05"N, 99°02'19.67"E, alt. 4198 m), 12 October 2011, Xiaofei Shi, Shi1026 (HKAS 71999); **China**: • Guizhou Province, Bijie, Hezhang town, Shui Tang plantation, 18 September 2008, Xiaofei Shi, Shi193 (HKAS 71752); **China**: • Guizhou Province, Bijie, *Rhododendron* forest in Bai Mu, 10 August 2009, Xiaofei Shi, Shi195 (HKAS 63319); **China**: • Heilongjiang Province, Yichun City, Wuying National Forest Park (48°14'08.64"N, 129°12'39.22"E, alt. 350 m), 17 August 2010, Xiaofei Shi, Shi555 (HKAS 63151); **China**: • Tibetan Autonomous Prefecture, Lin Zhi, 25 km away from the military base (29°45.566"N, 94°44.050"E, alt. 3150 m), 26 June 2009, A.W. Wilson Aww471.

###### Notes.

This species is morphologically similar to North American *Suillusweaverae* and *S.placidus*. Geographical ranges and hosts are the keys to distinguish these three species. This species has often been morphologically confused with the *Suillusgranulatus* complex. But *Suillusaenoplacidus* is distinguished by association with five-needle pines (Pinussubg.Strobus), whereas *S.flavopunctipes*, *S.granulatus*, and *S.longiflavopunctipes* are associated with two-to-three needle pines (Pinussubg.Pinus). In addition, even though morphological features of the caps are similar, stipe features are very distinct. *Suillusaenoplacidus* has a white stipe covered with whitish to reddish brown glandular dots; *S.flavopunctipes* and *S.longiflavopunctipes* have yellowish stipes, especially close to apex, and their glandular dots develop from yellow to brown. A potential new species, sister to *S.aenoplacidus*, from Japan has been identified in the ITS phylogeny (Suppl. material 1: fig. S2).

##### ﻿ Suillus flavopunctipes

Taxon classificationAnimaliaBoletalesSuillaceae

R. Zhang, X.F. Shi, G.M. Mueller & P.G. Liu sp. nov.

E9D9419D-4226-5877-8C91-FF91BF6B6D26

822263

Figs 6 , 7

###### Etymology.

The epithet refers to the yellow (flavo-) glandular dots (-punctipes) found on the stipe in young sporophores.

**Figure 6.**
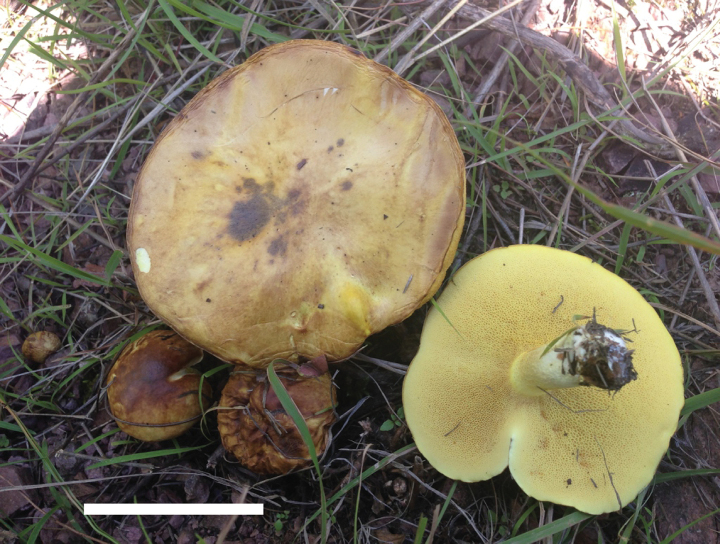
Basidiomata of *Suillusflavopunctipes* (holotype, HKAS 91433). Scale bar: 5 cm.

###### Diagnosis.

*Suillusflavopunctipes* is in the *Suillusgranulatus* morphological complex. This species can easily be differentiated from *S.granulatus* and *S.longiflavopunctipes* by its host association with *Pinusyunnanensis* and *P.massoniana* in southern China.

**Figure 7.**
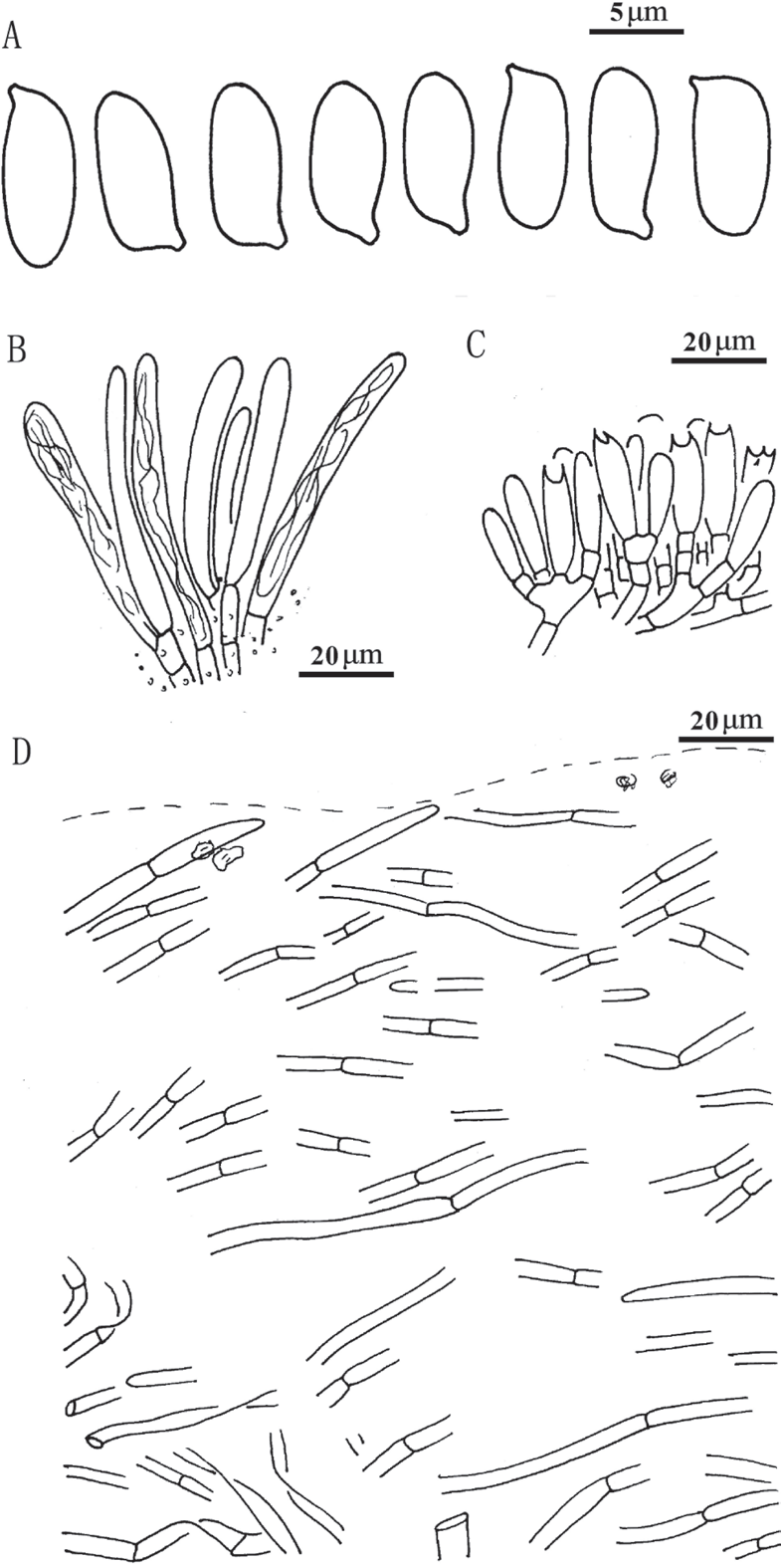
*Suillusflavopunctipes* (holotype, HKAS 91433). **a** Basidiospores; **b**. Cheilocystidia; **c**. Hymenium with basidia; **d**. Pileipellis (mature specimens). Scale bars: 5 μm (**a**); 20 μm (**b–d**).

###### Typification.

**China**: • Yunnan Province, Dali City, Miao-pu hill (25°34'07.32"N, 100°12'20.87"E, alt. 2208 m), 20 August 2012, Rui Zhang RZ08201207 (holotype HKAS 91433).

###### Morphology.

***Pileus*** develops from hemispherical to convex or broadly convex, 1–10 cm diameter, surface viscid to glutinous in young or when moist, glabrous, all white or with a brown glutinous layer when young, developing brown (6D5 to 6F7) patches or streaks above the yellow background (4A5, 4A6) due to deposition of basidiospores from overlaying sporophores which are captured by the glutinous layer which becomes dried out. ***Hymenophore*** adnate to subdecurrent, yellow (3A5), surface covered with yellowish milky droplets when young, dotted with brownish dots in age. Does not change color when bruised. Tube mouths small, 1 to 3 per mm, angular. ***Tubes*** 0.5–5 mm deep, concolorous with pore surface. ***Stipe*** 1.5–6 × 0.5–2.0 cm, cylindrical or tapering downwards, solid, lacking annulus, background color pallid when young, becoming yellowish in apex. Covered with connected glandular dots that form streaks, glandular dots change from yellow (3A4) to cinnamon brown (6E5–6E7) at maturity. ***Context*** yellowish white to yellow (3A3, 3A4) in both pileus and stipe. Does not change color when exposed. ***Spore print*** dark cinnamon brown (6F5–6F7), usually in mass. ***Odor and taste*** pleasant smell, taste indistinctive.

***Basidiospores*** [40/2/2] 8.0–9.5 × 3.0–4.0 μm, Q = (2.00) 2.29–2.83 (3.17), Q_sd_ = 2.49 ± 0.24, smooth, oblong in face view, narrowly inequilateral with a hilar appendage in profile view, hyaline yellow or brown in KOH, tawny ochraceous in Melzer’s. ***Basidia*** 4-spored, clavate, bulbous, 15.0–20.0 × 5.0–6.0 μm, hyaline yellow in KOH, tawny ochraceous in Melzer’s. ***Hymenophoral trama*** divergent, wrinkled, thin-walled, hyaline and 5–11(-16) μm. ***Pleuro- and cheilocystidia*** in fascicles, abundant, clavate, up to 70 μm long, contents brown or hyaline, surrounded by brown amorphous material in KOH. ***Pileipellis*** encrusted by tiny hyaline or ochraceous granules, hyphae densely distributed as if interwoven in yellowish glue in KOH, 3–7(-10) μm. ***Stipitipellis*** covered by patches of brown amorphous pigment, composed of interwoven hyphae about 3–9 μm wide. ***Caulocystidia*** abundant over the stipe, morphologically similar with pleuro- and cheilocystidia, base surrounded by brown pigment, up to 100 μm. ***Context trama*** hyaline, smooth, thin-walled, interwoven, similar in pileus and stipe, mostly 3–30 μm, up to 40 μm wide at stipe base. ***Clamp connections*** absent.

###### Habitat.

Gregarious, in association with *Pinusyunnanensis* and *P.massoniana*.

###### Known distribution.

Mainly distributed in southern China, extending north to Henan Province along with *P.massoniana*.

###### Specimens examined.

**China**: • Yunnan Province, Baoshan City, Long-yang, Banqiao town, Qingshui village (alt. 1800 m), 20 August 2009, Gang Wu, GangWu 98 (HKAS 57630); **China**: • Guangdong Province, Lecang Xian, Jiufeng town, Shi Er Du Shui conservative region, 15 September 2011, Xiaofei Shi, Shi961 (HKAS 71974); **China**: • Henan Province, Nanyang City, Nei Xiang County, Qili Ping pine forest (alt. 720 m), 7 August 2010, Xiaofei Shi, Shi440 (HKAS 63242); **China**: • Hubei Province, Shiyan City, Fang Xian, Xi Song town, Xi Ping village (alt. 1200 m), 31 July 2011, Xiaofei Shi, Shi762 (HKAS 71892); **China**: • Fujian Province, Wuyi Shan City, Long Jing Mountain (alt. 300 m), 4 September 2011, Xiaofei Shi, Shi883 (HKAS 71943).

###### GenBank.

ITS = KX342861; LSU = KU721336; *TEFα-1* = KU721624; *RPB1* = KU852282; *RPB2* = KU852298.

###### Notes.

See above for comparisons of this species with *Suillusaenoplacidus*. Within the *Suillusgranulatus* complex, this species can easily be differentiated from *S.granulatus* and *S.longiflavopunctipes* by its host association and geographic range. *Suillusflavopunctipes* is a southern China species associated with *Pinusyunnanensis* and *P.massoniana*. Morphological characters, including color variations and stipe length, are not stable across large collections and can be affected by growing conditions. For molecular studies, the ITS phylogeny alone can resolve the three species in the *Suillusgranulatus* complex with high support.

##### ﻿ Suillus longiflavopunctipes

Taxon classificationAnimaliaBoletalesSuillaceae

R. Zhang, X.F. Shi, G.M. Mueller and P.G. Liu sp. nov.

C55D8A0B-D5FB-5B0A-B2FB-69A8E27124F3

822264

Figs 8 , 9

###### Etymology.

“*longi*-” means that the stipe of young sporophore is comparatively longer than that of *S.flavopunctipes*.

**Figure 8.**
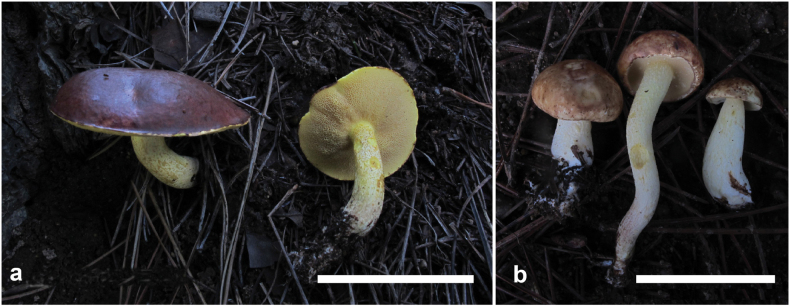
Basidiomata of *Suilluslongiflavopunctipes* (holotype, HKAS 71922). **a** Mature basidiomata. **b** Young basidiomata. Scale bar: 5 cm.

###### Diagnosis.

*Suilluslongiflavopunctipes* young sporocarps have comparatively longer stipes than other similar species in the *S.granulatus* morphological complex. This species is associated with *Pinusdensiflora* and *P.thunbergii*, and one collection is with P.sylvestrisvar.sylvestriformis.

**Figure 9.**
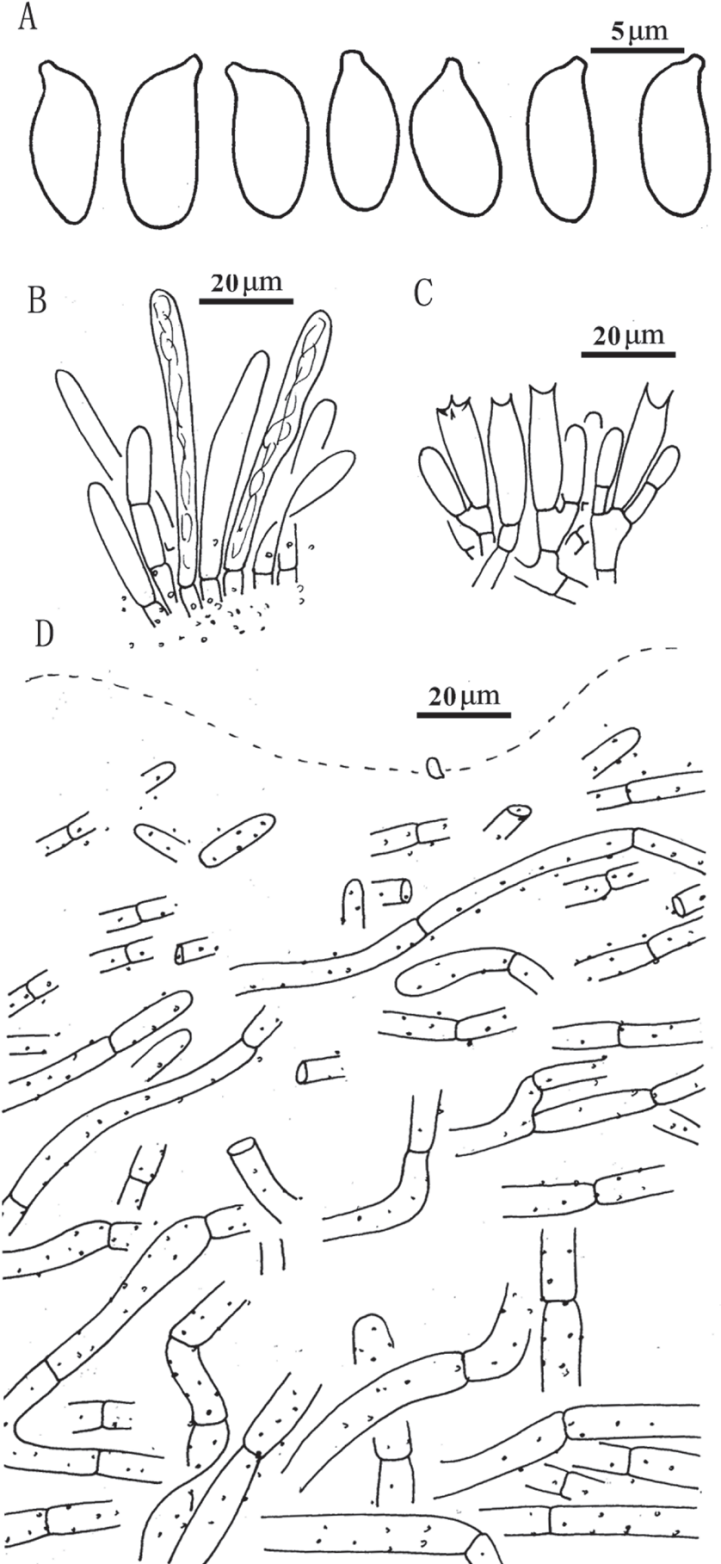
*Suilluslongiflavopunctipes* (holotype, HKAS 71922). **a** Basidiospores; **b** Cheilocystidia; **c** Hymenium with basidia; **d** Pileipellis (mature specimens). Scale bars: 5 μm (**a**); 20 μm (**b–d**).

###### Typification.

**China**: • Shandong Province, Linyi City, Pingyi town, Dianzi Plantation (35°32'22.14"N, 117°55'12.42"E, alt. 250 m), 24 August 2011, Xiaofei Shi, Shi828 (holotype, HKAS 71922).

###### GenBank.

ITS = KU721235; LSU = KU721340; *RPB1* = KU852195; *RPB2* = KU852296.

###### Morphology.

***Pileus*** develops from hemispherical to convex or broadly convex, 2–9 cm diameter, surface viscid to glutinous, glabrous, younger ones all white, or the glutinous layer in cinnamon brown color patches (5B4, 5C5). Color reddish brown without color patches in age (6D5–6F5). ***Hymenophore*** adnate to subdecurrent, yellow (3A5), with milky white droplets when young, dotted with brownish dots when age. Do not change color when bruised. ***Pores*** 1 to 2 per mm, smaller towards margin, angular. ***Tubes*** 2–3 mm deep, concolorous with pore surface. ***Stipe*** 5–7 × 0.8–1.5 cm, cylindrical or tapering downwards, solid, no annulus, background color pallid in young, become yellowish, darker yellow in apex when age. Covered all over with big and almost linked glandular dots, glandular dots change from yellow (3A4) to vinaceous brown (5C4–5E4) when age. ***Context*** yellowish white to yellow (3A3, 3A4) in pileus and stipe. Do not change color when exposed. ***Spore print*** cinnamon brown (5B4, 5C5). ***Odor and taste*** pleasant smell, taste indistinctive.

***Basidiospores*** [80/2/2] (8.0) 8.5–10.0 × 3.5–4.0 μm, Q = 2.25–2.57 (2.71), Q_sd_ = 2.38 ± 0.12, smooth, oblong in face view, narrowly inequilateral with a hilar appendage in profile view, hyaline yellow or brown in KOH, tawny ochraceous in Melzer’s. Basidia 4-spored, clavate, bulbous at top, 22.0–27.0 × 6.0–7.0 μm, yellowish in KOH, tawny ochraceous in Melzer’s. ***Hymenophoral trama*** divergent, wrinkled, thin-walled, hyaline and mostly 5–11 μm, up to 16 μm. *Pleuro*- and *cheilo****cystidia*** in fascicles, abundant, clavate, up to 70 μm long, content brown or hyaline, surrounded by brown amorphous materials in KOH. ***Caulocystidia*** abundant all over the stipe, morphologically similar with pleuro- and cheilocystidia, basal surrounded by brown pigments, up to 100 μm. ***Pileipellis*** encrusted by tiny hyaline or ochraceous granules, hyphae densely distributed as if interwoven in yellowish glue in KOH, most 3–7 μm, up to 10 μm wide. ***Stipitipellis*** covered by profuse brown amorphous pigments, composed of interwoven hyphae, about 3–9 μm wide, most contain brown pigments. ***Context trama*** hyaline, smooth, thin-walled, interwoven, similar in pileus and stipe, mostly 3–30 μm, up to 40 μm wide at stipe base. ***Clamp connections*** absent.

###### Habitat.

Solitary to scattered, in association with *Pinusdensiflora* and *P.thunbergii*, and one collection is with P.sylvestrisvar.sylvestriformis.

###### Known distribution.

Natural range is in northeastern China, Korea and Japan.

###### Specimens examined.

**China**: • Jilin Province, Antu Town, Changbai Mountain, Junshi Bu yard (alt. 748 m), 7 August 2010, Xiaofei Shi, Shi445 (HKAS 63209); **China**: • Shandong Province, Qingdao City, Lao Mountain (alt. 300 m), 18 August 2011, Xiaofei Shi, Shi812; **China**: • Shandong Province, Linqu town, Jiushan village, Black pine forest park (alt. 200 m), 23 August 2011, Xiaofei Shi, Shi824 (HKAS 71918); **China**: • Shandong Province, Linyi City, Pingyi town, Dianzi plantation (alt. 450 m), 24 August 2011, Xiaofei Shi, Shi829 (HKAS 71923); **China**: • Shandong Province, Linyi City, Pingyi town, Dianzi plantation (alt. 450 m), 24 August 2011, Xiaofei Shi, Shi830 (HKAS 71924); **China**: • Jiansu Province, Lianyungang City, Huaguo Mountain (alt. 100 m), 27 August 2011, Xiaofei Shi, Shi837 (HKAS 71925); **China**: • Jiangsu Province, Lianyungang City, Huaguo mountain, 27 August 2011, Xiaofei Shi, Shi838 (HKAS 71926).

###### Notes.

Differences of this species with *Suillusaenoplacidus* and *S.flavopunctipes* are in above commentaries. But it remains confusing for the delimitations of *Suilluslongiflavopunctipes* and *S.granulatus*, the latter distributes from Europe to northeastern China. The two species have overlapped geographic ranges and one shared host P.sylvestrisvar.sylvestriformis. Though *S.granulatussensu stricto* is not found growing with *Pinusdensiflora* and *P.thunbergii*, further studies are required to confirm host associations of the two species. Morphological identifications are not easy to apply for the *S.granulatus* complex, but molecular phylogenies, even ITS phylogeny alone, can separate these species.

##### ﻿ Suillus aestivoluteus

Taxon classificationAnimaliaBoletalesSuillaceae

R. Zhang, X.F. Shi, G.M. Mueller & P.G. Liu sp. nov.

894A8233-D723-5706-AB7A-2FD32041894C

822265

Figs 10 , 11

###### Etymology.

“aestivo-” means summer. This species has very thin and sheer partial veil as a summer version of the European *S.luteus*, which has thick and wooly partial veils.

**Figure 10.**
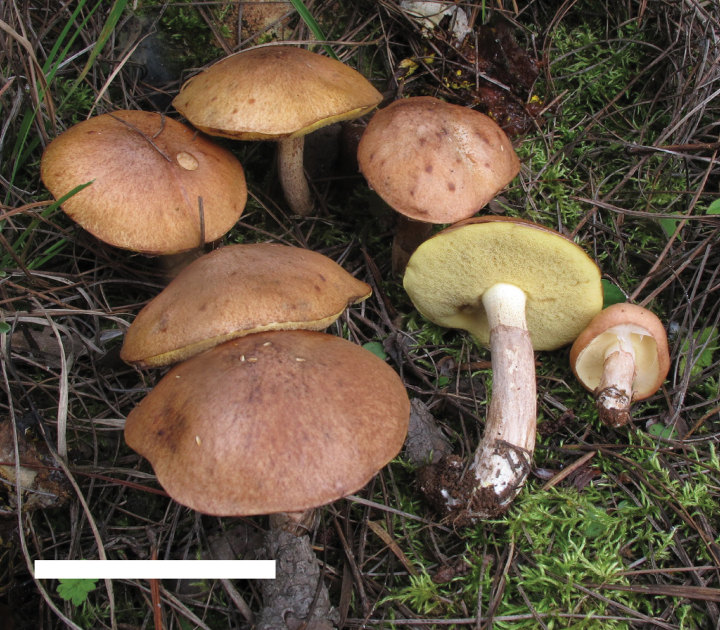
Basidiomata of *Suillusaestivoluteus* (holotype, HKAS 63212). Scale bar: 5 cm.

###### Diagnosis.

Morphologically similar to *S.luteus* but differentiated from it by having thick, persistent and woolly textured annlus or ring, partial veil underneath the ring covers the basal part of the stipe. Glandular dots on lower part of the stipe are covered by the partial veil and could not be seen.

**Figure 11.**
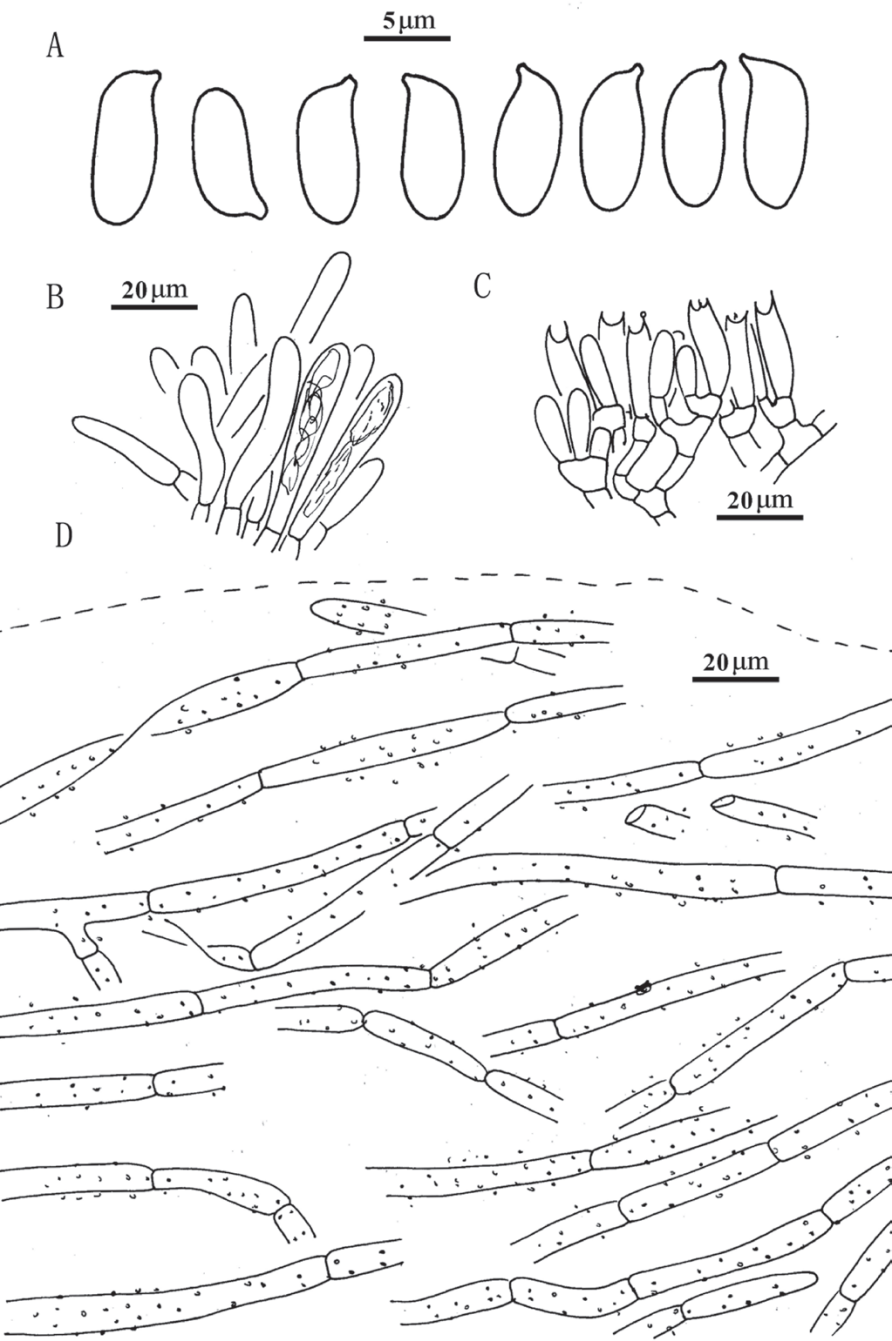
*Suillusaestivoluteus* (holotype, HKAS 63212). **a** Basidiospores; **b** Cheilocystidia; **c** Hymenium with basidia; **d** Pileipellis (mature specimens). Scale bars: 5 μm (**a**); 20 μm (**b–d**).

###### Typification.

**China**: • Yunnan Province, Lijiang City, Yulong Snow Mountain, Gan Hai Zi (26°46'28.57"N, 100°14'09.55"E, alt. 2611 m), 16 September 2010, Xiaofei Shi, Shi690 (holotype, HKAS 63212).

###### Morphology.

***Pileus*** develops from obtuse to convex or flat, 3–12 cm diameter, surface viscid to glutinous, glabrous, background color pallid yellow (4A2) to buff brown (5C4), with brownish or cinnamon brown (5C5, 5D5, 5E7) color streaks, not fibrils, underneath the glue. Color streaks are denser and darker in cap center. Pileus cuticle easily separable. Appendiculated with white and membranous partial veils that fall off with age. ***Hymenophore*** adnate to subdecurrent, younger ones pallid yellow (3A3), turn to dark yellow (4B4) with age. Younger ones covered all over by white and membranous partial veils. Do not change color when bruised. ***Pores*** 2–3 per mm, small and round. ***Tubes*** 3–7 mm deep, concolorous with pore surface. ***Stipe*** 2.0–8 × 0.6–2.0 cm, equal to slightly bulbous in base, white or with yellow tinge in apex, solid, glandular dots all over the stipe, color dark brown (5F7), become smaller and less towards the base. Annulated with white and membranous ring. ***Stipe*** below the annulus covered with sheer remnants of the partial veil, beneath which glandular dots are visible. Annuli turn to buff (5B2) or even brownish (5C3, 5D5), often disappear when old. ***Mycelia*** white. ***Context*** white to yellowish, 6–15 mm deep in pileus. Stipe context concolorous. Do not change color when exposed. ***Spore print*** cinnamon brown (5E7, 5F7). ***Odor and taste*** indistinct.

***Basidiospores*** [80/2/2] 7.0–9.0 (9.5) × 3.0–3.5 (4) μm, Q = (2.00) 2.14–2.71 (2.83), Q_sd_ = 2.43 ± 0.21, smooth, oblong in face view, narrowly inequilateral with a hilar appendage in profile view, hyaline yellow or brown in KOH, tawny yellow in Melzer’s. ***Basidia*** 4-spored, clavate, bulbous top, 20.0–22.0 × 5.5–7.0 μm, hyaline yellow or brown in KOH, tawny yellow in Melzer’s. ***Hymenophoral trama*** divergent, wrinkled, thin-walled, hyaline and mostly 5–10 μm, up to 15 μm. ***Pleuro- and Cheilocystidia*** in fascicles, abundant, clavate, content brown or hyaline, surrounded by brown amorphous materials in KOH, up to 70 μm long. ***Caulocystidia*** abundant in all part of the stipe, morphologically similar with pleuro- and cheilocystidia, encrusted with too much brown pigments, up to 100 μm long. ***Pileipellis*** an ixocutis, hyphae hyaline, thin-walled and encrusted with ochraceous granules, scattered in glue, most 3–7 μm, up to 13 μm wide. *Stipitipellis* mostly composed of interwoven hyphae, covered by brown amorphogous pigments, about 3–9 μm wide. ***Context trama*** hyaline, smooth, thin-walled, interwoven, similar for pileus and stipe, mostly 3–30 μm, up to 50 μm wide at stipe base. ***Clamp connections*** absent.

###### Habitat.

Usually gregarious, in association with *Pinusyunnanensis*.

###### Known distribution.

Currently only known from southwestern China within the range of *Pinusyunnanensis*.

###### Specimens examined.

**China**: • Yunnan Province, Diqing Tibetan Autonomous Prefecture, Shangri-la Town, Xin Sheng Qiao forest park (alt. 3317 m), 17 September 2010, Xiaofei Shi, Shi699 (HKAS 63204); **China**: • Yunnan Province, Heqing Xian, Song Gui Town (alt. 1600 m), 10 October 2011, Xiaofei Shi, Shi1018 (HKAS 71991); **China**: • Yunnan Province, Weixi Town, Tacheng Xiang, Bazhu village (alt. 1900 m), 15 October 2011, Xiaofei Shi, Shi1032 (HKAS 72004); **China**: • Sichuan Province, Xicang City, Puge Town, Luoji Mountain, outside Xianren cave (alt. 2000 m), 12 September 2010, Xiaofei Shi, Shi673 (HKAS 63183).

###### GenBank.

ITS = KU721214; LSU = KU721393; *TEFα-1* = KU721602; *RPB1* = KU852247; *RPB2* = KU852310.

###### Notes.

Sister to this new species, *Suillusluteus* grows in Europe with *Pinussylvestris* and has been introduced to North America along with the host tree. *Suillusluteus* collections from northeastern China associate with P.sylvestrisvar.sylvestriformis and it is the same European *Suillusluteus* species.

##### ﻿ Suillus zangii

Taxon classificationAnimaliaBoletalesSuillaceae

R. Zhang, X.F. Shi, G.M. Mueller & P.G. Liu sp. nov.

1C8DAF7B-C88F-578C-9112-6DAA4FC7DB2B

822266

Figs 12 , 13

###### Etymology.

The species epithet honors Chinese mycologist Mu Zang.

**Figure 12.**
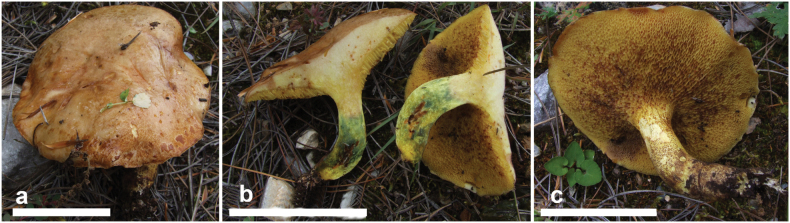
Basidiomata of *Suilluszangii* (holotype, HKAS 63254). Scale bar: 5 cm.

###### Diagnosis.

The basal stipe context turns greenish blue always when exposed.

**Figure 13.**
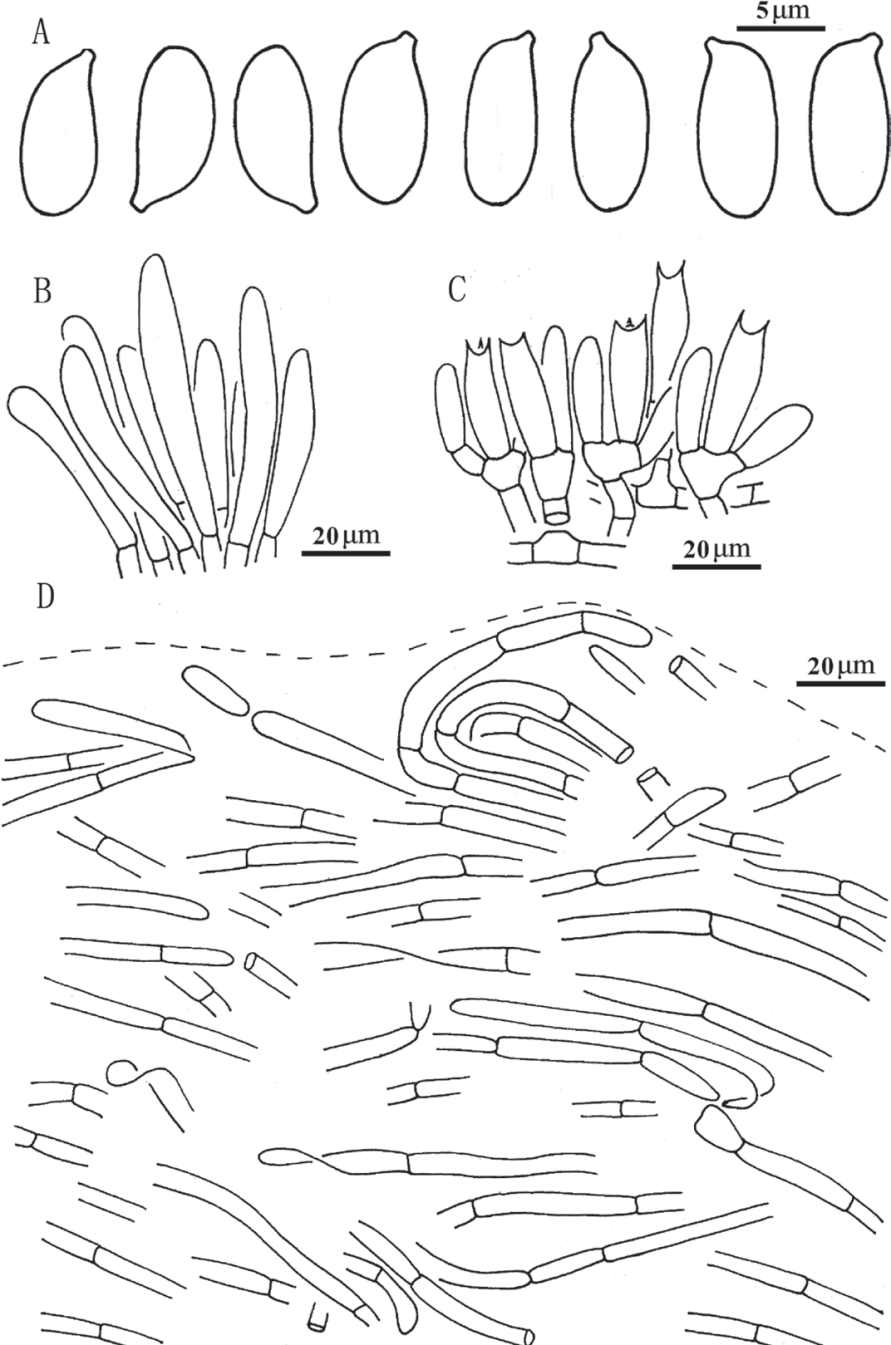
*Suilluszangii* (holotype, HKAS 63254). **a** Basidiospores; **b** Cheilocystidia; **c** Hymenium with basidia; **d** Pileipellis (mature specimens). Scale bars: 5 μm (**a**); 20 μm (**b–d**).

###### Typification.

**China**: • Yunnan Province, Diqing Tibetan Autonomous Prefecture, Shangri-la town, Nixi xiang (28°02'46.19"N, 99°30'16.41"E, alt. 3086 m), 17 September 2010, Xiaofei Shi, Shi703 (holotype, HKAS 63254).

###### GenBank.

ITS = KU721321; LSU = KU721726; *TEFα-1* = KU852220.

###### Morphology.

***Pileus*** hemispherical to convex or flat, 3–8 cm diameter, surface viscid to glutinous in moist, glabrous, with amorphous brown cinnamon (6D5 to 6D7) color dots or patches over brownish yellow (5B4) background. ***Hymenophore*** adnate to subdecurrent, golden yellow (4A6, 4A7). ***Pores*** 1 to 2 per mm, angular. ***Tubes*** 4–6 mm deep, concolorous with pore surface. ***Stipe*** 5–8 × 1.5–2 cm, equal, solid, no veil, background color pallid yellow (3A4, 3A5), more yellowish towards the tube. Covered all over with glandular dots that are in streaks, brown (5E6) dots at apex, reddish brown (6D3) at the base. ***Mycelia*** white. ***Context*** yellowish white to yellow (3A3, 3A4) in pileus and stipe. Change color immediately in lower part of the stipe to greenish blue (25A5, 25B5) when cut. ***Spore print*** unknown. ***Odor and taste*** indistinctive.

***Basidiospores*** [80/2/2] (8.5) 9.5–10.0 × 4.0–5.0 μm, Q = 1.90–2.38, Q_sd_ = 2.14 ± 0.13, smooth, oblong in face view, narrowly inequilateral with a hilar appendage in profile view, brown or ochraceous in KOH, tawny yellow in Melzer’s. ***Basidia*** 4-spored, clavate, bulbous top, 24.0–30.0 × 7.0–8.5 μm, hyaline yellow or brown in KOH, tawny yellow in Melzer’s. ***Hymenophoral trama*** divergent, wrinkled, thin-walled, hyaline and mostly 5–7 μm, up to 13 μm. ***Pleuro- and Cheilocystidia*** in fascicles, abundant, clavate, content brown or hyaline, surrounded by brown amorphous materials in KOH, up to 80 μm long. ***Caulocystidia*** abundant in all part of the stipe, morphologically similar with pleuro- and cheilocystidia, encrusted by brown pigments in KOH, up to 90 μm long. ***Pileipellis*** an ixocutis, hyphae densely encrusted with ochraceous granules, scattered in glue, most 3–10 μm, up to 13 μm wide. ***Stipitipellis*** mostly composed of interwoven hyphae, covered by brown amorphogous pigments, about 3–10 μm wide. ***Context trama*** hyaline, smooth, thin-walled, interwoven, similar for pileus and stipe, mostly 3–30 μm, up to 40 μm wide at stipe base. ***Clamp connections*** absent.

###### Habitat.

Solitary to scattered, in association with *Pinusarmandii*.

###### Known distribution.

Currently only known from Shangri-la town in Yunnan Province, China.

###### Specimens examined.

**China**: • Yunnan Province, Diqing Tibetan Autonomous Prefecture, Shangri-la Town, Nixi Xiang (28°03'03.73"N, 99°29'48.80"E, alt. 3035 m), 17 September 2010, Xiaofei Shi, Shi701 (HKAS 63237); • ibid Shi702 (HKAS 63238).

###### Notes.

This new species is sister with *Suillusmarginielevatus* and *S.indicus* (Sarwar et al. 2015; Verma and Reddy 2015). Geographic ranges and host associations can help separate *Suilluszangii* from the other two. Unlike *Suilluszangii* and *Suillusmarginielevatus*, *S.indicus* has a white and membranous ring on the stipe, appendiculated cap margin, and no glandular dots. Also, *Suillusindicus* and *S.marginielevatus* are both in association with *Pinuswallichiana*. So it would be interesting to know, excluding observational errors, reasons for the presence or absence of veil and glandular dots not conserved among closely related species.

##### ﻿ Suillus sect. Diversipedes

Taxon classificationAnimaliaBoletalesSuillaceae

R. Zhang, X.F. Shi, G.M. Mueller & P.G. Liu sect. nov.

D31A82D7-DCF9-546C-B1E3-119B223C6B2F

822260

###### Etymology.

This section contains a large number of species and is morphologically very diverse. Therefore, the derivation is from *diversi*- (diverse) and *pes* (foot).

###### Diagnosis.

Suillussect.Diversipedes is distinguished by its striking morphological diversity. Key feature is that sect. Diversipedes form ectomycorrhizal primarily with *Pinus* (rarely *Quercus*). Morphological identifications include: stipes with or without glandular dots (variable in color); partial veil (if present) fibrillose, gelatinous, or membranous; spores olive to cinnamon-brown.

###### Typification.

*Suillustomentosus* Singer, Snell & E.A. Dick, Mycologia 51(4): 570 (1960) [1959]

###### Morphology.

***Basidiomata*** stipitate-pileate with tubular hymenophore. ***Pileus*** develops from hemispherical to convex, plane or umbonate, sometimes with wavy or recurved margin, viscid to glutinous, or dry, glabrous or covered with fibrils, squamules or scales. Background color ivory to yellow. Some species are covered with pinkish, brown, red or yellow fibrils, appressed squamules or scales. Pileus contains colored scales and glutinous layers yellow, yellowish white, brown, dark brown, olive brown, olive, pinkish, red, or cinnamon. ***Hymenophore*** adnate, subdecurrent or decurrent, Pores 1–2 per mm, large ones up to 5 mm diameter, round to angular, radially arranged to almost lamellate. Younger ones of some species beaded with white or yellowish droplets. Sometimes changing color to brownish or light blue when bruised or cut. ***Context*** white, whitish yellow, yellow or light orange in pileus and stipe. Stipe context sometimes changing color to blue, greenish blue, reddish or brownish. Stipe equal to clavate, solid, with or without glandular dots, glandular dots whitish, yellow, reddish, brown, or cinnamon brown when young, become cinnamon brown or brown with age. With or without veils, veils often superior, white, pinkish or brownish, persistent or evanescent, fibrillose, cottony, gelatinous, glutinous or membranous. ***Mycelia*** always white or pinkish, sometimes changing color to pinkish or red when bruised. ***Spore print*** olive, brown, cinnamon brown or olive brown.

***Basidiospores*** smooth, oblong and inequilateral, hyaline yellow to ochraceous brown in KOH, usually 7–11 μm. ***Basidia*** 4-spored, clavate, hyaline yellow in KOH. ***Cystidia*** abundant, typically fasciculate, large, up to 100 μm, with brown contents and surrounded by brown amorphous materials in KOH, some species lack caulocystidia. ***Pileipellis*** a layer of gelatinous hyphae, with yellowish hyaline content in KOH; some species with a layer of scales that are smooth and light ochraceous in KOH. ***Clamp connections*** absent.

###### Habitat.

Scattered to gregarious, ectomycorrizal with *Pinus*.

###### Known species.

*Suillusacerbus*, *S.acidus*, *S.americanus*, *S.boletoluteus*, *S.bovinus*, *S.cinerescens*, *S.cothurnatus*, *S.decipiens*, *S.discolor* (possibly), *S.flavidus*, *S.fuscotomentosus*, *S.helenae*, *S.himalayensis*, *S.hirtellus*, *S.kwangtungensis*, *S.megaporinus*, *S.minusculus*, *S.phylopictus*, *S.phylosubaureus*, *S.pinetorum*, *S.plorans*, *S.punctipes*, *S.salmonicolor*, *S.sibiricus*, *S.spraguei*, *S.subalutaceus*, *S.subaureus*, *S.subcinnamomeus*, *S.subolivaceus*, *S.subsibiricus*, *S.suilloides*, *S.tomentosus*, and *S.umbonatus*.

###### Notes.

This section is morphologically very diverse. Pileus dry or viscid to glutinous, glabrous or fibrillose to scaly, cap colors variable, colors include yellow, yellowish white, brown, dark brown, olive brown, olive, pinkish, red, and cinnamon. Stipe background color also variable, concolorous or not with pileus. Glandular dots present or not, when present color varies from white, yellow, reddish, brownish. Partial veil present or absent, when present either as appendiculate margin or if an annulus then fibrillose, gelatinous, glutinous or membranous, persistent or evanescent. Spore deposits brown, olive brown, or reddish brown. Hosts of this section include *Pinus* subgenera *Pinus* and *Strobus*. *Suillussubaureus* is capable of forming association with *Quercus*.

This section contains numerous potential new or cryptic species. For instance, *Suilluscinerescens* contains cryptic species with distinct morphological features waiting to be confirmed with more collections. *Suilluspinetorum*, *S.plorans* and *S.subsibiricus* contain geographic cryptic species. An unknown species from Russia, sisters to *S.subcinnamomeus* is identified in the ITS phylogeny as a potential new species. More species complexes are found in this section compared with other section and subgenera. Resolving species in section Diversipedes requires large sample collections and phylogenetically informative sequences.

##### ﻿ Suillus boletoluteus

Taxon classificationAnimaliaBoletalesSuillaceae

R. Zhang, X.F. Shi, G.M. Mueller & P.G. Liu. sp. nov.

4568B1D0-823F-580A-9B68-B64C725D18E2

822267

Figs 14 , 15

###### Etymology.

The species resembles *Suillusluteus* and “boleti-” indicates its smooth and regular hymenium like species in *Boletinus*.

**Figure 14.**
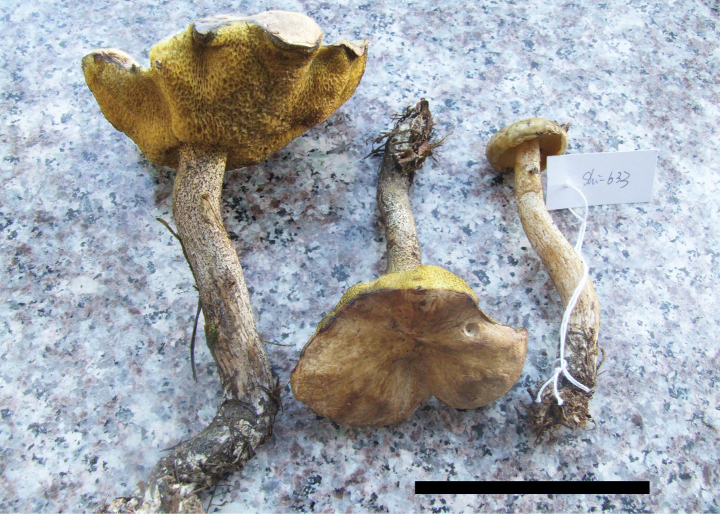
Basidiomata of *Suillusboletoluteus* (holotype, HKAS 63244). Scale bar: 5 cm.

###### Diagnosis.

*Suillusboletoluteus* has smooth and regular hymenium. The pores and stipes usually change color to greenish blue when cut. This species is in association with Pinussubg.Strobus in southwestern China and subg. Pinus in northeastern China.

**Figure 15.**
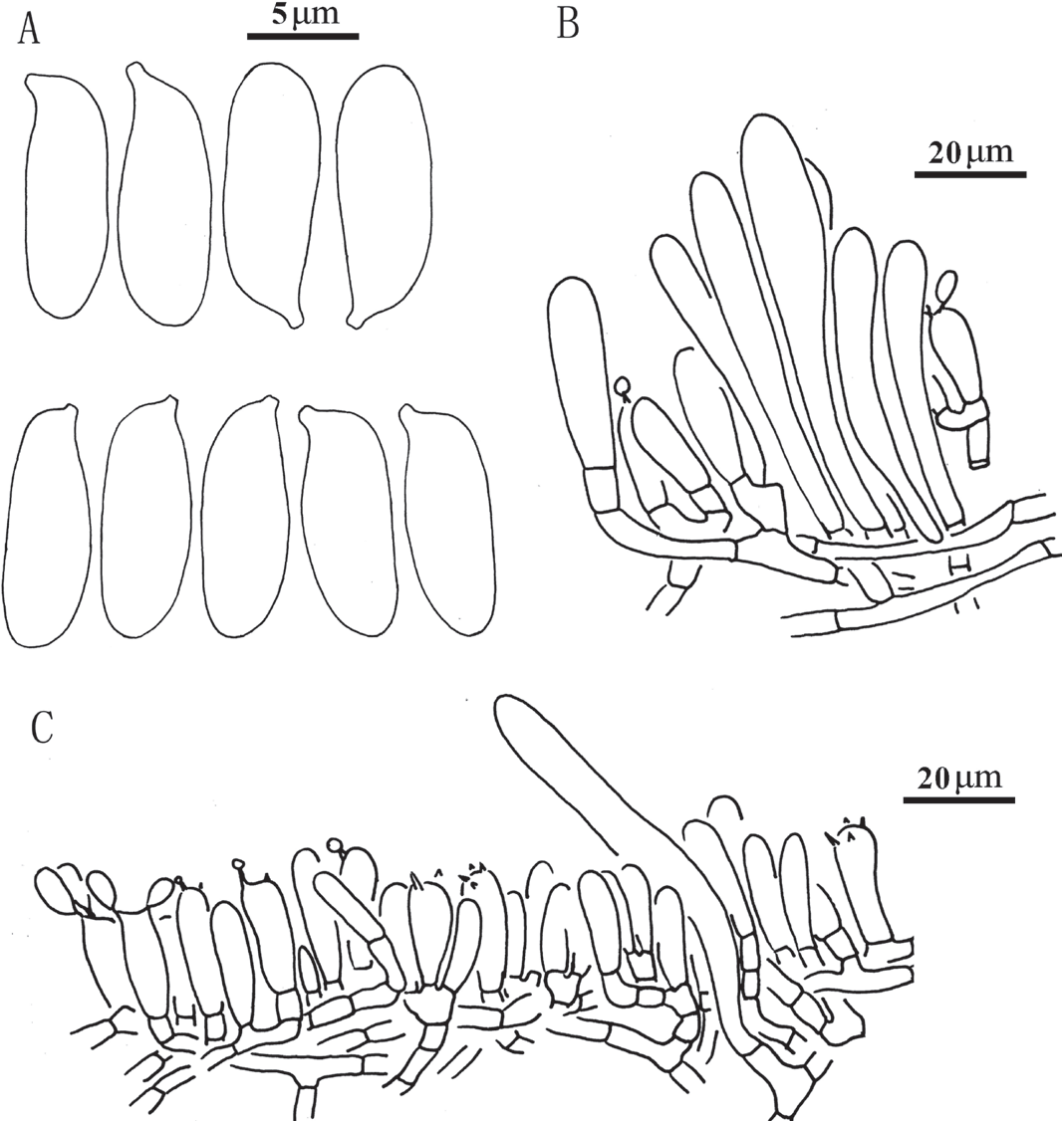
*Suillusboletoluteus* (holotype, HKAS 63244). **a** Basidiospores; **b** Cheilocystidia and some basidia on tube surface; **c** Basidia and pleurocystidia. Scale bars: 5 μm (**a**); 20 μm (**b–d**).

###### Typification.

**China**: • Inner Mongolia, Hulun Buir, Erguna, Moerdaoga National Forest Park (51°23'16.40"N, 120°45'20.64"E, alt. 1000 m), 27 August 2010, Xiaofei Shi, Shi633 (holotype, HKAS 63244).

###### GenBank.

ITS = KU721499; LSU = KU721374; *TEFα-1* = KU721702

###### Morphology.

***Pileus*** develops from convex to plane or recurved, margin wavy at maturity, 3–9 cm diameter, surface viscid to glutinous, glabrous, background color yellowish buff (4B4), with brownish color streaks (6D5, 6E5) underneath the gluten. Younger ones white cap with buff brownish glue (5C4). Appendiculated with white and membranous partial veils that fall off with age. ***Hymenophore*** adnate to narrowly adnate, yellow (3B7). ***Pores*** 1–2 per mm, angular, compound. Pore surface covered with brownish cinnamon glandular dots (6F4–6F7). Change color to greenish blue (24A4) when cut or bruised. ***Tubes*** 4–7 mm deep. Stipe 6–9.5 × 0.8–1.2 cm, equal or slightly tapering towards apex, solid, cuticle background color greyish white, covered all over with glandular dots that are big and dense in streaks, dots are buff brown (5B3, 5B4), or cinnamon brown (6C4, 6C5) in younger ones. A superior gelatinous veil present, white and membranous, fall off at maturity or leave some brownish (6C4–6D4) membranous traces on the stipe. ***Context*** white to yellowish. Sometimes pileus context turn light blue when cut. ***Spore print*** brown. ***Odor and taste*** indistinctive.

***Basidiospores*** [40/2/2] 9.5–12.0 (13.5) × 3.5–4.0 μm, Q = (2.38) 2.50–3.43 (3.71), Q_sd_ = 2.81 ± 0.34, smooth, oblong in face view, narrowly inequilateral with a hilar appendage in profile view, brown or ochraceous in KOH, tawny yellow in Melzer’s. ***Basidia*** 4-spored, clavate, bulbous top, 20.0–26.0 × 7.5–8.5 μm, hyaline yellow or brown in KOH, tawny yellow in Melzer’s. ***Hymenophoral trama*** divergent, wrinkled or smooth, thin-walled, hyaline and mostly 3–8 μm wide. ***Pleuro- and Cheilocystidia*** in fascicles, abundant, clavate, content brown or hyaline, surrounded by brown amorphous materials in KOH, up to 70 μm long. ***Caulocystidia*** abundant in all part of the stipe, morphologically similar with pleuro- and cheilocystidia, fascicle base encrusted by profuse brown pigments in KOH, up to 130 μm long. ***Pileipellis*** a gelatinous layer with some hyphae encrusted by ochraceous granules, some hyphae are not encrusted, most 2–8 μm, up to 11 μm wide. ***Stipitipellis*** mostly composed of interwoven hyphae, covered by brown amorphogous pigments, about 3–10 μm wide. ***Context trama*** hyaline, smooth, thin-walled, interwoven, similar for pileus and stipe, mostly 3–30 μm, up to 40 μm wide at stipe base. ***Clamp connections*** absent.

###### Habitat.

Solitary to scattered, in association with Pinussylvestrisvar.mongolica and *Pinusarmandii*.

###### Known distribution.

Currently only known from northeastern and southwestern China.

###### Specimens examined.

**China**: • Yunnan Province, Jianchuan Town, Lao Jun Mountain (alt. 3400 m), 8 September 2009, Xiaofei Shi, Shi284 (HKAS 71797) • ibid 12 September 2009, Xiaofei Shi, Shi289 (HKAS 71802).

###### Notes.

This species is sister to North American *Suillusacidus*. The two sibling species are morphologically very similar. It is interesting that *Suillusboletoluteus* is in association with Pinussubg.Strobusin southwestern China andsubg.Pinus in northeastern China. Host associations of the new species should be further confirmed. In addition, it is possible that *S.boletoluteus* contains cryptic species as indicated by the long branches in the *TEFα-1*, *RPB1* and *RPB2* phylogenies.

##### ﻿ Suillus cinerescens

Taxon classificationAnimaliaBoletalesSuillaceae

R. Zhang, X.F. Shi, G.M. Mueller & P.G. Liu. sp. nov.

313D6DA9-9F18-50FE-B90D-4B54F8342CA5

822268

Figs 16 , 17

###### Etymology.

The epithet indicates the grey color of the pileus.

**Figure 16.**
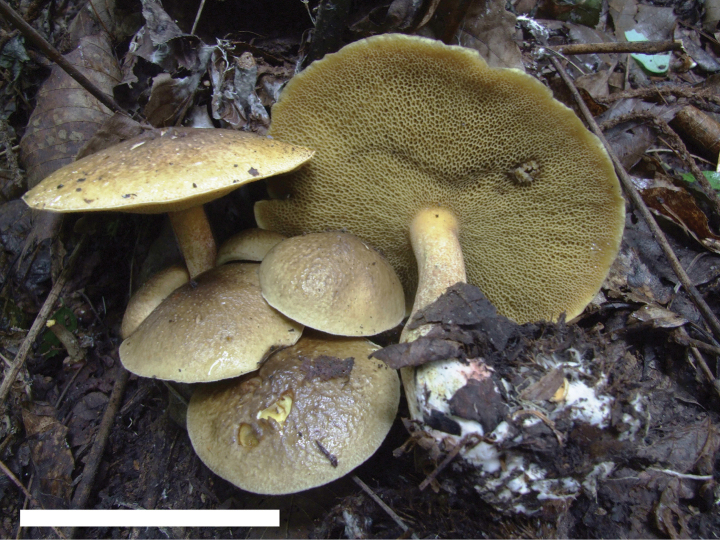
Basidiomata of *Suilluscinerescens* (holotype, HKAS 63243). Scale bar: 5 cm.

###### Typification.

**China**: • Shaanxi Province, Baoji City, Mei Xian, Yingtou Town, Hao Ping Dali village (alt. 1300 m), 4 September 2010, Xiaofei Shi, Shi664 (holotype, HKAS 63243).

**Figure 17.**
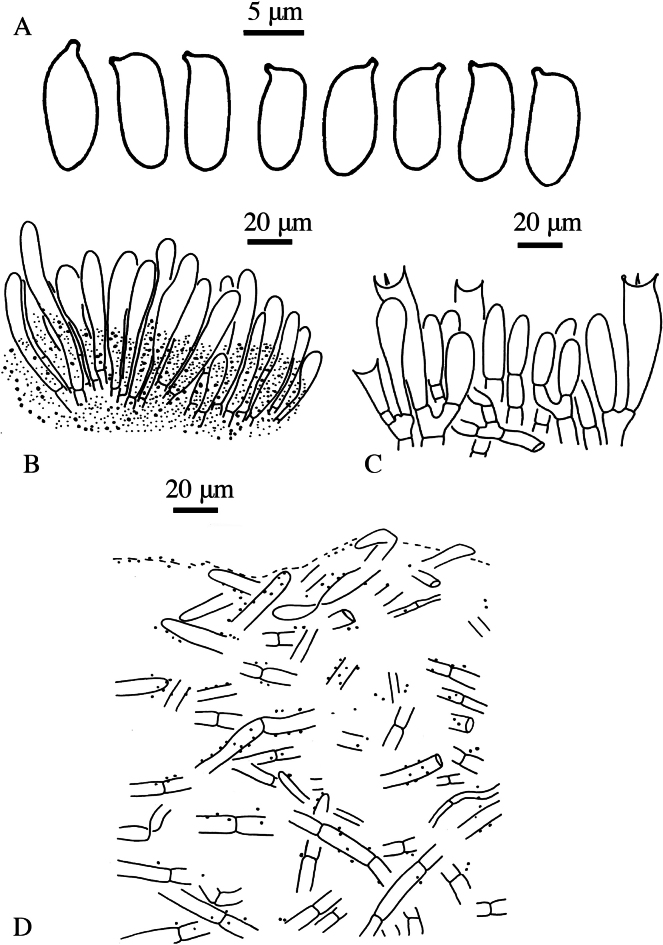
*Suilluscinerescens* (holotype, HKAS 63243). **a** Basidiospores; **b** Cheilocystidia; **c** Hymenium with basidia; **d** Pileipellis (mature specimens). Scale bars: 5 μm (**a**); 20 μm (**b–d**).

###### Diagnosis.

*Suilluscinerescens* has grey pileus, is in association with *Pinusarmandii* in central and southwestern China.

###### GenBank.

ITS = KU721159; LSU = KU721317; *TEFα-1* = KU721740; *RPB1* = KU852278.

###### Morphology.

***Pileus*** develops from hemispherical to convex or plane, 4–9 cm diameter, surface viscid to glutinous, glabrous, background color yellowish brown (4B4), younger ones with dark olive glue (3C4, 3C5), discoloring to greyish (3B3) when old. ***Hymenophore*** adnate to narrowly adnate, younger ones dull cinnamon (5C4, 5C5), turn to olive yellow (3B6, 3C6) with age. Do not change color when bruised. ***Pores*** 1 per mm, angular, compound. ***Tubes*** 4–9 mm deep, concolorous with pores. ***Stipe*** 6–10 × 0.9–2 cm, equal or tapering towards apex, solid, no ring, cuticle color yellowish white (3A3), covered all over with reddish brown (4A6, 4A7) glandular dots. ***Mycelia*** white, turn to red (6A3, 7A3) when bruised. ***Context*** white to yellowish. Do not change color when exposed. ***Spore print*** olive brown. ***Odor and taste*** indistinctive.

***Basidiospores*** [40/2/2] (8.5) 9.0–10 × (3.5) 4.0–4.5 μm, Q =(2.22) 2.25–2.50, Q_sd_ = 2.4 ± 0.13, smooth, oblong in face view, narrowly inequilateral with a hilar appendage in profile view, brown or uncommonly hyaline in KOH, dark ochraceous in Melzer’s. ***Basidia*** 4-spored, clavate, bulbous top, 20.0–25.0 × 7.0–8.0 μm, contain brown pigments or uncommonly hyaline in KOH. ***Hymenophoral trama*** divergent, wrinkled, thin-walled, hyaline and mostly 5–11 μm, up to 15 μm. ***Pleuro- and cheilocystidia*** in fascicles, abundant, clavate, up to 70 μm long, content brown or hyaline, surrounded by brown amorphous materials in KOH. ***Caulocystidia*** abundant, morphologically similar with pleuro- and cheilocystidia, in fascicles with over 10 caulocystidia, surrounded by brown pigments, up to 100 μm. ***Pileipellis*** encrusted by fine ochraceous granules, hyphae scattered in yellowish glue in KOH, most 3–7 μm, up to 11 μm wide. ***Stipitipellis*** covered by profuse brown amorphous pigments, composed of interwoven hyphae, about 3–9 μm wide. ***Context trama*** hyaline, smooth, thin-walled, interwoven, similar in pileus and stipe, mostly 5–50 μm, up to 110 μm wide at stipe base. ***Clamp connections*** absent.

###### Habitat.

Usually gregarious, in association with *Pinusarmandii*.

###### Known distribution.

Currently known from central and southwestern China.

###### Specimens examined.

**China**: • Hubei Province, Shen Nong Jia Forest, Honghua Duo town (alt. 1600 m), 31 July 2011, Xiaofei Shi, Shi763 (HKAS 71893); **China**: • Yunnan Province, Lijiang City, Xuesong village (alt. 2700 m), 27 August 2009, Gang Wu, GangWu 155 (HKAS 57687); **China**: • Heilongjiang Province, Yichun City, Wuying National Forest Park (alt. 400 m), 17 August 2010, Xiaofei Shi, Shi558 (HKAS 63234); **China**: • Heilongjiang Province, Da Xing Anling, Huzhong Town, Hua road (alt. 1000 m), associated with *Pinuspumila*, 21 August 2010, Xiaofei Shi, Shi588 (HKAS 63225).

###### Notes.

Two collections (HKAS 63234 and 63225) are excluded from the new species description because they are strongly indicated as different species by phylogenetic, morphological and ecological evidence. The collection “HKAS 63234” from northeastern China has strong bluing reaction when the hymenium is bruised and it associates with *Pinuskoraiensis*. Another collection “HKAS 63225” has more reddish cinnamon in pileus color, and it associates with *Pinuspumila*. However, more collections are needed to confirm these observations and to support the cryptic species. The *Suilluscinerescens* collections associated with *Pinusarmandii* are morphologically similar with closely related species *S.plorans* from Europe.

##### ﻿ Suillus minusculus

Taxon classificationAnimaliaBoletalesSuillaceae

R. Zhang, X.F. Shi, G.M. Mueller & P.G. Liu sp. nov.

2099BAD0-650E-528C-B94F-7573A44BDE52

822269

Figs 18 , 19

###### Etymology.

The epithet refers to the small size of the species.

**Figure 18.**
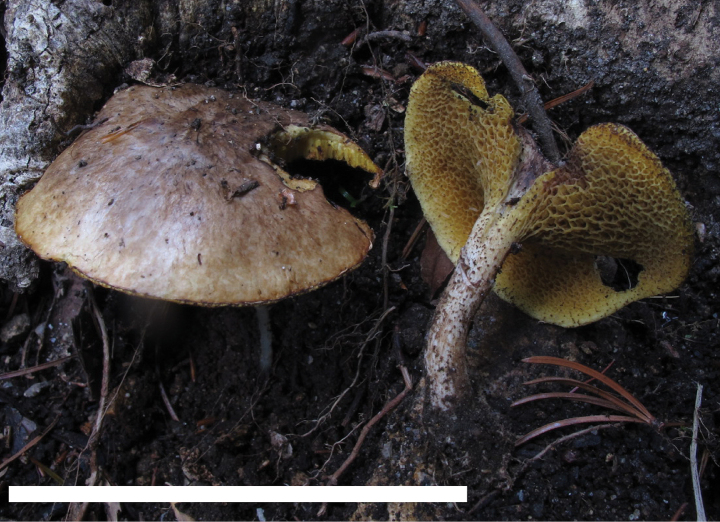
Basidiomata of *Suillusminusculus* (paratype, HKAS 71980). Scale bar: 5 cm.

###### Diagnosis.

The diameter of the pileus is 3–4.5 cm and it is among the smallest of known *Suillus* species.

**Figure 19.**
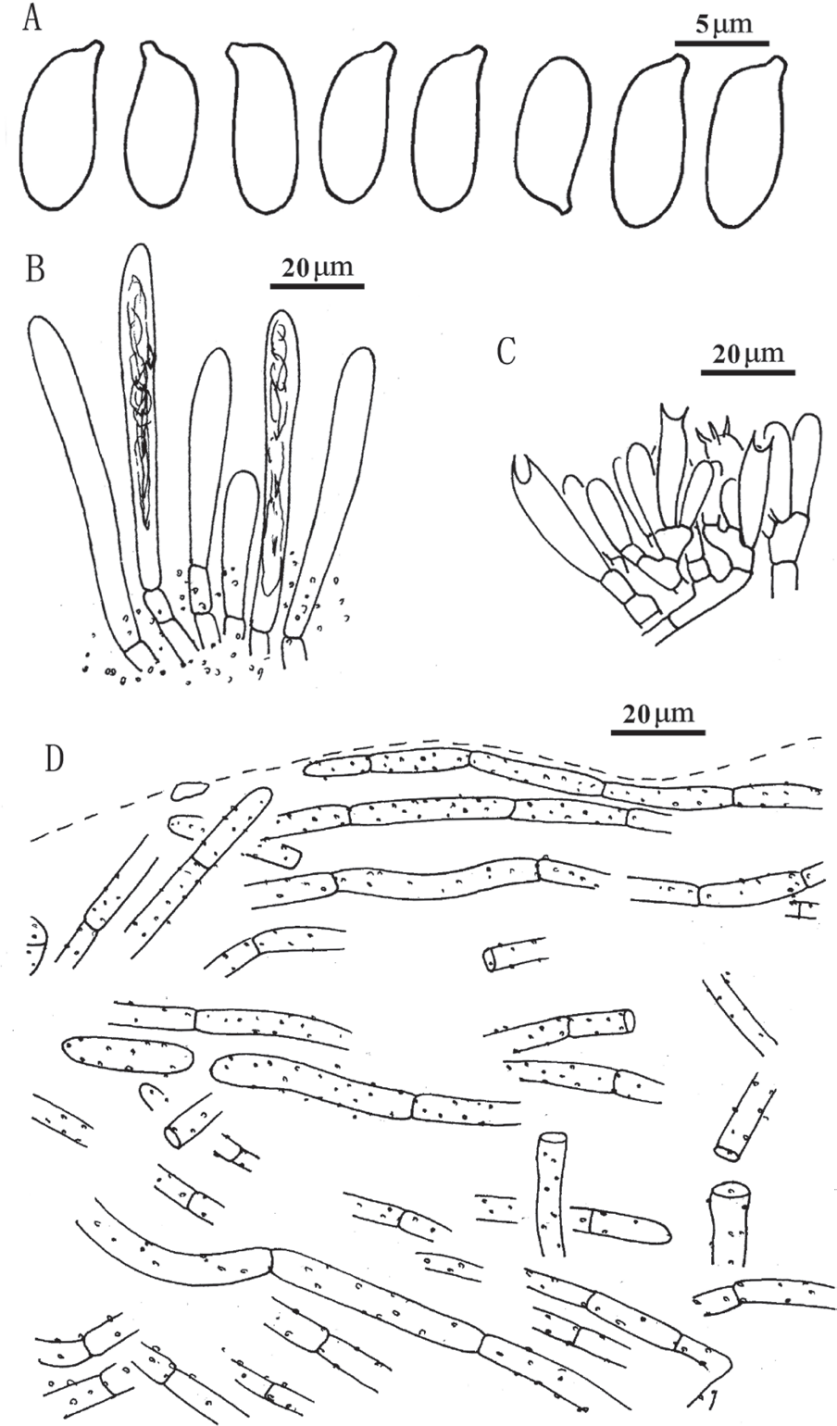
*Suillusminusculus* (paratype, HKAS 71980). **a** Basidiospores; **b** Cheilocystidia; **c** Hymenium with basidia; **d** Pileipellis (mature specimens). Scale bars: 5 μm (**a**); 20 μm (**b–d**).

###### Typification.

**China**: • Guangdong Province, Ruyuan, Shaoguan, Nanling National Forest Park (24°53'50.10"N, 113°01'37.22"E, alt. 1200 m), 18 September 2015, Bo Li, Libo113 (holotype, HKAS 89874).

###### GenBank.

ITS = KU721492; LSU = KY489974; *TEFα-1* = KU721706; *RPB1* = KU852193; *RPB2* = KU852352.

###### Morphology.

***Pileus*** develops from convex to plane, eventually depressed, 3–4.5(-5) cm diameter, surface viscid when moist, background color yellowish brown (4A4, 4B4), covered with closely appressed dark brown (5E7, 5E8). ***Hymenophore*** decurrent, yellow (4A7, 4B7), pores comparatively large, 1 per mm, angular, compound, radially arranged, smaller towards the margin. ***Tubes*** 3–5 mm long, concolorous with the pores. ***Stipe*** 3.0–4.0 × 0.3–0.5 cm, cylindrical, solid, veil white and membranous, turning blackish when bruised, lost in age. Reticulate above the annulus. Glandular dots abundant, distribute mainly below the annulus, vinaceous brown (6D7). ***Context*** yellow in pileus and stipe, does not change color. Pileus context thin, no more than 4 mm thick. ***Spore print*** reddish brown (6E5). ***Odor and taste*** indistinctive.

***Basidiospores*** [80/1/2] (8.5) 9.0–11.0 × 3.5–4.5 μm, Q = 2.13–2.50 (2.57), Q_sd_ = 2.36 ± 0.13, smooth, oblong in face view, narrowly inequilateral with a hilar appendage in profile view, brown or ochraceous in KOH, tawny yellow in Melzer’s. ***Basidia*** 4-spored, clavate, bulbous, 21.0–28.0 × 6.0–8.5 μm, hyaline yellow or brown in KOH, tawny yellow in Melzer’s. ***Hymenophoral trama*** divergent, wrinkled, thin-walled, hyaline and mostly 5–8(-12) μm. ***Pleuro- and Cheilocystidia*** in fascicles, abundant, clavate, contents brown or hyaline, surrounded by brown amorphous material in KOH, up to 80 μm long. ***Pileipellis*** outer most a layer of hyphae light ochraceous or hyaline in KOH, smooth, most 3–7 μm; underneath a gelatinous layer with hyphae densely encrusted with ochraceous granules, most 3–7(-11) μm wide. ***Stipitipellis*** mostly composed of interwoven hyphae, covered by brown amorphogous pigments, about 3–10 μm wide. ***Caulocystidia*** abundant along stipe, morphologically similar with pleuro- and cheilocystidia, encrusted with brown pigments in KOH, up to 100 μm long. ***Context trama*** hyaline, smooth, thin-walled, interwoven, similar in pileus and stipe, mostly 3–30 μm, up to 40 μm wide at stipe base. ***Clamp connections*** absent.

###### Habitat.

Scattered, in association with *Pinuskwangtungensis*.

###### Known distribution.

Currently only known from the Nanling National Forest Park in Guangdong.

###### Specimens examined.

**China**: • Guangdong Province, Ruyuan, Shaoguan, Nanling National Forest Park (24°53'47.02"N, 113°01'36.05"E, alt. 1209 m), 18 September 2015, Rui Zhang, Rui363 (HKAS 90665); *ibid* 17 September 2011, Xiaofei Shi, Shi979 (HKAS 71980).

###### Notes.

*Pinuskwangtungensis* is a rare pine species and forms a large forest only in the Nanling National Forest Park; it seems that *S.minusculus* is also rare and should be under conservation.

##### ﻿ Suillus phylosubaureus

Taxon classificationAnimaliaBoletalesSuillaceae

R. Zhang, X.F. Shi, G.M. Mueller & P.G. Liu sp. nov.

46A6DE82-61E7-56A4-8F23-67F00387926F

822270

Figs 20 , 21

###### Etymology.

This species is sister to *Suillussubaureus* and identified first by molecular phylogeny.

**Figure 20.**
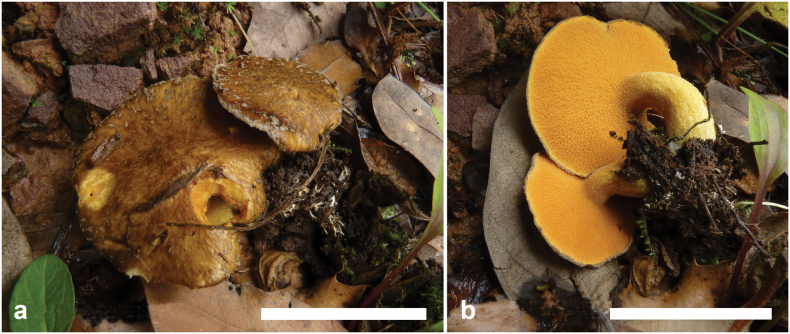
Basidiomata of *Suillusphylosubaureus* (holotype, HKAS 71798). Scale bar: 5 cm.

###### Typification.

**China**: • Yunnan Province, Jianchuan City, Lao Jun Mountain (alt. 3400 m), 8 September 2009, Xiaofei Shi, Shi285 (holotype, HKAS 71798).

**Figure 21.**
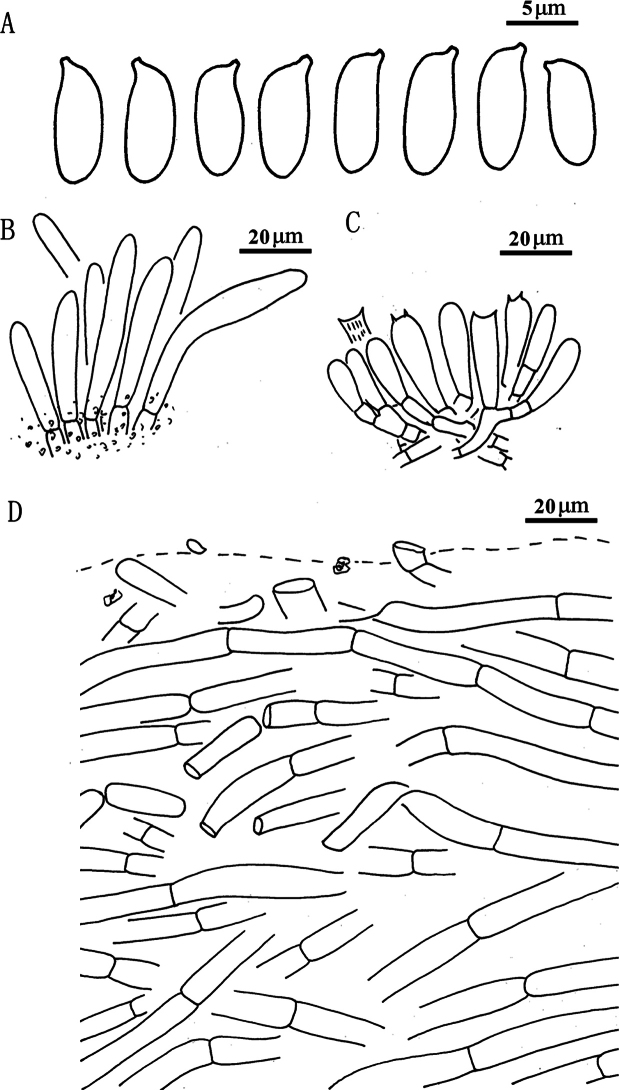
*Suillusphylosubaureus* (holotype, HKAS 71798). **a** Basidiospores; **b** Cheilocystidia; **c** Hymenium with basidia; **d** Pileipellis (mature specimens). Scale bars: 5 μm (**a**); 20 μm (**b–d**).

###### GenBank.

ITS = KU721174; LSU = KU721369; *TEFα-1* = KU721696.

###### Morphology.

***Pileus*** develops from hemispherical to plane, margin wavy at maturity, 3–8 cm diameter, surface viscid in moist, appressed blackish brown scales (6F6, 6F7) underneath the glue, becoming patched and almost glabrous when old. ***Hymenophore*** subdecurrent to decurrent, younger ones bright yellow (4A7, 4A8), turn to brownish yellow with age (4B7). Do not change color when bruised. ***Pores*** 1–2 per mm, angular, compound, arranged as if inconspicuously lamellate, smaller towards the margin. ***Tubes*** 3–6 mm deep, concolorous with pores. ***Stipe*** 5–10 × 0.8–1.5 cm, equal to slightly clavate, solid, no veil, white background, yellow at apex, covered with small glandular dots that are yellow (4A4, 4A5) when young, become brown (6E4) when old. ***Mycelia*** white, do not change color when bruised. ***Context*** yellowish white in pileus, slightly blue around worm holes. In stipe pallid yellow, turn blue (23A4) when cut. ***Spore print*** unknown. ***Odor and taste*** indistinctive.

***Basidiospores*** [40/2/2] 8.0–9.0 × 3.5–4.0 μm, Q = 2.25–2.57, Q_sd_ = 2.35 ± 0.11, smooth, oblong in face view, narrowly inequilateral with a hilar appendage in profile view, brown or ochraceous in KOH, tawny yellow in Melzer’s. ***Basidia*** 4-spored, clavate, bulbous top, 24.0–28.0 × 7.0–8.5 μm, hyaline yellow or brown in KOH, tawny yellow in Melzer’s. ***Hymenophoral trama*** divergent, wrinkled or smooth, thin-walled, hyaline and mostly 3–8 μm wide. ***Pleuro- and Cheilocystidia*** in fascicles, abundant, clavate, content brown or hyaline, surrounded by brown amorphous materials in KOH, up to 70 μm long. ***Caulocystidia*** abundant in all part of the stipe, morphologically similar with pleuro- and cheilocystidia, fascicle base encrusted by profuse brown pigments in KOH, up to 90 μm long. ***Pileipellis*** a layer of scale hyphae, not encrusted, ochraceous, smooth, half of each hypha (4–9 μm) shrink into a thinner strand (1–2 μm). ***Stipitipellis*** mostly composed of interwoven hyphae, covered by brown amorphogous pigments, about 3–10 μm wide. ***Context trama*** hyaline, smooth, thin-walled, interwoven, similar for pileus and stipe, mostly 3–30 μm, up to 40 μm wide at stipe base. ***Clamp connections*** absent.

###### Habitat.

Scattered to gregarious, grows in mixed forest and probably associates with *Pinusarmandii*.

###### Known distribution.

Currently only known from subalpine region of Yunnan Province, China.

###### Specimens examined.

**China**: • Yunnan Province, Jianchuan City, from Li Cha village to Lao Jun Mountain (alt. 3400 m), 2 September 2009, Bang Feng, Bang-Feng763 (HKAS 57492); **China**: • Yunnan Province, Jianchuan City, Lao Jun Mountain (alt. 3400 m), 8 September 2009, Xiaofei Shi, Shi288 (HKAS 71801); **China**: • Yunnan Province, Deqen, Shangri-la Town, Haba snow Mountain (alt. 4900 m), September 2008, Yanchun Li, Yanchun-Li1476 (HKAS 56316).

###### Notes.

This new species is sister to North American *Suillussubaureus*. The two species differ slightly in morphology including colors of the cap. Host association of this species needs to be further studied. As for *Suillussubaureus*, five needle pines are requisites for the establishment of seedlings. But it is unknown about the host species of *S.phylosubaureus* and whether five needle pines are required in any of its developmental stages.

##### ﻿ Suillus subcinnamomeus

Taxon classificationAnimaliaBoletalesSuillaceae

R. Zhang, X.F. Shi, G.M. Mueller & P.G. Liu sp. nov.

417F5DF1-AEF2-5B11-803C-2907E9977F1B

822271

Figs 22 , 23

###### Etymology.

The cap color of this species is cinnamon.

**Figure 22.**
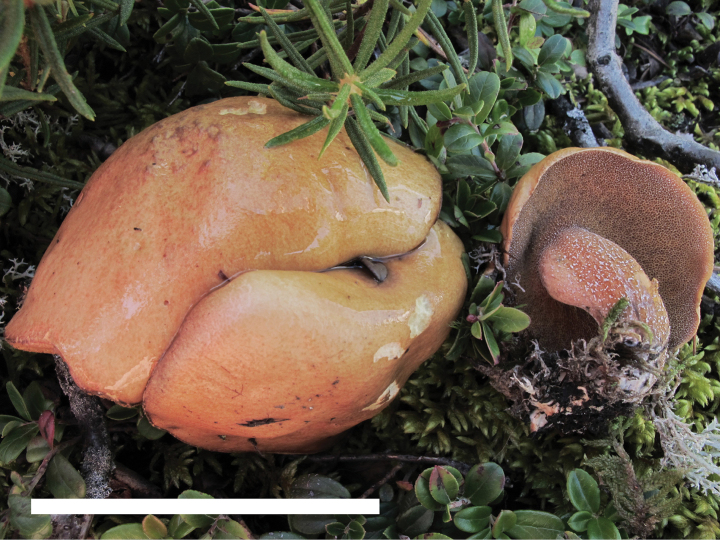
Basidiomata of *Suillussubcinnamomeus* (holotype, HKAS 63222) Scale bar: 5 cm.

###### Diagnosis.

*Suillussubcinnamomeus* has cinnamon color pileus and ivory white glandular dots when young. This species is in association with *Pinuspumila* in northeastern China.

**Figure 23.**
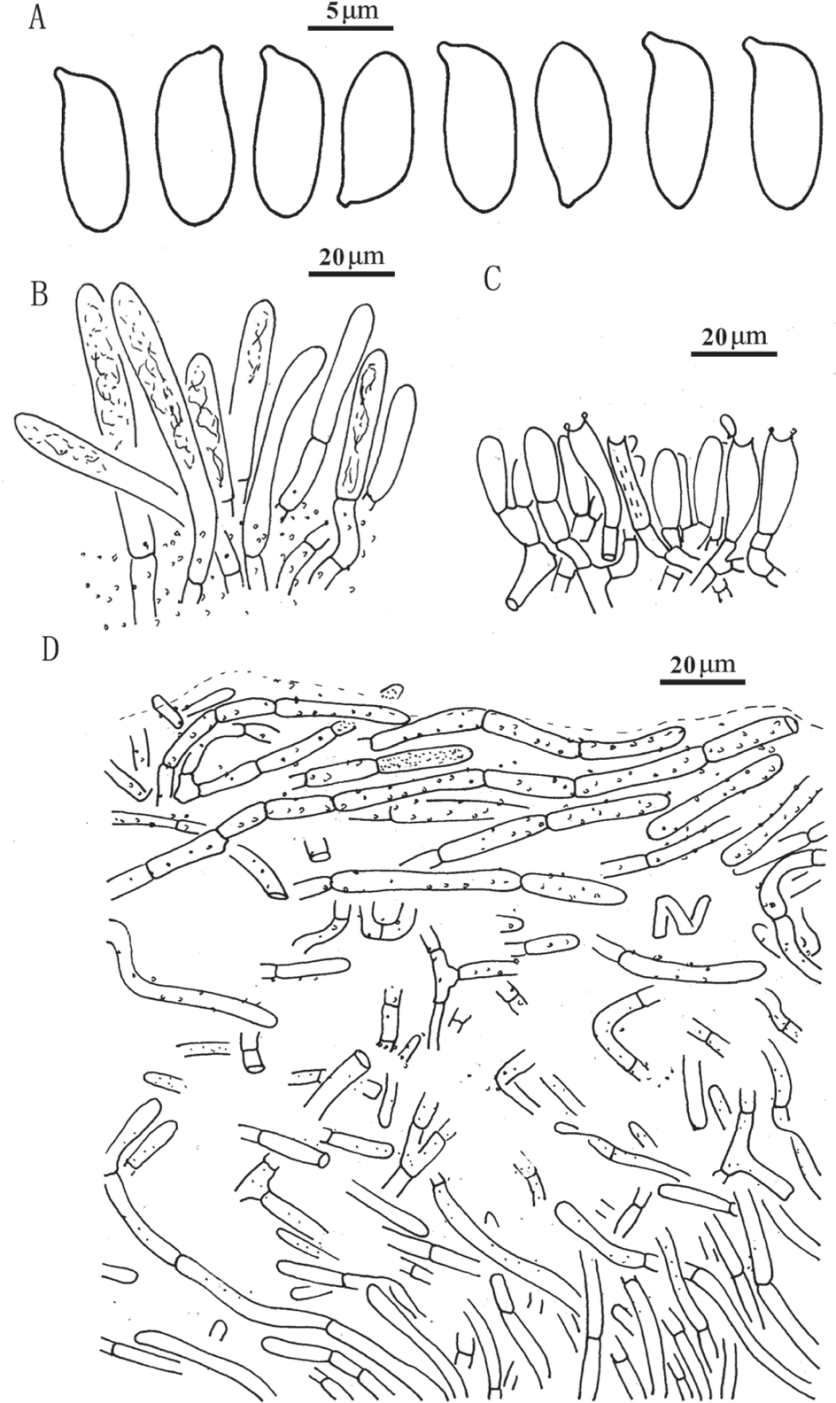
*Suillussubcinnamomeus* (holotype, HKAS 63222). **a** Basidiospores; **b** Cheilocystidia; **c** Hymenium with basidia; **d** Pileipellis (mature specimens). Scale bars: 5 μm (**a**); 20 μm (**b–d**).

###### Typification.

**China**: • Heilongjiang Province, Da Xing Anling, Dabai Mountain (alt. 1000 m), 22 August 2010, Xiaofei Shi, Shi601 (holotype, HKAS 63222).

###### GenBank.

ITS = KU721496.

###### Morphology.

***Pileus*** convex or obtuse, 4–7 cm diameter, surface viscid to glutinous, glabrous, background color yellow (4A6) or light cinnamon (5A5), small color streaks of bright reddish cinnamon (6A7, 6A8) resemble fibrils covering all over the cap. ***Hymenophore*** adnate, younger ones dark cinnamon (6D3–6D5), become more olive brown (5E5, 5E6) with age. Do not change color when bruised. ***Pores*** 2 per mm, round or angular. ***Tubes*** 3–6 mm deep, concolorous with pores. ***Stipe*** 6.5–8.5 × 1.2–1.8 cm, bulbous at base or clavate, solid, concolorous with pileus, with more reddish brown or cinnamon (5E6 or 6C7) color streaks. Glandular dots all over, ivory white (5A1) when young, become reddish brown (5E6) or brown (5F6) with age. ***Mycelia*** pinkish (6A5, 6A6) ***Context*** white to yellowish in pileus and stipe, light cinnamon (5A2, 5A3) close to the cuticles. Do not change color when exposed. ***Spore print*** unknown. ***Odor and taste*** indistinctive.

***Basidiospores*** [40/2/2] 8.5–10.0 (10.5) × 3.5–4.5 μm, Q = 2.13–2.57, Q_sd_ = 2.34 ± 0.13, smooth, oblong in face view, narrowly inequilateral with a hilar appendage in profile view, hyaline to yellowish in KOH, tawny yellow in Melzer’s. ***Basidia*** 4-spored, clavate, bulbous top, 23.0–32.0 × 6.2–7.5 μm, hyaline to yellowish in KOH, tawny yellow in Melzer’s. ***Hymenophoral trama*** divergent, thin-walled, very wrinkled, encrusted with some ochraceous granules in KOH, usually 6–8 μm. ***Pleuro- and Cheilocystidia*** abundant, in fascicles, clavate, up to 70 μm long, content brown or hyaline, surrounded by brown amorphous materials in KOH. ***Caulocystidia*** similar with pleuro- and cheilocystidia, septated once or twice especially at the base. ***Pileipellis*** a layer of scattered hyphae in glue, densely encrusted with fuzzy appearance and surrounded by tiny brown granules in KOH, most hyphae 5–7 μm, up to 8.5 μm wide. ***Stipitipellis*** a thick layer of brown amorphous pigments covers the stipe, with abundant caulocystidia intruding outwards. ***Context trama*** hyaline, smooth, thin-walled and interwoven, similar for pileus and stipe, mostly 22–32 μm, widest at stipe base to 43 μm. ***Clamp connections*** absent.

###### Habitat.

Solitary, associated with *Pinuspumila*.

###### Known distribution.

In northeastern China.

###### Specimens examined.

**China**: • Heilongjiang Province, Da Xing Anling, Xinlin Yuanlin Yuan park (alt. 600 m), 27 August 2010, Xiaofei Shi, Shi 615 (HKAS 63240).

###### Notes.

This species is morphologically and phylogenetically very distinct. Sister species *Suilluspunctipes* does not have such reddish cinnamon cap color and ivory white glandular dots when young.

##### ﻿ Suillus subsibiricus

Taxon classificationAnimaliaBoletalesSuillaceae

R. Zhang, X.F. Shi, G.M. Mueller & P.G. Liu sp. nov.

9672F294-7C04-52B4-8484-84D8C3BC1C3B

822272

Figs 24 , 25

###### Etymology.

This species is related to Siberian *Suillussibiricus*.

**Figure 24.**
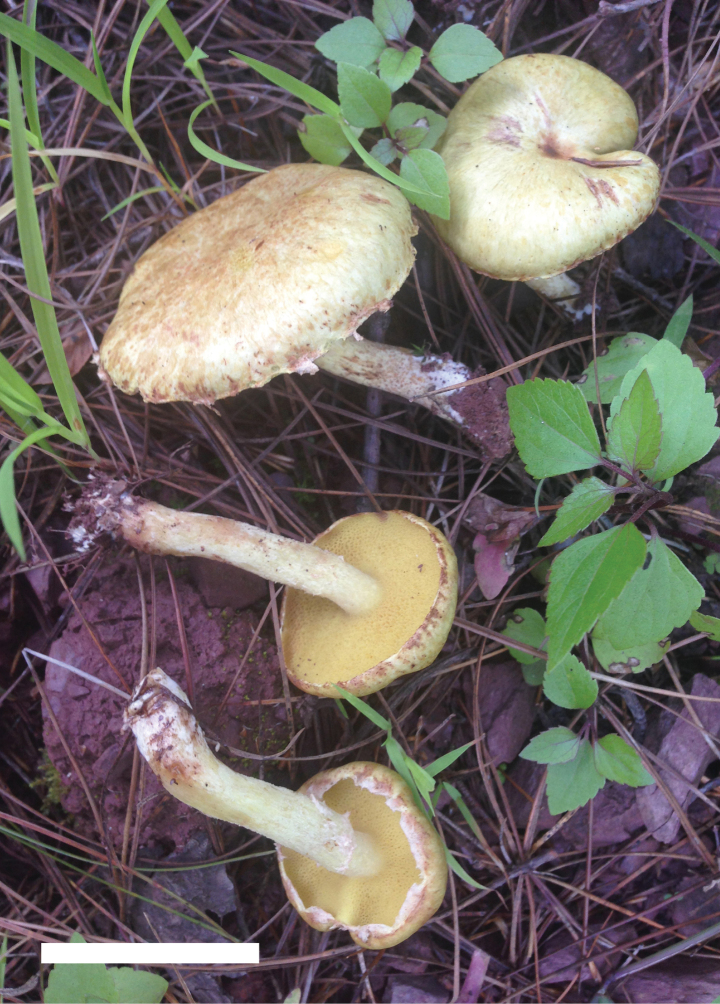
Basidiomata of *Suillussubsibiricus* (holotype, HKAS 91415). Scale bar: 5 cm.

###### Diagnosis.

*Suillussubsibiricus* is in the *S.americanus* morphological complex. This species is in association with *Pinusarmandii* and *P.koraiensis* in southwestern, northeastern and central China.

**Figure 25.**
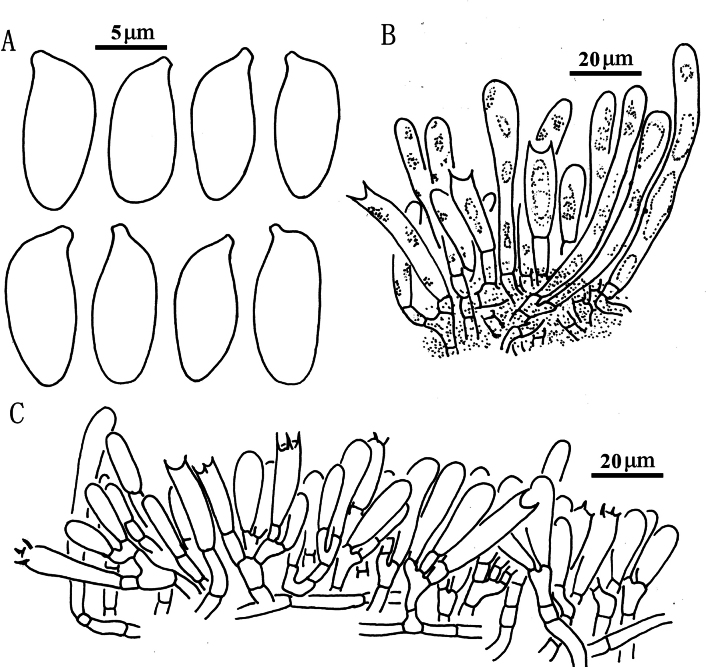
*Suillussubsibiricus* (paratype, HKAS 63155). **a** Basidiospores; **b** Cheilocystidia; **c** Hymenium with basidia. Scale bars: 5 μm (**a**); 20 μm (**b–d**).

###### Typification.

**China**: • Sichuan Province, Huili Town, Da Qing Mountain (27°44'23.23"N, 102°20'24.20"E, alt. 2105 m), 29 July 2012, Rui Zhang, RZ07291202 (holotype, HKAS 91415).

###### GenBank.

ITS = KU663189; LSU = KU721520; *TEFα-1* = KU663197; *RPB1* = KU852259; *RPB2* = KU852349.

###### Morphology.

***Pileus*** convex to flat or slightly umbonate, 2–9 cm diameter, surface viscid, glabrous, background color yellow (3A4, 3A5), covered mainly to the margin with pallid pink (6A2) appressed squamules. Appendiculated with whitish or pinkish (6A3) fibrillose partial veil, lost in age. ***Hymenophore*** subdecurrent to decurrent, yellow (4A4, 4A5), often beaded with pallid yellow (4A2) droplets when young, golden yellow (4A7, 4A8) in age, turning brownish or sometimes light blue when bruised. ***Pores*** 1–2 per mm, round to angular, radially arranged. ***Tubes*** 3–8 mm deep, concolorous with pores, turning brownish or sometimes light blue when cut. ***Stipe*** 3–6.5 × 0.4–1.2 cm, tapering towards apex or equal, solid; cuticle background color yellow (4A3). Glandular dots covering stipe, dense, large and often connected in streaks, yellowish (4A4) when young, becoming reddish brown (6B4, 6B5) or brown (5F6) with age. *Veil* forming appendiculate patches on pileus margin, yellowish white (4A2) and cottony, lost in age. Ephemeral annulus often covering some of the stipe below the annulus, turning pinkish when bruised. ***Mycelia*** white, turning pinkish (6A5, 6A6) when bruised. ***Context*** white to yellowish in pileus and stipe, turning brownish or slightly blue when exposed. ***Spore print*** brown (6D4). ***Odor and taste*** indistinctive.

***Basidiospores*** [80/2/2] 8.5–11 (12) × 3–4 (4.5) μm, Q = 2.22–2.63 (2.75), Q_sd_ = 2.48 ± 0.21, smooth, oblong in face view, narrowly inequilateral with hilar appendage in profile view, hyaline yellow to brown in KOH, tawny brown in Melzer’s. ***Basidia*** 4-spored, clavate, bulbous, 24.0–35.0 × 5.0–7.0 μm, hyaline yellow in KOH, tawny yellow in Melzer’s. ***Hymenophoral trama*** divergent, thin-walled, smooth or wrinkled, sparsely encrusted with granules in KOH, usually 5–8 μm. ***Pleuro- and Cheilocystidia*** usually in fascicles, abundant, clavate or cylindrical, up to 70 μm long, contents brown or hyaline, surrounded by brown amorphous material in KOH. ***Pileipellis*** a layer of ochraceous and scattered gelatinous hyphae, encrusted with fine granules, mostly 4–7 (9) μm wide. ***Stipitipellis*** covered by amorphous pigment, most hyphae thin-walled, septate, 3–10 μm. ***Caulocystidia*** similar to pleuro- and cheilocystidia, embedded by in abundant brown amorphous material. ***Context trama*** hyaline, smooth, thin-walled, interwoven, similar for pileus and stipe, mostly 4–15 μm, up to 30 μm at stipe base. ***Clamp connections*** absent.

###### Habitat.

Solitary to scattered, in association with *Pinusarmandii* and *P.koraiensis*.

###### Known distribution.

Currently known from southwestern, northeastern and central China.

###### Specimens examined.

**China**: • Yunnan Province, Dali City, Miaopu hill (25°34'7.32"N, 100°12'20.87"E, alt. 2208 m), 20 August 2012, Rui Zhang, RZ08201203 (HKAS 91430); **China**: • Yunnan Province, Dali City, Cang Mountain, on the way to the television tower (alt. 2700 m), 22 August 2009, Cai Qing, CaiQing113 (HKAS 58780), **China**: • Heilongjiang Province, Harbin City, Mutan Xian, Dagui town, Friendship village, north side of Menggu Mountain (alt. 200 m), 11 August 2010, Xiaofei Shi, Shi494 (HKAS63155), **China**: • Heilongjiang Province, Yichun City, Liangshui National Nature Reserves (alt. 150 m), 14 August 2010, Xiaofei Shi, Shi521 (HKAS 63156); **China**: • Shaanxi Province, Baoji City, Mei Xian, Yingtou Town, Hao Ping Dali Village (alt. 1300 m), 12 September 2010, Xiaofei Shi, Shi670 (HKAS 63195); **China**: • Shaanxi Province, Ankang City, Langao town, Nangong Mountain (alt. 2000 m), 29 July 2011, Xiaofei Shi, Shi761 (HKAS 71891).

###### Notes.

*Suillussubsibiricus* is morphologically identical to its sister species *S.americanus*. Geographic range and host associations are the keys to differentiate the two species. *Suillusamericanus* was described from North America associated with Pinussubg.strobus and *P.monticola*. Another species, *S.sibiricus*, is not well documented in the literature and lacks holotype and following collections (Singer 1945). *Suillussibiricus* was described from Siberia and presumably is associated with five needle *Pinussibirica*. *Suillushimalayensis*, growing with *P.wallichiana* in Himalayan region, is also in the *S.americanus* complex.

It seems that *S.americanus* and *S.subsibiricus* under current delimitations contain more cryptic species. At least *S.subsibiricus* collections associated with *P.armandii* in southwestern China are not the same as the ones from northeastern China associated with *P.koraiensis*, which is supported by the *TEFα-1*, *RPB1*, *RPB2*, concatenated phylogenies and coalescent analysis (Wu et al. 2000; Mueller et al. 2001). Phylogenies based on the ribosomal regions and morphological characters are not effective in resolving the *S.americanus* complex.

##### ﻿ Suillus pinetorum

Taxon classificationAnimaliaBoletalesSuillaceae

(W.F. Chiu) H. Engel & Klofac, in Engel, et al., Schmier- und Filzröhrlinge s.l. in Europa, Die Gattungen Boletellus, Boletinus, Phylloporus, Suillus, Xerocomus (Weidhausen b. Coburg): 12 (1996)

751F7B99-5127-5817-8D72-9627E666515E

Figs 26 , 27

###### Basionym.

Boletinuspunctatipesvar.pinetorum W.F. Chiu Mycologia, 40(2): 200 (1948)

**Figure 26.**
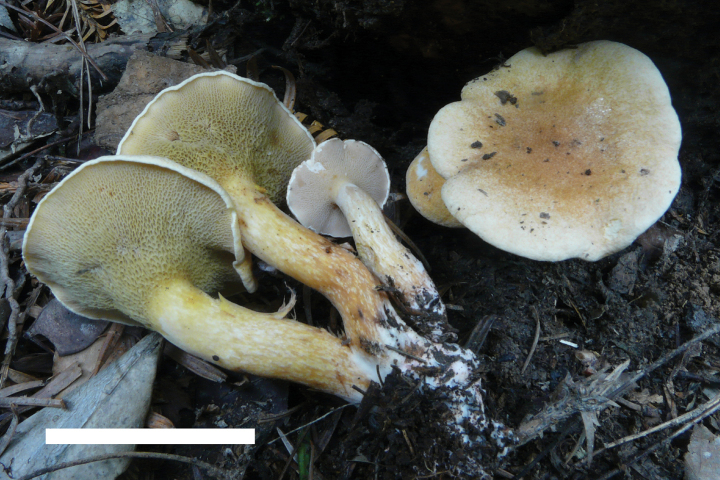
Basidiomata of *Suilluspinetorum* (neotype, HKAS 91427). Scale bar: 5 cm.

###### Holotype.

**China**: • Yunnan Province, Kunming, Tiehfungan, 12 November 1941, W. F. Chiu (no. 7717)

**Figure 27.**
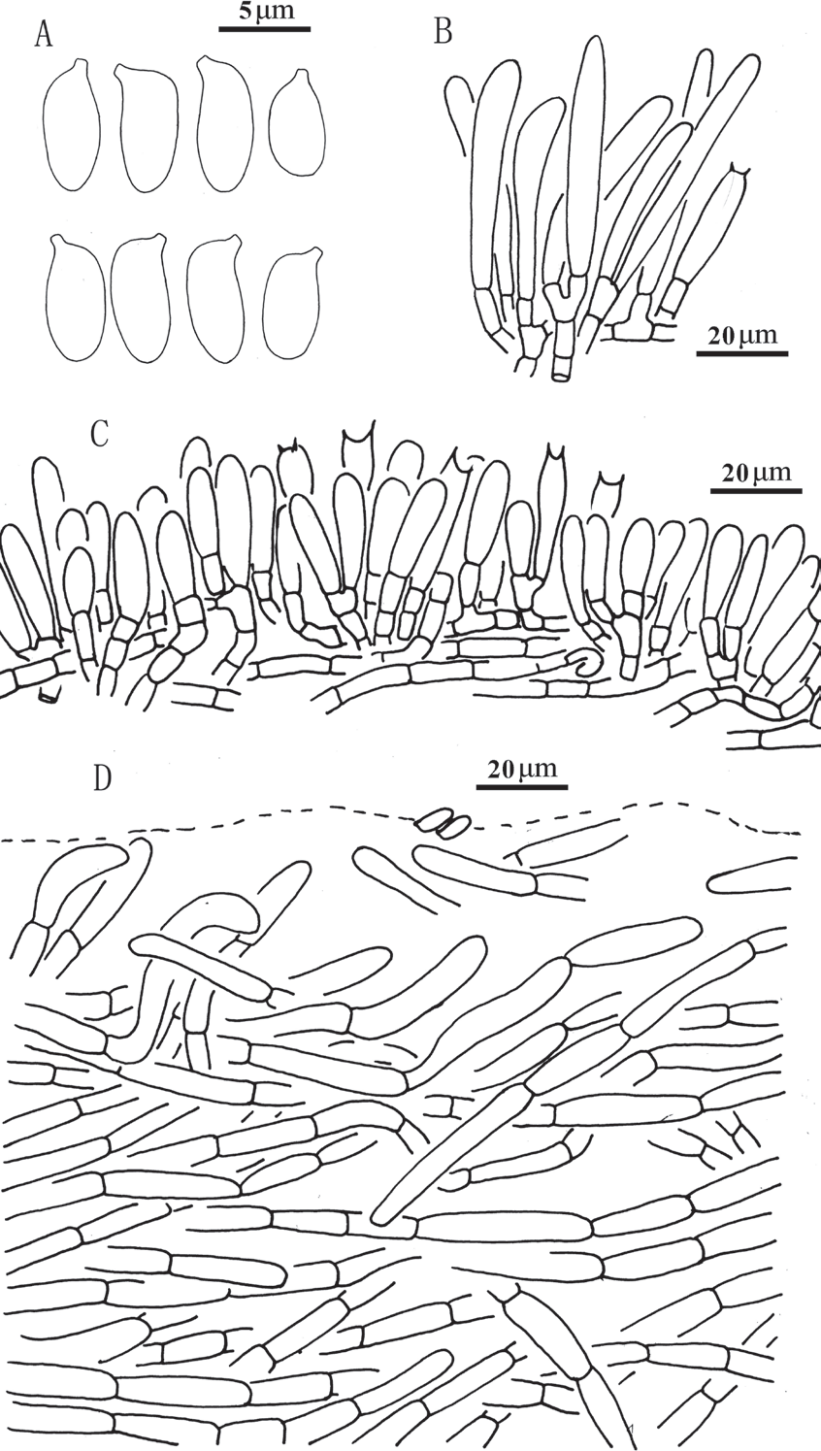
*Suilluspinetorum* (neotype, HKAS 91427). **a** Basidiospores; **b** Cheilocystidia; **c** Hymenium with basidia; **d** Pileipellis (mature specimens). Scale bars: 5 μm (**a**); 20 μm (**b–d**).

###### Diagnosis.

*Suilluspinetorum* is morphologically identical to *S.bovinus*. This species is in association with *Pinusyunnanensis* and *P.massoniana* in southwestern China.

###### Typification.

**China**: • Yunnan Province, Kunming City, Dong Zhan, Qinglong Village (25°03'13.70"N, 102°48'1.50"E, alt. 1942 m), 15 August 2012, Rui Zhang, RZ08151203 (neotype designated here, HKAS 91427, MBT 10025711).

###### Morphology.

***Pileus*** develops from convex to flat, margin inrolled at first, then expanding, 3–5 cm diameter, up to 8 cm broad, surface dry or viscid in moist, glabrous, color beige (5A3) to ochraceous cinnamon (5A6, 5B6). ***Pellicle*** peeling off easily. ***Hymenophore*** subdecurrent to decurrent, younger ones ochraceous (5B4), turn to darker ochraceous (5B5, 5C5) with age. ***Pores*** 1–1.2 mm diameter, angular, compound, radially arranged or inconspicuously lamellate. ***Tubes*** 1–4 mm deep, concolorous with pores, dotted with cinnamon (6A4) colored glandular dots. ***Stipe*** 3–4 × 0.3–1.5 cm, tapering downwards, solid, no glandular dots, no ring, concolorous with pileus, covered with some cinnamon or pinkish (5C6) small streaks. ***Context*** yellowish white to light orange (5A2) in both pileus and stipe, more pinkish (6A2) near cuticles. Do not change color anywhere. ***Mycelia*** white. ***Spore print*** olive brown (4D3). ***Odor and taste*** pleasant, chewy texture.

***Basidiospores*** [80/2/4] (7.0) 7.5–8.5 × 3.0–3.5 μm, Q = (2.00) 2.14–2.67, Q_sd_ = 2.33 ± 0.14, smooth, oblong in face view, narrowly inequilateral with a hilar appendage in profile view, hyaline yellowish in KOH, tawny brown in Melzer’s. ***Basidia*** 4-spored, clavate, bulbous top, 25.0–30.0 × 6.5–8.5 μm, hyaline yellow in KOH. ***Hymenophoral trama*** divergent, all encrusted by fine granules, thin-walled, smooth to wrinkled, mostly 4–8 μm. ***Pleuro- and Cheilocystidia*** in fascicles, abundant, clavate or cylindrical, up to 70 μm long, content brown or hyaline, surrounded by brown amorphous materials in KOH. ***Caulocystidia*** not seen. ***Pileipellis*** an ixocutis, hyphae hyaline, thin-walled and encrusted with hyaline to ochraceous granules, scattered in glue, mostly 3–8 μm, up to 14 μm wide. ***Stipitipellis*** encrusted by patches of amorphous pigments, most hyphae thin-walled, septated, interwoven, up to 13 μm wide. ***Context trama*** hyaline, smooth, thin-walled, interwoven, similar for pileus and stipe, mostly 4–20 μm, widest at stipe base to 40 μm. ***Clamp connections*** absent.

###### Habitat.

Solitary to scattered, in association with *Pinusyunnanensis* and *P.massoniana*.

###### Known distribution.

Mainly distributed in southwestern China.

###### Specimens examined.

**China**: • Yunnan Province, Diqing Tibetan Autonomous Prefecture, Ben Zi Lan, valley of Bai Ma Snow Mountain (alt. 4200 m), 6 September 2009, Xiaofei Shi, Shi260 (HKAS 63132); **China**: • Yunnan Province, Zhanyi Town, Zhu Jiang Yuan, Maxiong Mountain, 30 June 2008, Xiaofei Shi, Shi120 (HKAS 63281); **China**: • Yunnan Province, Dehong Prefecture, Yingjiang, from Tongbi Guan to Xima about 12 km, 18 July 2009, Yanchun Li, Yanchun-Li678 (HKAS 59425); **China**: • Guizhou Province, Zunyi City, from Daozhen to Yangxi about 3 km (alt. 1120 m), 28 July 2010, Xiaofei Shi, Shi393 (HKAS 63200); **China**: • Guizhou Province, Guiyang City, Kaiyang Town, Yonggang village, Gangzhai plantation (alt. 1300 m), 19 June 2010, Xiaofei Shi, Shi348 (HKAS 63136), **China**: • Hunan Province, Zhang Jia Jie City, Zhang Jia Jie National Forest Park, 16 June 2010, Xiaofei Shi, Shi344 (HKAS 63137).

###### Notes.

*Suilluspinetorum* was first documented as “Boletinuspunctatipesvar.pinetorum” by Chiu (1948) and later illustrated by Chiu (1957). The illustration, host and most part of the morphological descriptions match with our collections. However, not as Chiu (1948, 1957) described, the species does not have brown glandular dots and morphological descriptions of this species could be enriched with more details. In addition, the affiliation of *Suilluspinetorum* under *Boletinuspunctatipes* is remotely correct, whereas comparisons with *S.bovinus* were missing. (Engel et al. 1996) reclassified the variety by Chiu (1948) under genus *Suillus* and raised its taxonomic level to species. The type collected by Chiu (1948) is missing at the fungarium of the Institute of Microbiology, Academia Sinica in Beijing, China; so a neotype is assigned here.

*Suilluspinetorum* is morphologically identical with *S.bovinus*. *Suillusbovinus* associates with *Pinussylvestris* and distributes from Europe to northeastern China. *Suilluspinetorum* under current delimitation seems to contain new cryptic species. Collections associated with *Pinusyunnanensis* in southwestern China are not phylogenetically the same as collections growing with *Pinusmassoniana* in southwestern and central China. It is uncertain if host association is rigorous for delimiting cryptic species. *Pinusyunnanensis* and *P.massoniana* are not sister species, and geographic ranges of the two overlap in southwestern China.

##### ﻿ Suillus subg. Boletinus

Taxon classificationAnimaliaBoletalesSuillaceae

(Kalchbr.) R. Zhang, X.F. Shi, G.M. Mueller & P.G. Liu comb. & stat. nov.

749C5ED1-398C-58C2-AE46-0AA1D52B69F4

822254

###### Basionym.

*Boletinus* Kalchbr., Mathematische und naturwissenschaftliche Mitteilungen. Mathem. Természettud. Közlem. 5: 286 (1867).

###### Typification.

*Suilluscavipes* (Klotzsch) A.H. Sm. and Thiers, Monogr. North Amer. Species *Suillus*: 30 (1964)

###### Etymology.

The subgenus name is referred from a former genus name “*Boletinus*”.

###### Diagnosis.

This subgenus differs from all other subgenera of *Suillus* by the presence of clamp connections in the hyphae of sporophores, pileus usually red, bright pinkish or yellowish brown, spore print brown with olive yellow, vinaceous or purple tinge.

###### Morphology.

***Basidiomata*** stipitate-pileate with tubular hymenophore. ***Pileus*** develops from hemispherical to convex or applanate, fibrillose or squamulose, dry, usually red, bright pinkish or yellowish brown when mature; context yellow, no color change when cut. ***Hymenophore*** boletinoid, pores wide and compound, radially arranged, sometimes decurrent. ***Stipe*** subcylindrical to cylindrical, generally hollows in maturity, some remain solid, no glandular dots; veil presents, generally forming a double and dry annulus. ***Spore print*** brown with olive yellow, vinaceous or purple tinge.

***Basidiospores*** smooth, oblong and inequilateral, hyaline yellow in KOH. ***Basidia*** 4-spored, clavate, hyaline yellow in KOH. ***Cystidia*** abundant, large, up to 100 μm, surrounded by brown amorphous materials or not. ***Pileipellis*** a trichoderm, thin-walled, often tangled. *Clamps* constantly present in the trama of sporophores and in basidial basal septum, except in one species.

###### Habitat.

Scattered to gregarious, ectomycorrizal with *Larix*.

###### Known species.

*Suillusampliporus*, *S.asiaticus*, *S.cavipes* complex, *S.ochraceoroseus*, *S.paluster*, and probably *S.foetidus* (See Notes).

###### Notes.

*Boletinus* was delimitated by Singer (1986) as a genus was almost correct except for *S.ochraceoroseus* without clamps. *Boletinus* is better to be retained in the genus *Suillus*. The key feature to differentiate *Boletinus* from other *Suillus* subgenera is the presence of clamps, but this character is not without an exception and the evolutionary function of clamps is not clear. More than three potential new species are reported in subg. Boletinus. *Suilluscavipes* remains a species complex. *Suillusampliporus* is a replaced name of North American *S.cavipes* based on the geographical distribution (Nguyen et al. 2016). The geographical range of *S.cavipes* complex extends to northern China and is likely panboreal.

##### ﻿ Suillus subg. Douglasia

Taxon classificationAnimaliaBoletalesSuillaceae

R. Zhang, X.F. Shi, G.M. Mueller & P.G. Liu subgen. nov.

69350E5D-62DF-514E-9AB5-AB3E57534D65

822257

###### Etymology.

“*Douglasia*” refers from “Douglas fir”, the common name of *Pseudotsugamenziesii*, which is the only known host for the subgenus.

###### Typification.

*Suilluscaerulescens* A.H. Sm. and Thiers

###### Diagnosis.

This subgenus is exclusively associated with *Pseudotsugamenziesii*. Other features of this subgenus include the glabrous or fibrillose pileus; solid, annulated, stipe lacking glandular dots; stipe often changing color to green or blue when cut and lacking clamp connections.

###### Morphology.

***Basidiomata*** stipitate-pileate with tubular hymenophore. ***Pileus*** develops from hemispherical to convex or applanate, glabrous or covered with fine appressed scales, viscid beneath the fibrillose scales, or viscid but not glutinous, rusty to pale brown or with cinnamon tinge; context yellow, no color change when cut. ***Hymenophore*** broadly adnate to subdecurrent, tubes 5–10 mm deep, or up to 15 mm deep, staining brownish when bruised, mouths 1–2.5 mm diameter, angular, and radially arranged. ***Stipe*** equal, tapering downwards or upwards, solid, dry and floccose, lacking glandular dots, veil superior, membranous to floccose or gelatinous; reticulate at apex, context of lower stipe sometimes changing color to green or blue when cut. ***Spore print*** dull cinnamon or brownish.

***Basidiospores*** smooth, oblong or suboblong, ochraceous in KOH, usually 7–11 μm. ***Basidia*** 4-spored, clavate, hyaline yellow in KOH. ***Cystidia*** abundant, typically fasciculate, hyaline, or containing brown content and surrounded by brown amorphous material in KOH. ***Pileipellis*** usually with two layers, inner layer composed of gelatinous interwoven hyphae, and the other of ochraceous smooth or incrusted hyphae in KOH. ***Clamp connections*** absent.

###### Habitat.

Scattered to gregarious, ectomycorrizal with *Pseudotsugamenziesii*.

###### Known species.

*Suilluscaerulescens*, *S.lakei* and *S.ponderosus*

###### Notes.

The grouping of the three species associated with *Pseudotsugamenziesii* (*S.caerulescens*, *S.lakei*, *S.ponderosus*) could not be classified to any subgenera in previous publications. The three species were not treated as related species in Smith and Thiers (1964) or Singer (1986). Previous section classification generally overlooked the host associations with *Pseudotsuga*. The three species were supported as monophyletic in past ITS phylogenies, but no higher classification was proposed (Kretzer et al. 1996; Nguyen et al. 2016). Efforts were undertaken to search for *Suillus* under Asian *Pseudotsuga* trees, but none were found from sporophore surveys or ecological root tip sampling (Murata et al. 2013; Wen et al. 2014; this study). The geographic range of the subgenus is restricted to Western North America and Rocky Mountains in North America delimited by the range of *Pseudotsugamenziesii*.

##### ﻿ Suillus

Taxon classificationAnimaliaBoletalesSuillaceae

subg . Fuscoboletinus (Pomerl. & A.H. Sm.) R. Zhang, X.F. Shi, G.M. Mueller & P.G. Liu comb. & stat. nov.

5E04BA55-4ADB-57D1-8D06-8BC09C448E71

822255

###### Basionym.

*Fuscoboletinus* Pomerl. & A.H. Sm., Brittonia 14: 157 (1962)

###### Typification.

*Fuscoboletinussinuspaulianus* Pomerl. & A.H. Sm.

###### Etymology.

The subgenus name is referred from its type, *Fuscoboletinussinuspaulianus*.

###### Diagnosis.

The subgenus differs from other subgenera mainly by spore print color. Pileus is usually red, and viscid to glutinous. Veils is glutinous, or dry and fibrillose. Glandular dots are lacking. Spore print is purplish brown in mass. Clamp connections are absent and associated with *Larix*.

###### Morphology.

***Basidiomata*** stipitate-pileate with tubular hymenophore. ***Pileus*** develops from hemispherical to convex or applanate, viscid to glutinous, usually red to brown in maturity, with or without scales or fibrils, with appendiculate veil remnants; context yellow, no color change when cut or slowly turning to pinkish and brown. ***Hymenophore*** boletinoid, separable, pores up to 2 mm, angular or irregular, and radially arranged. ***Stipe*** subcylindrical to cylindrical, solid, lacking glandular dots; veil viscid or dry and floccose; stipe surface floccose or viscid. ***Spore print*** purplish brown.

***Basidiospores*** smooth, oblong and inequilateral, hyaline yellow in KOH, usually 7–11 μm, one species has large basidiospores 12–14 μm. ***Basidia*** 4-spored, clavate, hyaline yellow in KOH. ***Cystidia*** abundant, typically fasciculate, large, up to 100 μm, with brown content and surrounded by brown amorphous materials in KOH. ***Pileipellis*** a layer of gelatinous hyphae, with yellowish hyaline content in KOH. ***Clamp connections*** absent.

###### Habitat.

Scattered to gregarious, ectomycorrizal with *Larix*.

###### Known species.

*Suillusglandulosus*, *S.sinuspaulianus* and *S.spectabilis*.

###### Notes.

The grouping of *S.spectabilis*, *S.sinuspaulianus*, and *S.glandulosus* was not found in older mycological references. Singer (1986) placed *Suillusspectabilis* and *S.ochraceoroseus* in section Solidipedes, and *S.sinuspaulianus* and *S.glandulosus* in section Glandulosi. Pomerleau and Smith (1962) classified all three species in the genus *Fuscoboletinus* along with several species from other *Suillus* subgenera (Table 1). Subgenus Fuscoboletinus is the less speciose subgenus in genus Suillus.

Further studies are required for identifying hosts of *S.sinuspaulianus* and *S.glandulosus* and for determining whether the two are better treated as synonyms (Nguyen et al. 2016). Reported hosts of *S.sinuspaulianus* are *Pinus* sp., *Abiesbalsamea* and *Piceaglauca* in Canada (Pomerleau and Smith 1962). Documented hosts of *S.glandulosus* are *Abiesbalsamea* in Quebec (Canada) and *Thujaoccidentalis* in Michigan (USA) (Pomerleau and Smith 1962). The hosts of this species have never been confirmed from colonized mycorrhizal root tips, but phylogeny is well-supported to place this species within the *Larix* associated *Suillus* clade. Therefore, Nguyen et al. (2016) suggests that *Larix* species could be the primary host. However Pérez-Pazos et al. (2021) showed that *Picea* and *Abies* can also be secondary hosts. However, none of the hosts have been adequately confirmed. Collections of *Suillusspectabilis* from northeastern China were supported as conspecific with North American counterparts by GCPSR and coalescence. Population genetic studies are needed to assess the genomic divergence of *S.spectabilis* populations across the continents.

##### ﻿ Suillus subg. Larigni

Taxon classificationAnimaliaBoletalesSuillaceae

(Singer ex Estadès and Lannoy) R. Zhang, X.F. Shi, G.M. Mueller & P.G. Liu comb. & stat. nov.

0E97E324-0A44-5927-8F63-CE96CC0B67BB

822256

###### Basionym.

Suillussect.Larigni Singer ex Estadès and Lannoy Docums Mycol. 31(no. 121): 57 (2001).

###### Etymology.

was derived from “*Larignus*”, meaning of the larch.

###### Typification.

*Suillusgrevillei* (Klotzsch) Singer, Farlowia 2(2): 259 (1945).

###### Diagnosis.

Main features of this subgenus include glabrous and glutinous or dry pileus with scales, solid, annulate stipe, lacking glandular dots, olive brown or dull cinnamon spore print and lacking clamp connections.

###### Morphology.

***Basidiomata*** stipitate-pileate with tubular hymenophore. ***Pileus*** develops from hemispherical to convex or applanate, viscid to glutinous and glabrous, or dry with appressed scales, usually yellowish, orange or brown in maturity, sometimes pellicle separable, context yellow, no color change when cut. ***Hymenophore*** adnate to subdecurrent, 1 to 2 per mm, angular. ***Stipe*** equal to slightly clavate, solid, lacking glandular dots, veil dry and floccose, in some species context changes color to blue or green when exposed. ***Spore print*** olive brown to brown when moist, dull cinnamon when dry.

***Basidiospores*** smooth, oblong or suboblong, ochraceous in KOH, usually 7–11 μm. ***Basidia*** 4-spored, clavate, hyaline yellow in KOH. ***Cystidia*** abundant, typically fasciculate, hyaline, or containing brown contents and surrounded by brown amorphous materials in KOH. ***Pileipellis*** a layer of gelatinous hyphae, with yellowish hyaline content in KOH. ***Clamp connections*** absent.

###### Habitat.

Scattered to gregarious, ectomycorrizal with *Larix*.

###### Known species.

*Suillusalpinus*, *S.aurihymenius*, *S.bresadolae*, *S.elbensis*, the *S.grevillei* complex, *S.grisellus*, *S.lariciphilus*, *S.phylolaricinus*, *S.tridentinus* and *S.viscidus* complex.

###### Notes.

Excluding subgenera *Boletinus* and *Fuscoboletinus*, the rest of the *Suillus*–Larix species belong to subgenus Larigni. Section Larigni (Singer 1986) is very close to the current delimitation of subgenus Larigni because representative species were included from all major clades in this section. But the taxonomic description of section Larigni was not comprehensive and the known diversity was not comparable with the current understanding.

Many new species in the subg. Larigni can be recognized and supported by molecular phylogenies. However, taxonomic treatments applying for the new species are deferred pending further study. More focused study is required for resolving the *S.grevillei* and *S.viscidus* complexes with minor morphological variations. More comprehensive collections help to confirm host associations and geographic range of some cryptic new species. Though geographic range seems useful in resolving species in the two complexes, one putative new species of *S.grevillei* distributes intercontinentally across North America and Europe. East Asia is the diversity hotspot for subgenus Larigni. East Asian collections are found in all major clades of subg. Larigni. Efforts towards the sampling of East Asian *Larix* have resulted in the discovery of many new species in the subgenus (Shi et al. 2016). For further studies on the subg. Larigni, it is recommended to use LSU, *TEFα-1* and *RPB2* instead of ITS sequences to resolve phylogenetic relationships.

##### ﻿ Suillus phylolaricinus

Taxon classificationAnimaliaBoletalesSuillaceae

R. Zhang, X.F. Shi, G.M. Mueller & P.G. Liu sp. nov.

B49246EF-F0E2-5C73-ACA0-E6A07AC396EE

822261

Figs 28 , 29

###### Etymology.

“*Laricinus*” indicates that the host of this species is *Larix*. “*Phylo*-” means this species is first discovered and confirmed by molecular phylogeny.

**Figure 28.**
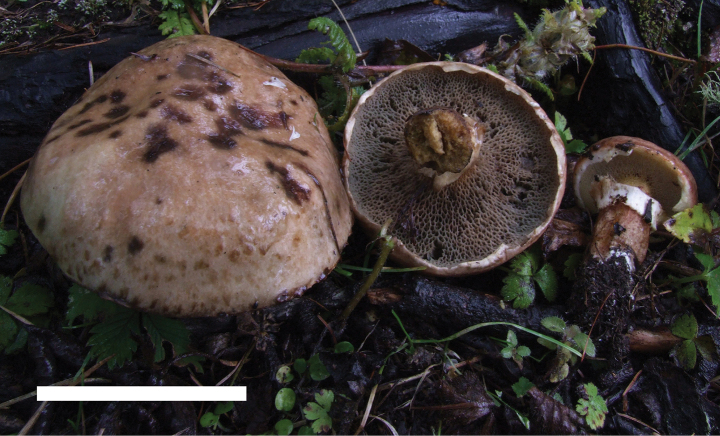
Basidiomata of *Suillusphylolaricinus* (holotype, HKAS 63176). Scale bar: 5 cm.

###### Typification.

**China**: • Yunnan Province, Diqing Tibetan Autonomous Prefecture, Shangri-la, near Tian-chi (27°34'42.45"N, 99°45'58.92"E, alt. 3321 m), 17 September 2010, Xiaofei Shi, Shi696 (holotype HKAS 63176).

**Figure 29.**
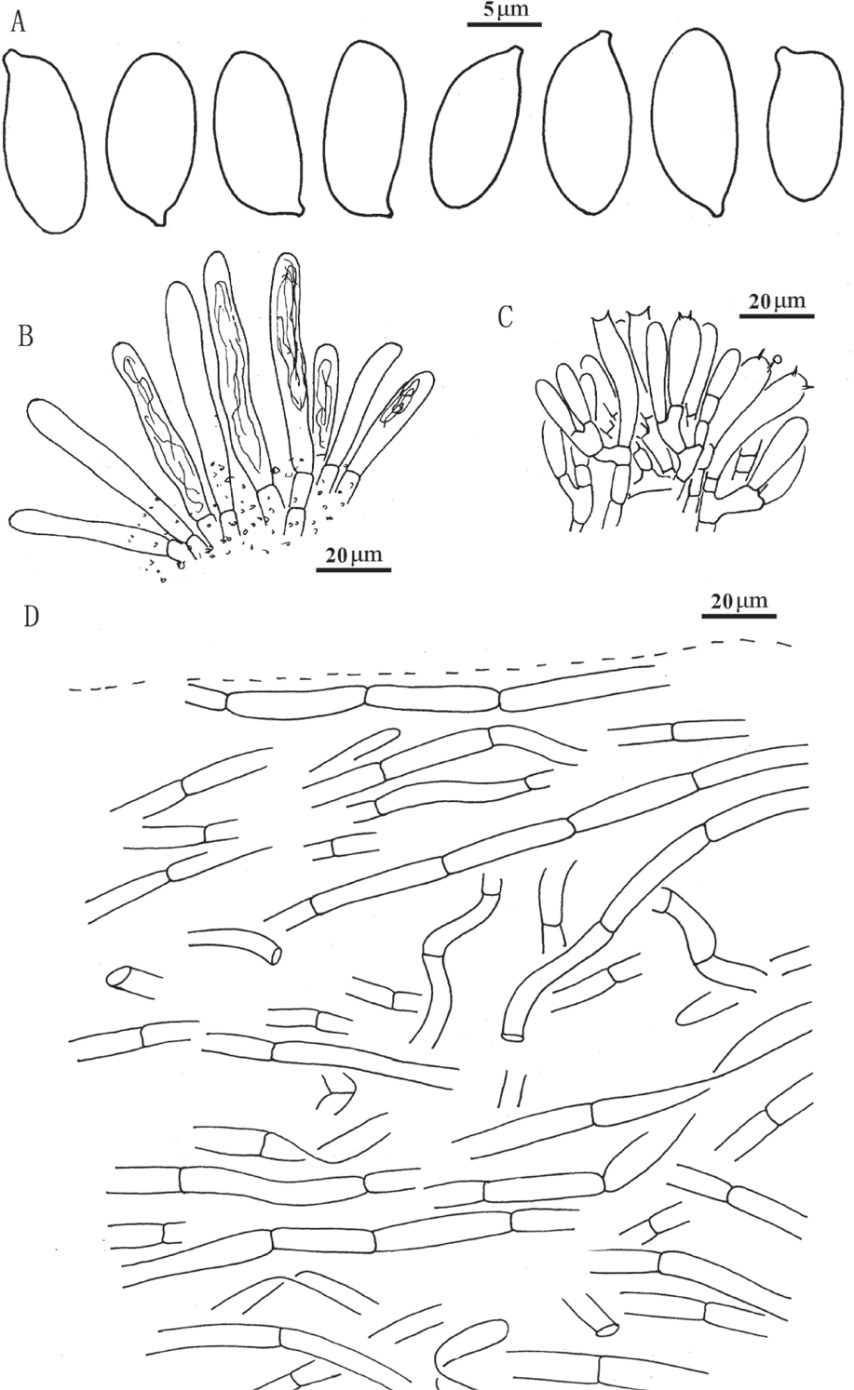
*Suillusphylolaricinus* (holotype, HKAS 63176). **a** Basidiospores; **b** Cheilocystidia; **c** Hymenium with basidia; **d** Pileipellis (mature specimens). Scale bar: 5 μm (**a**); 20 μm (**b–d**).

###### Diagnosis.

The distinctive feature of *S.phylolaricinus* is the color change of the context at the stipe apex. When cut, the context of the upper stipe turns to greenish blue immediately.

###### GenBank.

ITS = KU721438; LSU = KU663211; *TEFα-1* = KU721693; *RPB1* = KU852243; *RPB2* = KU852332.

###### Morphology.

***Pileus*** develops from convex to applanate, 3–10 cm diameter, surface viscid to glutinous, glabrous, some appressed brown scales (7D3–7D5) underneath the glue, denser in cap margin, cap usually a mixture of color patches from pale orange (6A2, 6A3) to orange brown (6B3, 6C3), younger sporophores brownish gray with olive tinge (4C2, 4B3). ***Pellicle*** is easily separable. Margin appendiculated with veil remnants and incurved slightly. ***Hymenophore*** subdecurrent to decurrent, younger ones yellowish (3A3, 3A4), turning to brownish or orange gray with age (6B2, 6B3, 6C2), changing to darker brownish gray when bruised. ***Pores*** 1–2 per mm, angular, compound. ***Tubes*** 4–7 mm deep. ***Stipe*** 4–10 × 0.6–2 cm, equal to slightly clavate, solid, lacking glandular dots, veil superior dry and floccose, color white turning blackish brown with age, reticulate above the annulus. ***Context*** white to yellowish (3A2, 3A3) in pileus and stipe, more yellowish at stipe apex, upper part of the stipe turning greenish blue (25A4, 26A3) when exposed, and stipe base more orange color (5A4). ***Spore print*** dark brown (6F3) when moist, dull cinnamon (6E6, 6D5) when dry. ***Odor and taste*** indistinctive.

***Basidiospores*** [80/2/2] 7.8–11.5 × 5.0–5.5 μm, Q = (1.90) 2.00–2.30, Q_sd_ = 2.13 ± 0.11, smooth, oblong in face view, narrowly inequilateral with a hilar appendage in profile view, hyaline yellow or brown in KOH. ***Basidia*** 2- to 4-spored, clavate, bulbous top, 17.0–32.0 × 5.0–10.0 μm, hyaline yellow in KOH. ***Hymenophoral trama*** not wrinkled, smooth, more or less interwoven, most 3–7 μm, up to 14 μm, thin-walled, hyaline. ***Pleuro- and Cheilocystidia*** in fascicles, abundant, clavate or cylindrical, up to 100 μm long, content brown or hyaline, surrounded by brown amorphous materials in KOH. ***Caulocystidia*** not seen. ***Pileipellis*** a layer of scales on top, hyphae smooth, content ochraceous, mostly 3–8 μm, up to 10 μm. Encrusted hyphae with hyaline granules, 3–8 μm underneath a gelatinous layer. ***Stipitipellis*** a somewhat gelatinous layer, encrusted with ochraceous amorphous materials. ***Context trama*** hyaline, smooth, thin-walled, interwoven, mostly 6–30 μm, up to 60 μm. ***Clamp connections*** absent.

###### Habitat.

Solitary to scattered, in high elevation forests of *Larixpotaninii*.

###### Known distribution.

Currently only known from subalpine region in southwestern China.

###### Specimens examined.

**China**: • Yunnan Province, Diqing Tibetan Autonomous Prefecture, Ben Zi Lan, valley of Bai Ma Snow Mountain (alt. 4200 m), 6 September 2009, Xiaofei Shi, Shi260 (HKAS 63132); *ibid* 12 October 2011, Xiaofei Shi, Shi1024 (HKAS 71997); **China**: • Yunnan Province, Diqing Tibetan Autonomous Prefecture, Shangri-la, near Napa (27°52'48.61"N, 99°37'04.03"E, alt. 3500 m), 11 October 2011, Xiaofei Shi, Shi 1022 (HKAS 71995); **China**: • Yunnan Province, Diqing Tibetan Autonomous Prefecture, Deqin Town, 18 August 2008, Yan-chun Li, Yan-chun 1518 (HKAS 56358); **China**: • Tibetan Autonomous Prefecture, by the road about 24 km away from Bomi to Motuo county, 22 June 2009, Bang Feng, Bang-Feng 366 (HKAS 57095).

###### Notes.

*Suillusphylolaricinus*, *S.grisellus* and *S.lariciphilus* have appressed scales, which is a distinct morphological character in subg. Larigni. The host is another key to identify these species. Hosts of *S.lariciphilus* are *Larixgriffithiana* and *L.himalaica*, while *Larixlaricina* is the host for *S.grisellus* (Pomerleau and Smith 1962; Adamčík et al. 2015). *Suillusgrisellus* reported from China for the first time associated with *Larixlaricina*.

## ﻿Discussion

This study is a comprehensive study that provides a modern update of subgeneric classification of *Suillus*, supported by multi-gene phylogeny. Multigene phylogenies enable the first application of GCPSR, concatenation and coalescent analyses of *Suillus* for higher classification and delimiting species. The monophyly of *Suillus* was supported by molecular phylogenies of ITS, *RPB1*, concatenated datasets and the coalescent analysis (Table 3). Further analyses are necessary to confirm the sister relationship of *Suillus* and the gasteroid *Truncocolumella* (Binder and Hibbett 2006). *Truncocolumellacitrina* was included in the ribosomal DNA phylogenies, but protein-coding genes should be acquired and analyzed for this genus (Suppl. material 1: figs S2, S3). The higher-level taxonomic conflicts of *Suillus* among previous classifications (Suppl. material 1: table S1) based mainly on morphology are resolved. Subgenera are recognized for the first time in the *Suillus* taxonomy. Host association and some morphological features such as the presence or absence of clamp connections are confirmed as the diagnostic features. Concatenation analyses were more effective than coalescence in supporting subgenera (Table 3). The performance of coalescence analysis in resolving subgeneric relationships would likely be improved with the inclusion of a larger number of single gene phylogenies (Mirarab and Warnow 2015).

Previously unrecognized *Suillus* diversity in East Asia was revealed in this study, and a large number of new species were identified through the intensive fieldwork and the phylogenetic analyses. Some of these new species have distinct morphological and ecological features. Morphological features alone are not effective in differentiating global *Suillus* species. For instance, *Suillusdecipiens*, *S.kwangtungensis*, *S.phylopictus* and *S.spraguei* resemble each other (Zhang et al. 2017, 2022). The current standard GCPSR approach for identifying fungal species addresses incomplete lineage sorting (ILS) by comparing discrepancies among gene trees for a given taxonomic group (Taylor et al. 2000). An alternative approach, coalescent analysis, is functionally similar to GCPSR. It automatically estimates the species tree from multiple gene trees to resolve the problems in ILS (Mirarab et al. 2014; Mirarab and Warnow 2015; Singh et al. 2015) and has been applied to estimating fungal species trees from multiple phylogenies of different loci (Leavitt et al. 2012; Liu et al. 2016). With the coalescent phylogeny, individual phylogenetic trees from different loci are no longer required, eliminating the need for manual interpretations of the GCPSR standard.

Challenges with species recognition by single gene phylogenies have been highlighted in recent years (Schoch et al. 2012; Hibbett and Taylor 2013; Kõljalg et al. 2013). Even so, before this study, newly described *Suillus* species were primarily identified only on the phylogenies of ITS sequences (Bruns et al. 2010; Sarwar et al. 2015). Some species complexes are not resolved in the ITS phylogeny (Suppl. material 1: fig. S2). Since the ITS phylogeny was used as the backbone to select samples for amplifying the other genes, it is unknown if such practice reduced the number of cryptic *Suillus* species being resolved (e.g., in the *S.grevillei* complex). Cryptic species in *Suillus* species complexes can often be delimited by host association or geographic distribution, e.g., occurring in different continents. Broadly distributed species such as *Suillusamericanus*, *S.ampliporus*, *S.flavopunctipes* and *S.spectabilis*, may contain cryptic species resolvable through population genetic or genomic studies.

This study has documented the need for multiple molecular datasets to resolve cryptic species, for acquiring observations covering all developmental stages of the potential new species, for sampling comprehensively to cover the geographic range, and for identifying host associations supplemented by ecological studies or in vitro synthesis. *Suillus* species are comparatively well-sampled in this study. However, sampling gaps still exist in some understudied regions and in association with unreported hosts. In North Asia, *Pinuspumila*, *P.sibirica* and *Larixsibirica* occupy large geographic ranges and may contain undocumented species. Other understudied hosts include *Pinusbrutia*, *P.halepensis* and *P.heldreichii* in Mediterranean regions; *Pinusdalatensis*, *P.fenzeliana*, *P.kesiya*, *P.krempfii* and *P.latteri* in Southeast Asia; *P.luchuensis*, *P.morrisonicola*, *P.taiwanensis* and *Larixkaempferi* in Taiwan island and Japan; and *Pinuscanariensis* in the Canary Islands off the northwest coast of Africa.

In Table 1, we summarized the genera, subgenera and sections classified by previous studies. However, the subsections were not emphasized because there is no phylogenetic support in the phylogenetic trees. It is important that any section level morphological classifications are supported by phylogenetic evidence before they are adopted. Rigorous subsection classification requires more sample collections and molecular sequences. We are not confident about confirming the subsections delimited by Singer (1986) and Estadès and Lannoy (2001), especially for SuillussubgenusLarigni. As shown in Fig. 1 and Fig. 3, dividing this subgenus into subsections would require more in depth phylogenetic resolution including more samples, more molecular sequences and greater geographic coverage.

## Supplementary Material

XML Treatment for Suillus

XML Treatment for Suillus Gray subg. Suillus

XML Treatment for Suillus Gray sect. Suillus

XML Treatment for Suillus aenoplacidus

XML Treatment for Suillus flavopunctipes

XML Treatment for Suillus longiflavopunctipes

XML Treatment for Suillus aestivoluteus

XML Treatment for Suillus zangii

XML Treatment for Suillus sect. Diversipedes

XML Treatment for Suillus boletoluteus

XML Treatment for Suillus cinerescens

XML Treatment for Suillus minusculus

XML Treatment for Suillus phylosubaureus

XML Treatment for Suillus subcinnamomeus

XML Treatment for Suillus subsibiricus

XML Treatment for Suillus pinetorum

XML Treatment for Suillus subg. Boletinus

XML Treatment for Suillus subg. Douglasia

XML Treatment for Suillus

XML Treatment for Suillus subg. Larigni

XML Treatment for Suillus phylolaricinus

## ﻿Additional information

### Use of AI

No use of AI was reported.

### Adherence to national and international regulations

All the fungal strains used in this study have been legally obtained, respecting the Convention on Biological Diversity (Rio Convention).

### Author ORCIDs

Xiaofei Shi https://orcid.org/0000-0001-5752-0574

Gregory M. Mueller https://orcid.org/0000-0001-8316-9319

Peigui Liu https://orcid.org/0000-0002-4405-7899

Fuqiang Yu https://orcid.org/0000-0001-7931-080X

Indunil C. Senanayake https://orcid.org/0000-0002-7165-2394

### Data availability

All of the data that support the findings of this study are available in the main text or Supplementary Information.

## Supplementary materials

Supplementary material 1 Phylogenetic analysis, sequences accession numbers and collection details of samples Data type docx https://binary.pensoft.net/file/1376323This dataset is made available under the Open Database License (http://opendatacommons.org/licenses/odbl/1.0/). The Open Database License (ODbL) is a license agreement intended to allow users to freely share, modify, and use this Dataset while maintaining this same freedom for others, provided that the original source and author(s) are credited.Xiaofei Sh
