# Supplementary material for: ﻿A subgeneric revision of the genus Suillus (Suillaceae, Boletales) and novel taxa from Eastern Asia based on morphology and multigene phylogenies
Source: IMA Fungus. 2025 Jul 17;16:e144260. doi: 10.3897/imafungus.16.144260 (PMC12290465; doi:10.3897/imafungus.16.144260)
Supplement: Supplementary material 1 — Phylogenetic analysis, sequences accession numbers and collection details of samples [file imafungus-16-e144260-s001.docx]

# Supplementary data

# Table S1 *Suillus* species counted in single gene phylogenies, concatenated phylogeny of five loci, GCPSR and coalescence analysis. Number of OTUs is counted from each phylogeny from figure 1 & 2, and supplementary figure 1 to 5. Red letters are for new species blue for new type collections (neotype).

| **Species names** | **ITS** | **LSU** | ***TEFα-1*** | ***RPB1*** | ***RPB2*** | **GCPSR** | **Concatenation** | **Coalescence** | **Comments** |
| --- | --- | --- | --- | --- | --- | --- | --- | --- | --- |
| *S. aenoplacidus* | 1 | 1 | 1 | 1 | 1 | 1 | 1 | 1 |  |
| *S. aestivoluteus* | 1 | 1 | 1 | 1 | 1 | 1 | 1 | 1 |  |
| *S. acidus-S. subolivaceus* | 1 | 1 | 1 | 1 | 1 | 1 | 1 | 1 |  |
| *S. alpinus* | 1 | 2 | 1 | 1 | 1 | 1 | 1 | 1 |  |
| *S. americanus* | 2 | 2 | 1 | 1 (syn. *S. subsibiricus*) | 1 | 1 | 2 | 1 |  |
| *S. ampliporus* | 2 | 1 | 1 | 1 | 1 | 1 | 1 | 1 | Multigenes needed, might contain new species |
| *S. anomalus* | 1 |  |  |  |  |  |  |  | Multigenes needed |
| *S. asiaticus* | 1 | 1 | 2 | 1 | 1 | 1 | 1 | 1 |  |
| *S. aurihymenius* | 1 | 1 | 1 | 1 | 1 | 1 | 1 | 1 |  |
| *S. bellinii* | 1 | 1 | 1 | 1 | 1 | 1 | 1 | 1 |  |
| *S. boletoluteus* | 1 | 1 | 1 | 1 | 1 | 1 | 1 | 1 |  |
| *S. borealis-S. brunnescens* | 1 |  |  |  |  |  |  |  | Multigenes needed |
| *S. bovinus* | 1 | 1 | 1 (syn. *S. pinetorum*) | 1 (syn. *S. pinetorum*) | 1 | 1 | 2 | 2 | More samples needed, might contain new species |
| *S. bresadolae* | 1 | 1 | 1 |  |  | 1 | 1 | 1 |  |
| *S. brevipes* | 1 | 1 | 1 | 1 | 1 | 1 | 1 | 1 |  |
| *S. caerulescens* | 1 | 1 | 1 | 1 | 1 | 1 | 1 | 1 |  |
| *S. cavipes* complex | 3 | 2 | 3 |  |  | 2 | 2 | 2 | Contains undescribed species, more samples needed |
| *S. cinerescens*  complex | 2 | 2 (includes *S.*  *plorans*) | 2 | 2 |  | 3 | 3 | 3 | Contains cryptic species, more samples are needed |
| *S. collinitus* | 1 |  |  |  |  |  |  |  | Multigenes needed |
| *S. decipiens* | 1 | 1 | 1 |  | 1 | 1 | 1 | 1 |  |
| *S. discolor* | 1 |  |  |  |  |  |  |  | Multigenes needed |
| *S. elbensis* | 1 | 1 | 1 | 1 (syn. *S.*  *grisellus*) | 1 | 1 | 1 | 1 |  |
| *S. flavidus* - *S. helenae - S. umbonatus* | 1 | 1 | 1 (syn. S. megaporinus) | 1 | 1 | 1 | 1 | 1 |  |
| *S. flavopunctipes* | 1 | 1 | 1 | 2 | 1 (syn. *S. longiflavipun-*  *ctipes*) | 1 | 1 | 1 |  |
| *S. fluryi* complex | 3 | 2 | 1 |  | 1 | 2 | 2 | 1 | Contains undescribed species, more samples needed |
| *S. foetidus* | 1 |  |  |  |  |  |  |  | Multigenes needed |
| *S. fuscotomentosus* - *S. variegatus - S.* *acerbus* | 1 | 1 | 1 | 1 | 1 | 1 | 1 | 1 |  |
| *S. glandulosipes* | 1 | 1 | 1 | 1 | 1 | 1 | 1 | 1 |  |
| *S. granulatus* | 1 | 1 | 1 |  | 1 | 1 | 1 | 1 |  |
| *S. grevillei* complex | 3 | 6 | 6 | 2 | 5 | 5 | 5 | 4 | Contains undescribed species, more samples needed |
| *S. grisellus* | 1 | 1 | 1 | 1 (syn *S. elbensis*) | 1 | 1 | 1 | 1 |  |
| *S. hirtellus* | 2 | 1 | 1 |  | 1 | 1 | 1 | 1 |  |
| *S. kaibabensis* | 1 |  |  |  |  |  |  |  | Multigenes needed |
| *S. kwangtungensis* | 1 | 1 | 1 | 1 |  | 1 | 1 | 1 |  |
| *S. lakei* | 1 | 1 | 1 | 1 | 1 | 1 | 1 | 1 |  |
| *S. lariciphilus* | 1 |  |  |  |  |  |  |  | Multigenes needed |
| *S. longiflavopunctipes* | 1 | 1 | 1 | 1 | 1 (syn. *S.*  *flavipunctipe*) | 1 | 1 | 1 |  |
| *S. luteus* | 1 | 1 | 1 | 1 | 1 | 1 | 2 | 1 |  |
| *S. marginielevatus - S.indicus* | 1 |  |  |  |  |  |  |  | Multigenes needed |
| *S. mediterraneensis* | 1 | 1 | 1 |  | 1 | 1 | 1 | 1 |  |
| *S. megaporinus* | 1 | 1 | 1 (syn. *S. flavidus*) |  |  | 1 | 1 | 1 |  |
| *S. minusulus* | 1 | 1 | 1 | 1 | 1 | 1 | 1 | 1 |  |
| *S. neoalbidipes* | 1 |  |  |  |  |  |  |  | Multigenes needed |
| *S. occidentalis* | 1 |  |  |  |  |  |  |  | Multigenes needed |
| *S. ochraceoroseus* | 1 | 1 (syn. *S. paluster*) | 1 (syn. *S. paluster*) |  | 1 | 1 | 1 (syn. *S. paluster*) | 1 | More samples and multigenes needed |
| *S. paluster* | 1 | 1 (syn. *S. ochraceoro- seus*) | 1 (syn. *S. ochraceoro- seus*) | 1 |  | 1 | 2 (syn. *S. ochraceoro- seus*) | 2 | More samples and multigenes needed |
| *S. phylolaricinus* | 1 | 1 | 1 | 1 | 1 | 1 | 1 | 1 |  |
| *S. phylopictus* | 2 | 2 | 2 | 2 | 1 | 2 | 2 | 2 | Contains undescribed species, more samples needed |
| *S. phylosubaureus* | 1 | 1 (syn. *S. subaureus*) | 1 |  |  | 1 | 1 | 1 |  |
| *S. pinetorum* | 2 | 2 | 2 (syn. *S.*  *bovinus*) | 1 (syn. *S.*  *bovinus*) | 1 | 2 | 2 | 2 | Contains cryptic species, more samples are needed |
| *S. placidus* | 1 | 1 | 1 | 1 | 1 | 1 | 1 | 1 |  |
| *S. plorans* | 2 | 2 (syn. *S. cinerescens*) | 2 |  |  | 2 | 2 | 2 | Contain geographic cryptic species. |
| *S. ponderosus* | 1 | 1 | 1 | 1 | 1 | 1 | 1 | 1 |  |
| *S. pseudobrevipes -S. wasatchicus-S.* | 1 | 1 | 1 | 1 | 1 | 1 | 1 | 1 |  |
| *S.volcanalis-S. albivelatus-S. cf pseudogranulatus-S.*  *pseudoalbivelatus* |  |  |  |  |  |  |  |  |  |
| *S. punctipes* | 1 |  |  |  |  |  |  |  | Multigenes needed |
| *S. pungens* | 1 | 1 | 1 | 1 | 1 | 1 | 1 | 1 |  |
| *S. quiescens* | 1 | 1 | 1 | 1 | 1 | 1 | 1 | 1 |  |
| *S. salmonicolor -S. cothurnatus* | 2 | 1 | 1 |  | 1 | 1 | 1 | 1 |  |
| *S. sinuspaulianus* -*S.*  *glandulosus* | 1 | 1 | 1 | 1 | 1 | 1 | 1 | 1 |  |
| *Suillus* sp. (Colorado USA, CO37) | 1 |  |  |  |  |  |  |  | Multigenes needed |
| *Suillus* sp. (Japan, GifuB2) | 1 |  |  |  |  |  |  |  | Multigenes needed |
| *Suillus* sp. (Russia, clone 250) | 1 |  |  |  |  |  |  |  | Multigenes needed |
| *S. spectabilis* | 1 | 1 | 1 | 1 | 2 | 1 | 1 | 1 |  |
| *S. spraguei* | 1 | 1 | 1 | 1 | 1 | 1 | 1 | 1 |  |
| *S. subalpinus* | 1 |  |  |  |  |  |  |  | Multigenes needed |
| *S. subalutaceus* | 1 |  |  |  |  |  |  |  | Multigenes needed |
| *S. subaureus* | 1 | 1 | 1 | 1 | 1 | 1 | 1 | 1 |  |
| *S. subcinnamomeus* | 1 |  |  |  |  |  | 1 | 1 | Multigenes needed |
| *S. subsibiricus* | 1 | 1 | 2 | 2 (syn. *S. americanus*) | 2 | 2 | 2 | 2 | Contains cryptic species, more samples are needed |
| *S. suilloides-S. amaranthii-S. umbrinus* | 1 |  |  |  |  |  |  |  | Multigenes needed |
| *S. tomentosus* complex | 2 | 1 | 1 | 1 | 2 | 2 | 2 | 2 | Contains undescribed species, more samples needed |
| *S. triacicularis* | 1 |  |  |  |  |  |  |  | Multigenes needed |
| *S. tridentinus* | 1 | 1 | 1 | 1 | 1 | 1 | 1 | 1 |  |
| *S. viscidus-S. subolivaceus* complex | 3 | 3 | 2 | 2 | 2 | 2 | 3 | 2 | Contains undescribed species, more samples needed |
| *S. weaverae* | 1 |  |  |  |  |  | 1 | 1 | Multigenes needed |
| *S. zangii* | 1 | 1 | 1 | 1 | 1 | 1 | 1 | 1 |  |


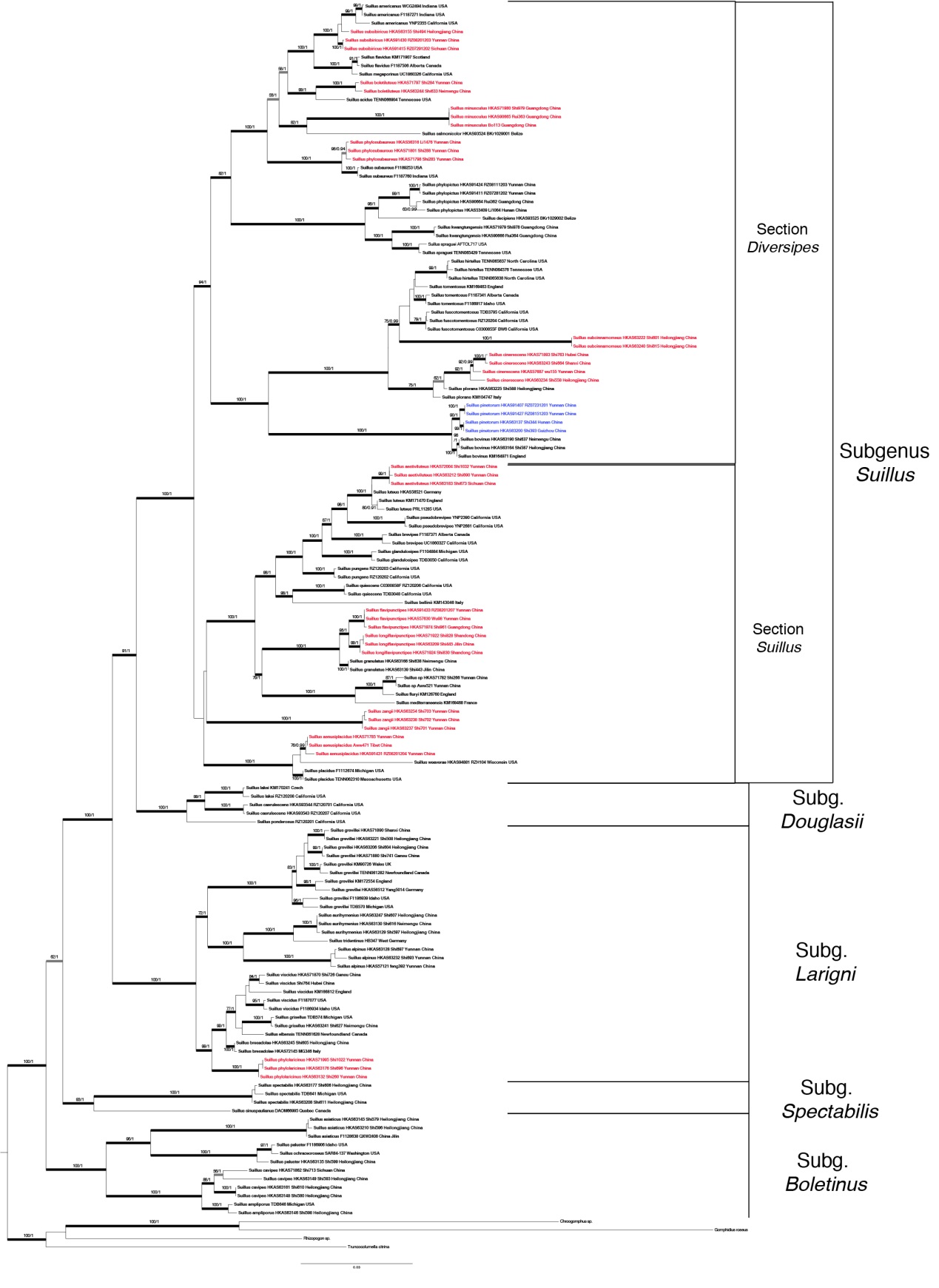


**Fig. S1**. Phylogenetic tree of RAxML and Bayesian analyses of genus *Suillus* based on the concatenated datasets of ITS, LSU, *TEFα-1, RPB1* and *RPB2* loci. Topological structures of RAxML and Bayesian analyses are without supported conflicts. Thick and black branches indicate ML bootstrap >70 % and Bayesian posterior probabilities >0.95, written close to the branches as “ML / Bayesian”. Thick and grey bars indicate >70 % ML bootstrap or >0.95 Bayesian PP. Subgenera and sections are annotated in the right side with line blocks to indicate species included. Bold letters indicate sequences generated in this project. Red letters are for new species described in the study, blue letters are for the redefined *S. pinetorum*. *Chroogomphus* sp., *Gomphidius* *roseus*, *Rhizopogon* sp. and *Truncocolumella citrina* were as outgroups.

**
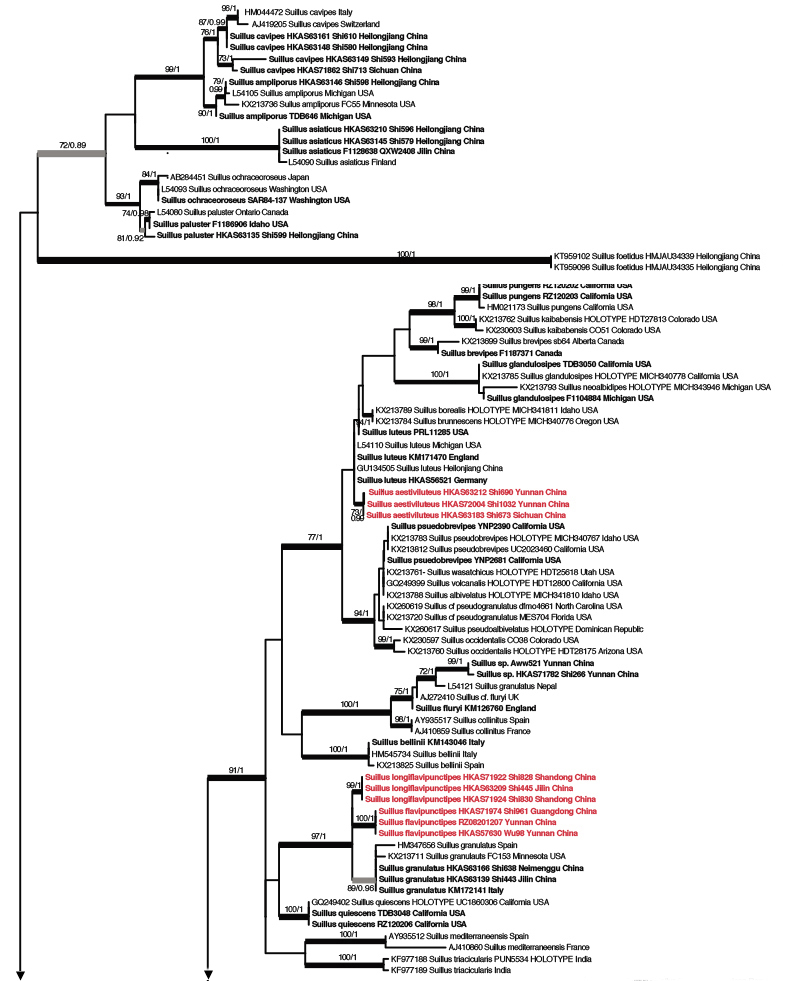
**

**
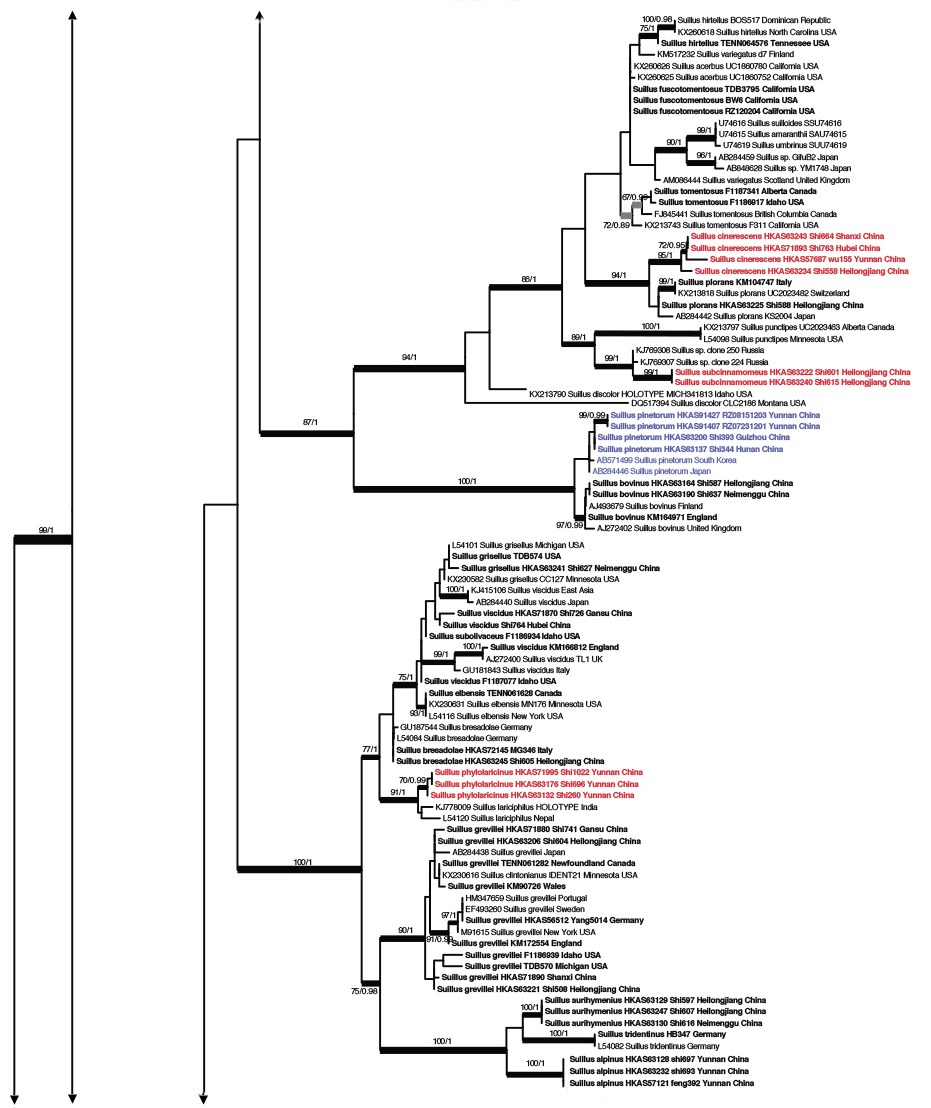

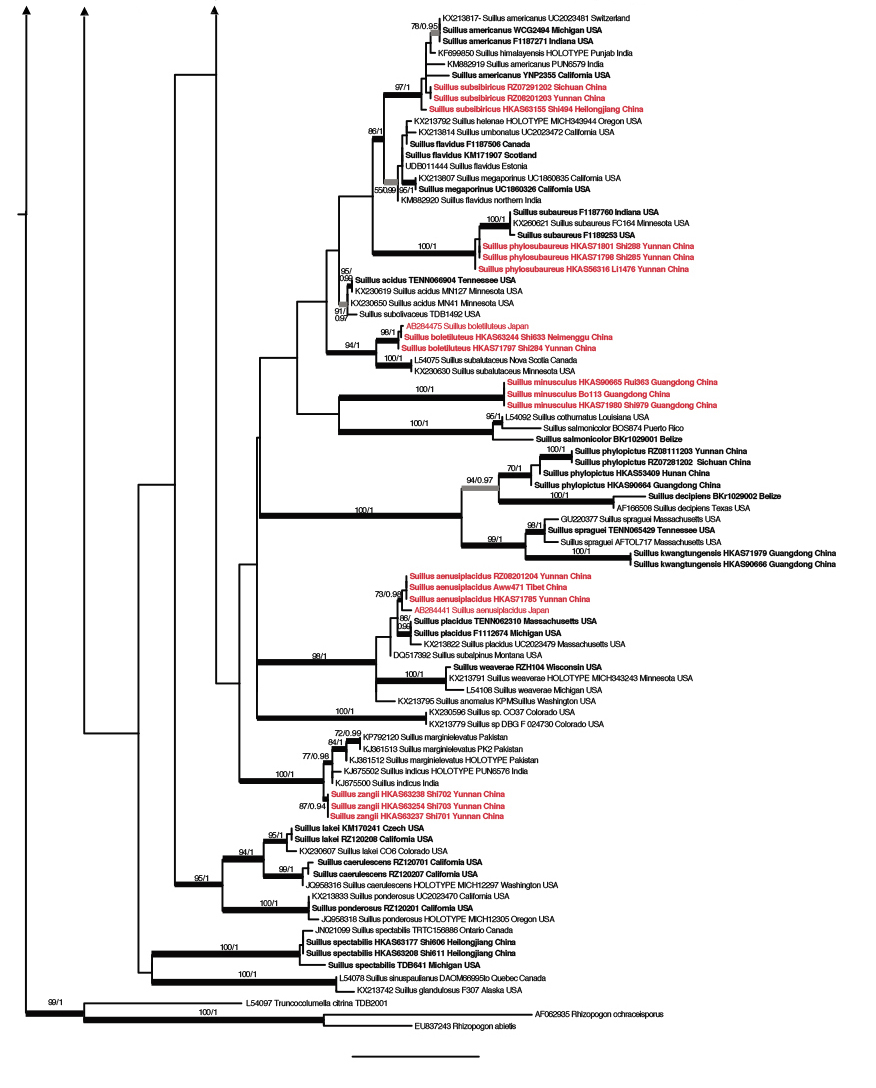
**

**Fig. S2** Phylogenetic tree of RAxML and Bayesian analyses of genus *Suillus* based on ITS sequences. Topological structures of RAxML and Bayesian analyses are statistically congruent. Thick and black branches indicate ML bootstrap >70 % and Bayesian posterior probabilities >0.95, written close to the branches as “ML / Bayesian”. Thick and grey bars indicate >70 % ML bootstrap or >0.95 Bayesian PP. Bold letters indicate sequences generated in this project, others are downloaded from GenBank. Red letters are for new species described in the study, blue letters are for the redefined *S. pinetorum*. *Rhizopogon abietis*, *Rhizopogon ochraceisporus* and *Truncocolumella citrina* were as outgroups.


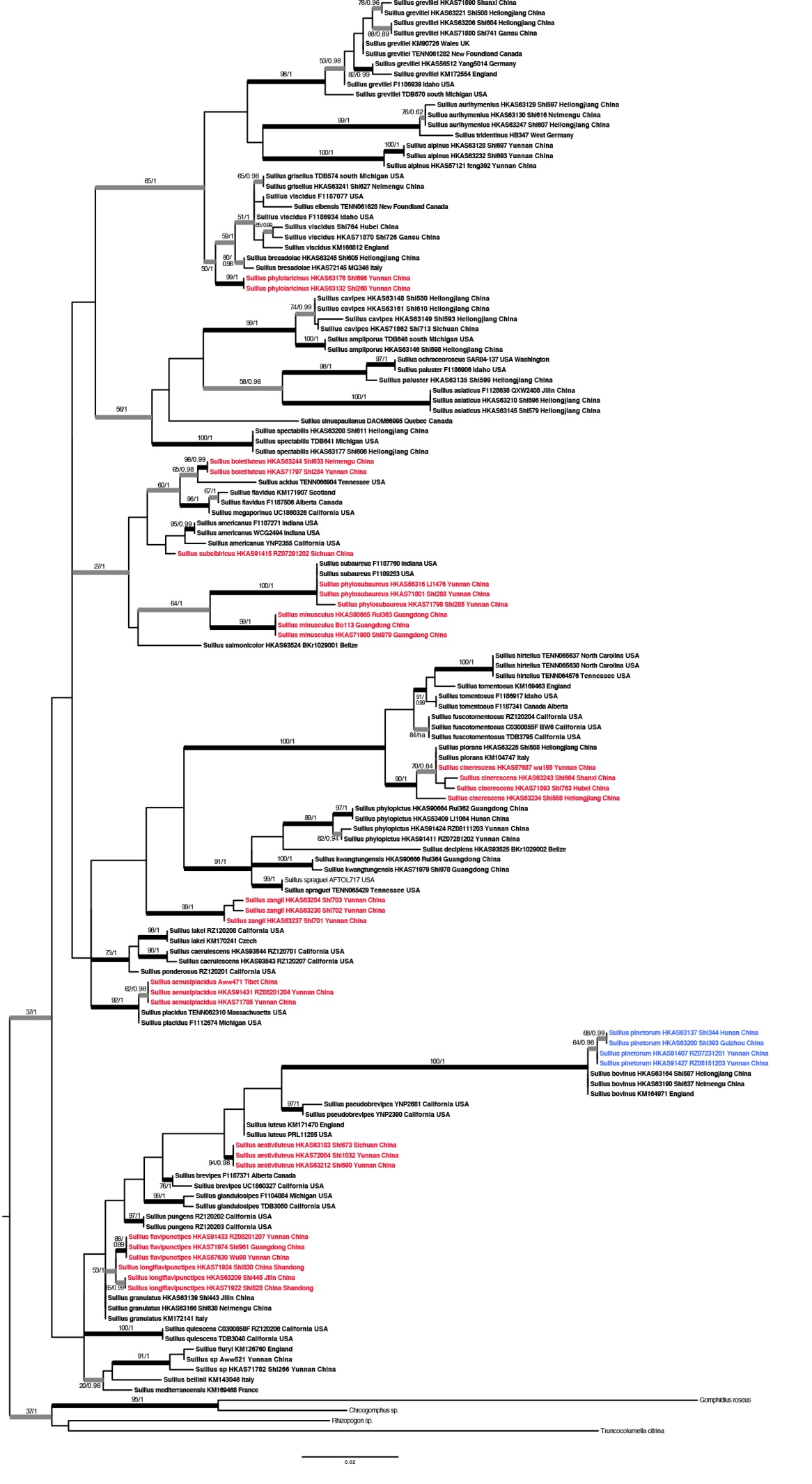


**Fig. S3** Phylogenetic tree of RAxML and Bayesian analyses of genus *Suillus* based on LSU sequences. Topological structures of RAxML and Bayesian analyses are statistically congruent. Thick and black branches indicate ML bootstrap >70 % and Bayesian posterior probabilities >0.95, written close to the branches as “ML / Bayesian”. Thick and grey bars indicate >70 % ML bootstrap or >0.95 Bayesian PP. Bold letters indicate sequences generated in this project. Red letters are for new species described in the study, blue letters are for the redefined *S. pinetorum*. *Chroogomphus* sp., *Gomphidius roseus*, *Rhizopogon* sp. and *Truncocolumella citrina* were as outgroups.


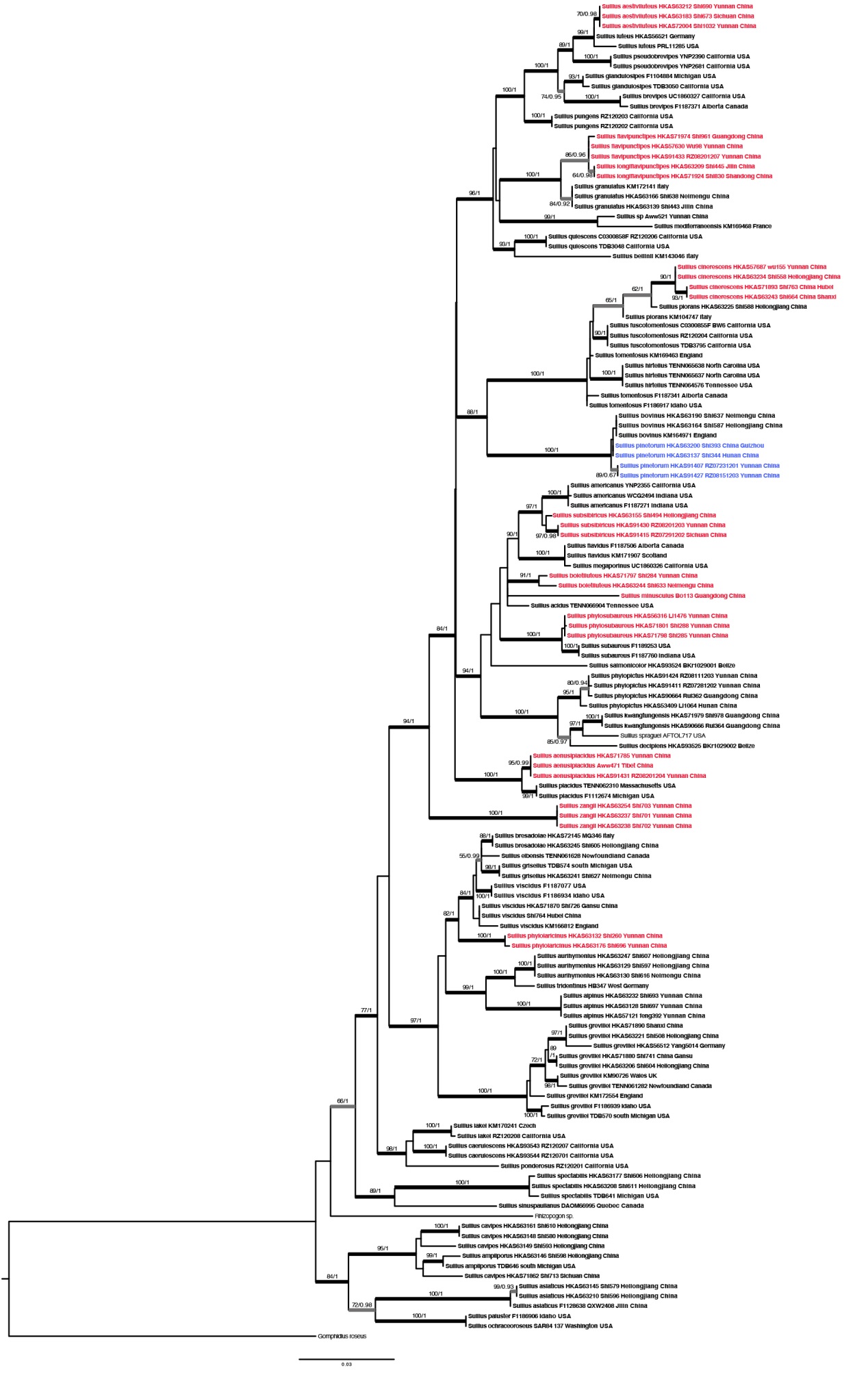


**Fig. S4** Phylogenetic tree of RAxML and Bayesian analyses of genus *Suillus* based on *TEF1-α* sequences. Topological structures of RAxML and Bayesian analyses are statistically congruent. Thick and black branches indicate ML bootstrap >70 % and Bayesian posterior probabilities >0.95, written close to the branches as “ML / Bayesian”. Thick and grey bars indicate >70 % ML bootstrap or >0.95 Bayesian PP. Bold letters indicate sequences generated in this project. Red letters are for new species described in the study, blue letters are for the redefined *S. pinetorum*. *Gomphidius roseus* and *Rhizopogon* sp. were as outgroups.


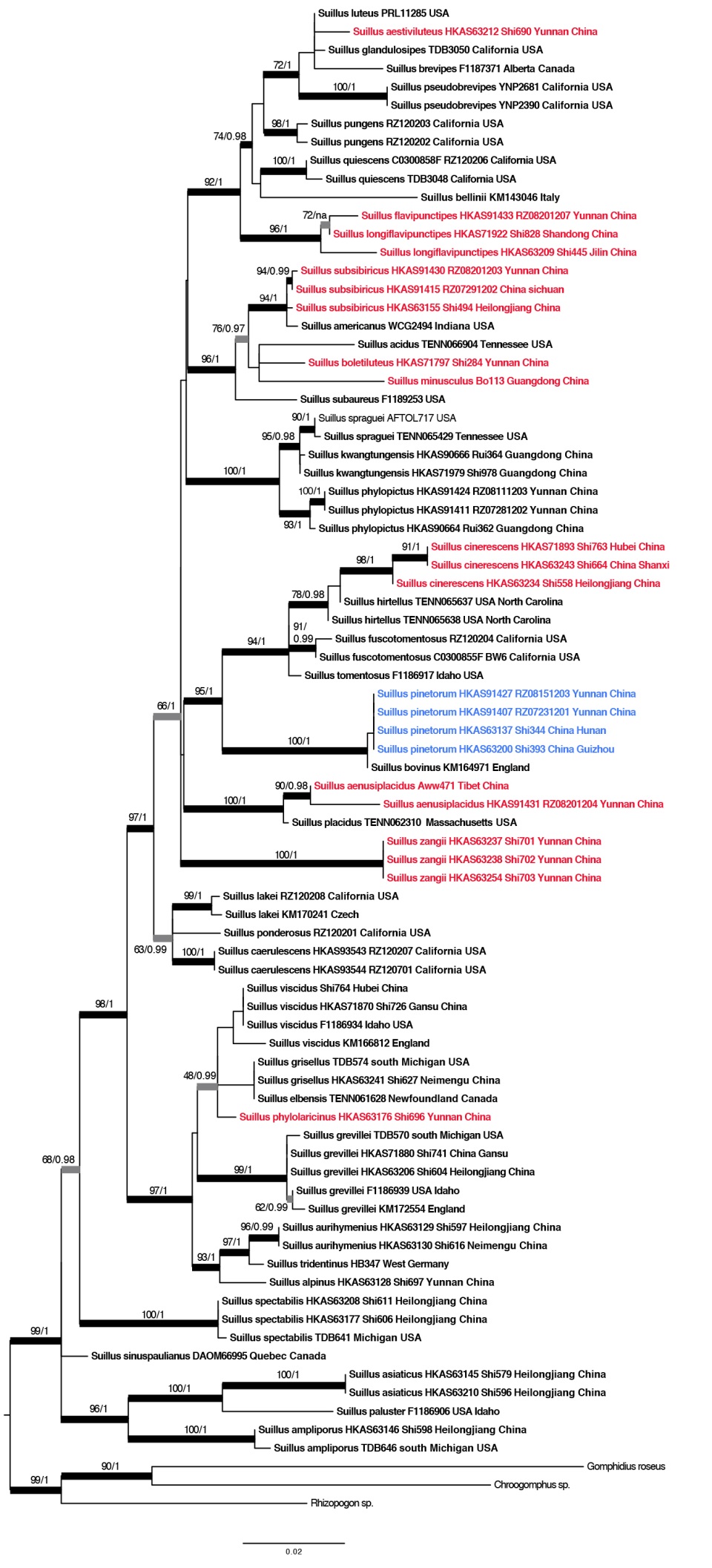


**Fig. S5** Phylogenetic tree of RAxML and Bayesian analyses of genus *Suillus* based on *RPB1* sequences. Topological structures of RAxML and Bayesian analyses are statistically congruent. Thick and black branches indicate ML bootstrap >70 % and Bayesian posterior probabilities >0.95, written close to the branches as “ML / Bayesian”. Thick and grey bars indicate >70 % ML bootstrap or >0.95 Bayesian PP. Bold letters indicate sequences generated in this project. Red letters are for new species described in the study, blue letters are for the redefined *S. pinetorum*. *Chroogomphus* sp., *Gomphidius roseus* and *Rhizopogon* sp. were as outgroups.


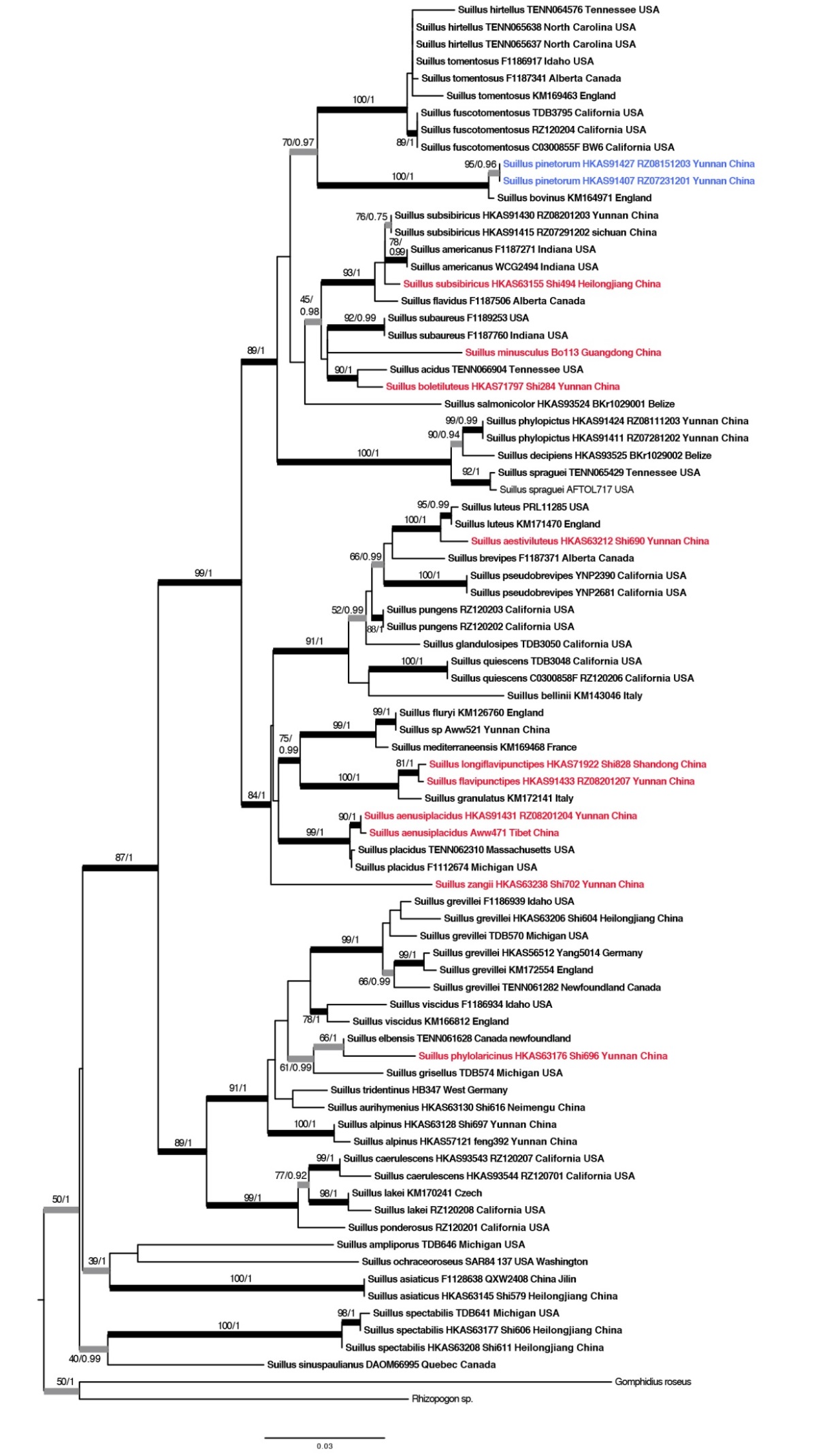


**Fig. S6** Phylogenetic tree of RAxML and Bayesian analyses of genus *Suillus* based on *RPB2* sequences. Topological structures of RAxML and Bayesian analyses are statistically congruent. Thick and black branches indicate ML bootstrap >70 % and Bayesian posterior probabilities >0.95, written close to the branches as “ML / Bayesian”. Thick and grey bars indicate >70 % ML bootstrap or >0.95 Bayesian PP. Bold letters indicate sequences generated in this project. Red letters are for new species described in the study, blue letters are for the redefined *S. pinetorum*. *Gomphidius roseus* and *Rhizopogon* sp. were as outgroups.
